# Supplementary material for: A deep learning-based computational pipeline predicts developmental outcome in retinal organoids
Source: PLoS Biol. 2026 Jan 27;24(1):e3003597. doi: 10.1371/journal.pbio.3003597 (PMC12843511; doi:10.1371/journal.pbio.3003597)
Supplement: S1 Note — (DOCX) [file pbio.3003597.s003.docx]

## **S1 Note**

### **Minimal organoid number for prediction tasks**

To examine how many training samples were required to achieve stable prediction performance, we systematically reduced the number of experiments included during model training. Although accurate predictions of both tissue emergence and tissue size were obtained when models were trained with images from about 1,000 organoids, we observed that convolutional neural networks reached a plateau in performance already at around six experiments (corresponding to around 500 organoids). This suggests that further increasing the training set size beyond this point provided only marginal gains in accuracy. By contrast, the machine learning classifiers did not show a clear plateau, indicating that tabular feature–based approaches required fewer experiments to reach comparable accuracy on the test set (**Supplementary Figure S28**).

### **Comparison of attribution methods to explain deep learning model decision making**

In order to find relevant structural information in the images that would guide the deep learning model’s decisions when predicting TOI at early time points before visibility, we analyzed outputs across eight relevance backpropagation methods (compare **Methods**) and all three CNN architectures. We observed notable differences in how relevance was assigned and how consistently these assignments aligned. Pairwise comparisons within the same model showed that most methods shared little overlap, with Dice coefficients remaining close to baseline levels throughout the time course (**Supplementary Figure S29-S33**). An exception was the combination of Grad-CAM and Guided Grad-CAM, which consistently exhibited higher agreement, indicating that CAM-based methods identify similar regions relative to gradient- or perturbation-based approaches. When comparing the same saliency method across different CNN architectures, gradient-based approaches such as DeepLiftSHAP and Integrated Gradients achieved the highest and most stable correlations, while CAM- and perturbation-based methods were less consistent across models (**Supplementary Figure S34**). Entropy analyses revealed that Grad-CAM and guided Grad-CAM consistently showed the lowest entropy and became progressively more focused over time, while feature ablation and kernel SHAP also displayed decreasing entropy in several readouts. In contrast, gradient-based methods (DeepLIFT SHAP, integrated gradients, simple saliency) and smooth occlusion maintained higher and more stable entropy values, indicating broader and more diffuse saliency distributions (**Supplementary Figure S35**). Finally, regional voting analyses showed that overlap across methods was highest at early time points, with multiple methods frequently labelling the same regions, but this agreement decreased as organoid development progressed (**Supplementary Figure S36**). Drift analyses of saliency map centers of mass further demonstrated that CAM-based (Grad-CAM, Guided Grad-CAM) and perturbation-based methods (Feature Ablation, Kernel SHAP) produced the strongest displacement over time, while gradient-based methods and smooth occlusion remained more stable (**Supplementary Figure S37**). Taken together, these results suggest that saliency methods differ not only in the regions they prioritize but also in their robustness across models, with CAM-based methods focusing relevance more narrowly and consistently within their family, and gradient-based methods maintaining broader, more transferable relevance patterns.

Overall, we were not able to identify comprehensible organoid features with relevance backpropagation that would explain the decision making of the CNN and give further unequivocal insights into early indicators of organoid morphology for the development of the TOI.
