# Supplementary figures and images for: A deep learning-based computational pipeline predicts developmental outcome in retinal organoids
Source: PLoS Biol. 2026 Jan 27;24(1):e3003597. doi: 10.1371/journal.pbio.3003597 (PMC12843511; doi:10.1371/journal.pbio.3003597)

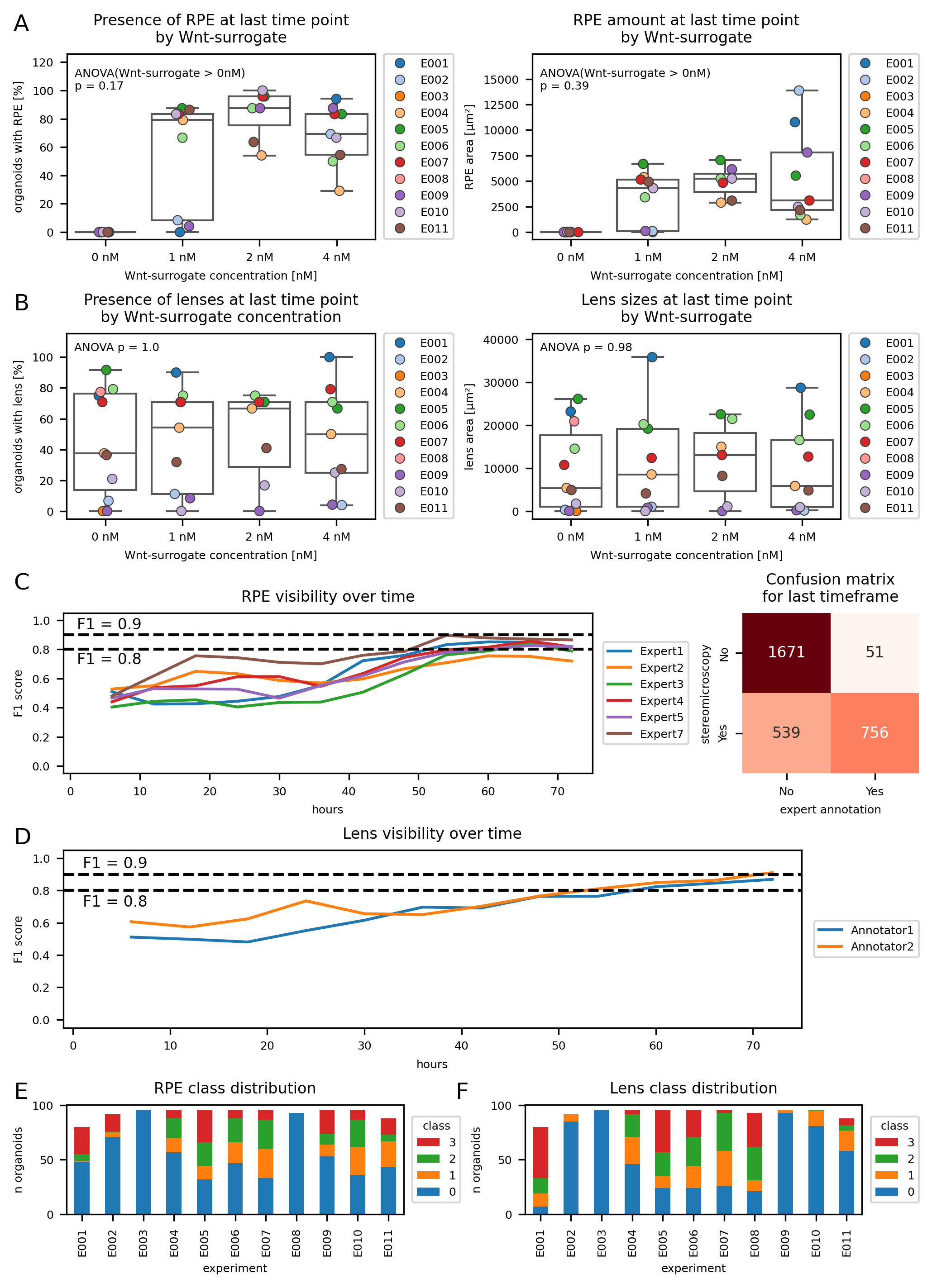

Supplement: S1 Fig — A Development of retinal pigmented epithelium (RPE) and its amount by Wnt-surrogate concentration. Organoids were treated with the indicated concentrations of Wnt-surrogate and subjected to stereomicroscopy in order to detect the presence of RPE (left graph) and to measure its area (right graph). RPE development was observable after Wnt-surrogate treatment but absent in non-treated organoids over all conducted experiments. Notably, there was no consistent correlation of the concentration of the inducing agent and the rate (left graph) or amount (right graph) of RPE induction. Raw data of the figure plots have been deposited as Extended Data 9. B Development of lenses and their sizes by Wnt-surrogate concentration. Organoids were treated with the indicated concentrations of Wnt-surrogate. Lenses were detected from the time-lapse widefield microscopy images (left graph) and the area was measured (right graph). Lens development and lens sizes were found to be independent of the Wnt-surrogate-treatment and -concentration. The indicated p-values were derived from a one-way ANOVA over all four groups. Data points are color coded for the respective experiments. Raw data of the figure plots have been deposited as Extended Data 9. C RPE visibility over time and increased sensitivity of RPE detection by stereomicroscopy. Expert evaluation of the presence of RPE in each organoid over time (see Methods). F1-scores were calculated using the stereomicroscopy annotation as ground truth. RPE begins to visibly emerge around 68 h of organoid development. There is a notable difference between the stereomicroscopy derived ground truth and the human evaluation of the time-lapse images at later time points, suggesting an increased sensitivity of stereomicroscopy over the time-lapse images for the detection of visible RPE. The confusion matrix (right panel), summed over the last 6 hours of the imaging window, confirms the inferior sensitivity of human annotation from time-lapse images com [file pbio.3003597.s004.tif]

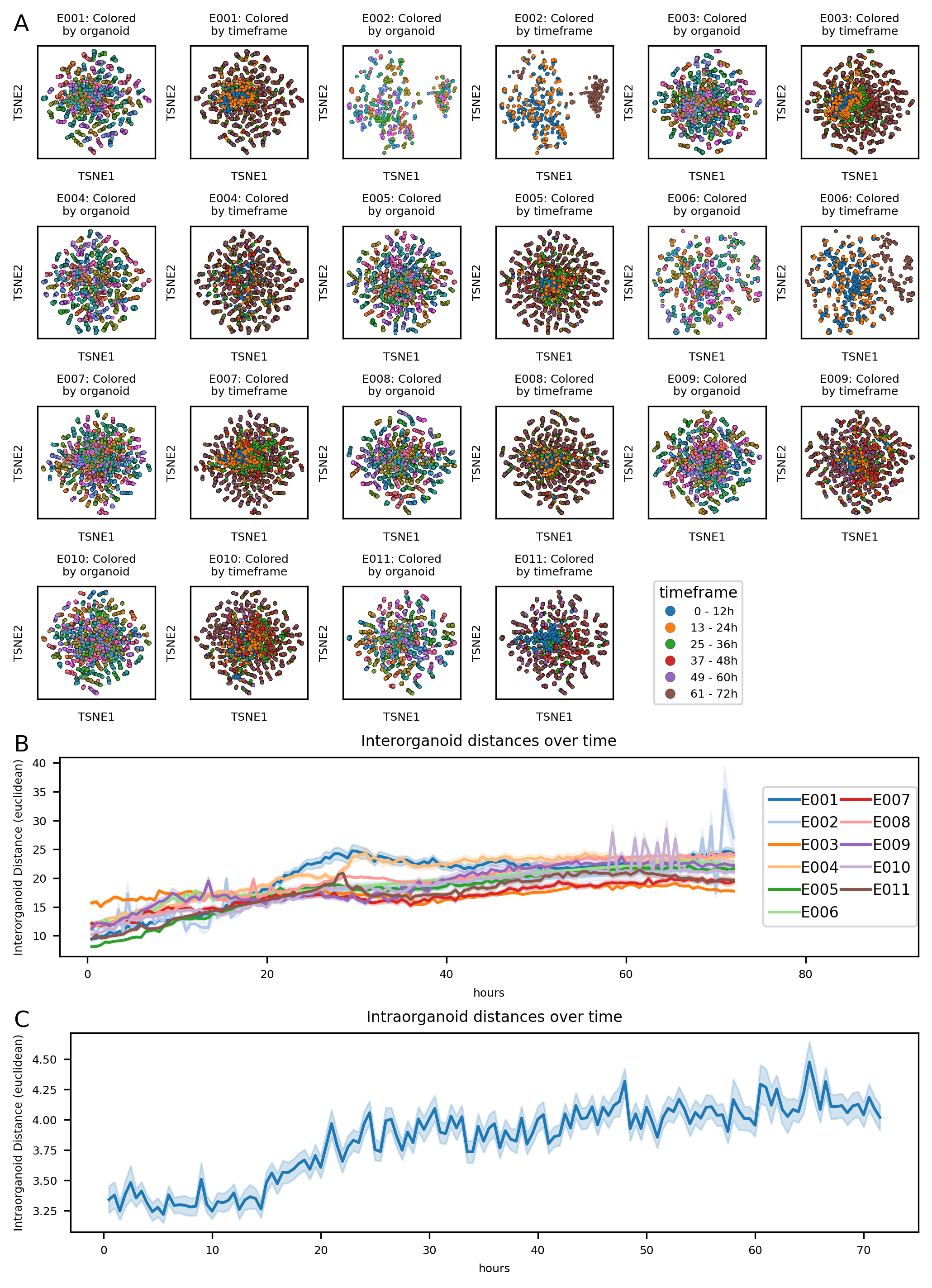

Supplement: S2 Fig — A Retinal organoid images were analyzed using the image analysis platform (see Fig 2 and Methods). Data of the indicated experiments (E0XX) were subjected to t-SNE dimensionality reduction of the first 20 principal components obtained from the morphometrics data and colored by organoid-identity (respective left graph) and the time of acquisition (respective right graph). While the data points of organoid images from earlier time points cluster more closely, there is a substantial divergence of organoids at later time points, suggesting increasing inter-individual differences of the morphologic characteristics over time. Raw data of the figure plots have been deposited as Extended Data 11. B Global morphological inter-organoid heterogeneity across experiments and time. The data correspond to the data shown in Fig 2D. Here, only organoids without Wnt-surrogate treatment were analyzed. Euclidean distances were calculated based on the first 20 principal components obtained from scaled morphometrics data for each organoid and plotted as the mean pairwise distance between all organoids for each time point. Notably, inter-organoid distance increased over time in an experiment-specific manner, indicating an increasing morphological divergence of the individual organoids over time. Raw data of the figure plots have been deposited as Extended Data 12. C Intra-organoid morphological changes over time. The data correspond to the data shown in Fig 2C. Here, only organoids without Wnt-surrogate treatment were analyzed. Euclidean distances were calculated based on 20 principal components of the morphometrics data for each organoid between time point n and time point n + 1. The resulting metric reflects the amount of morphological changes during a time span of 30 min. While the relative changes are comparatively small at the beginning, the increase over time is suggesting more drastic morphological changes at later time points, consistent with the findings described in B and C. Raw [file pbio.3003597.s005.tif]

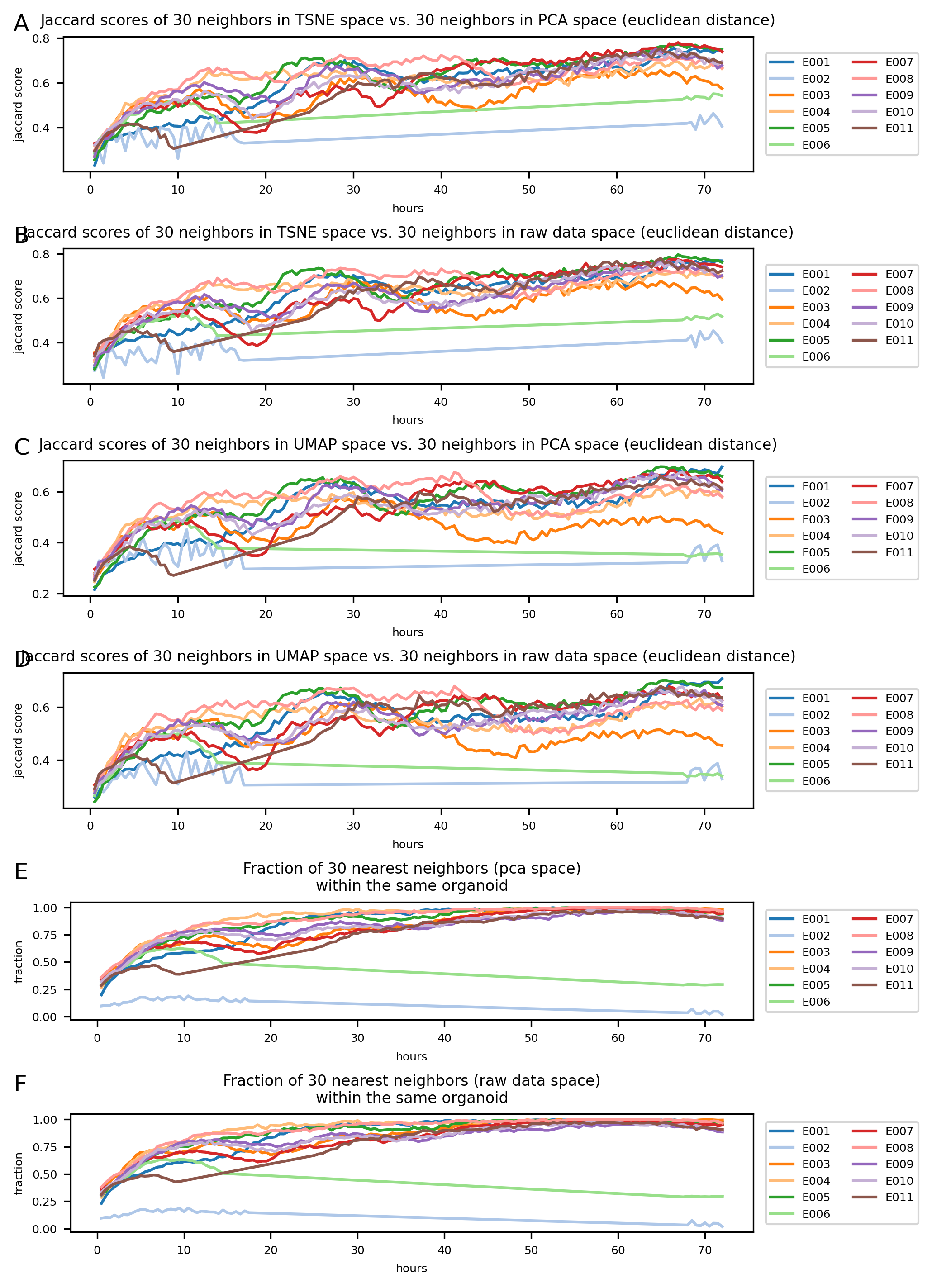

Supplement: S3 Fig — The data directly correspond to Figs 2 and S2. A–D For each data point, 30 nearest neighbors were calculated in PC space (20 principal components derived from the morphometrics data, A/C) or in raw data space (scaled morphometrics data, B/D). Subsequently, 30 nearest neighbors were identified from dimensionality reductions in 2 dimensions (either TSNE or UMAP). The size of the intersection over the size of the union (jaccard index) was calculated over time. At earlier timepoints, jaccard scores are comparably low, confirming the dense cloud of data points and morphological similarity of the organoids. At later timepoints, the jaccard score increases, indicating a good data structure preservation by dimensionality reduction compared to raw distance measurements. Raw data of the figure plots have been deposited as Extended Data 13. E/F For each data point, 30 nearest neighbors have been calculated in either raw data space or PC space as described above. At each time point, the fraction of nearest neighbors corresponding to the same organoid has been quantified. At earlier timepoints, the structural similarity of individual organoids is high as indicated by the low overlap of nearest neighbors derived from the same individual organoid. At later timepoints, the fraction of nearest neighbors derived from the same organoid is almost 1, confirming the visual representation from Figs 2 and S2 and conclusion that organoids at later timepoints are morphologically diverse and more similar within itself than towards other organoids at the same timepoints. Raw data of the figure plots have been deposited as Extended Data 14. (TIF) [file pbio.3003597.s006.tif]

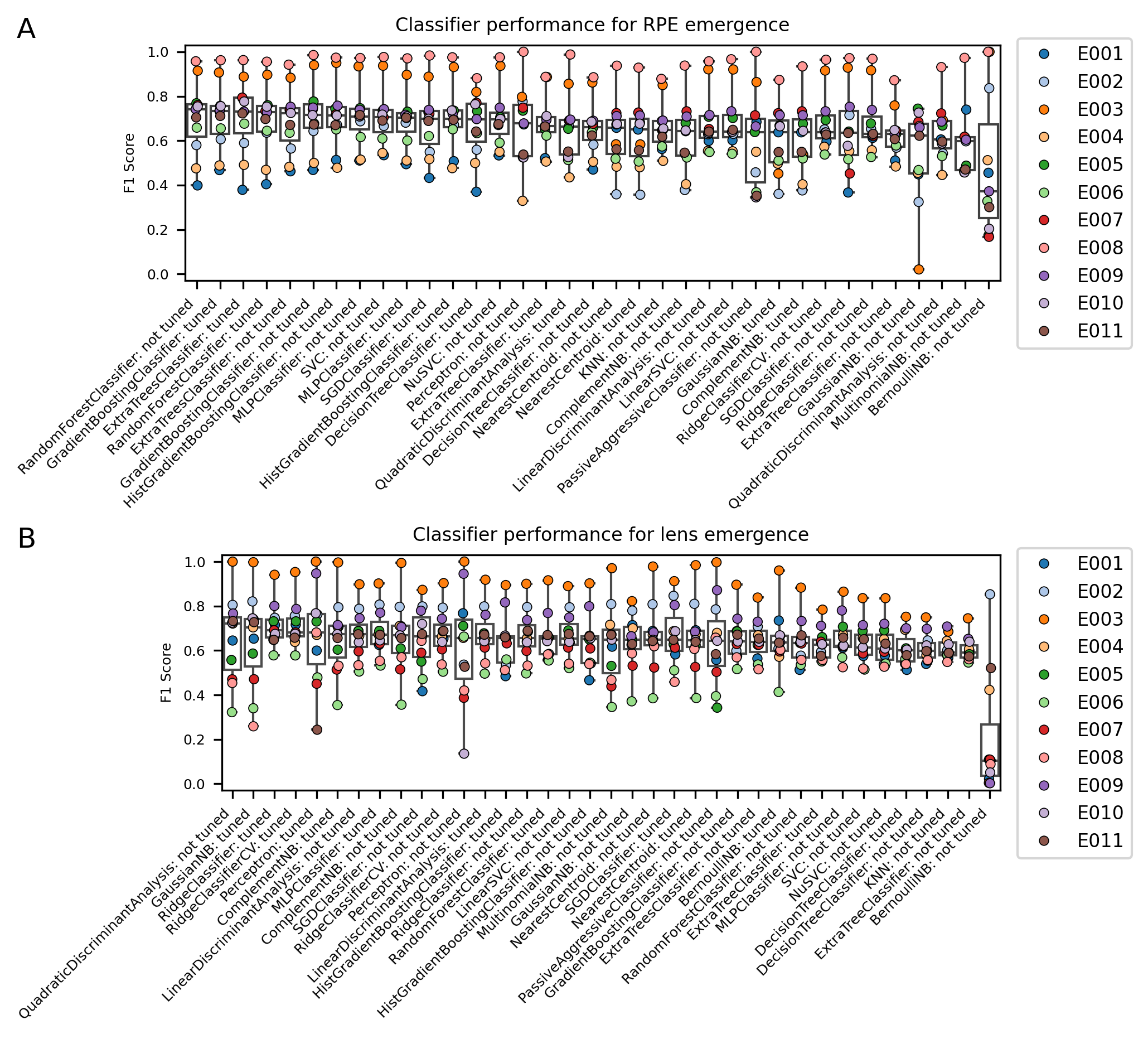

Supplement: S4 Fig — This analysis was performed on morphometrics data from single image slices. A The indicated classifiers were trained by cross-validation, using the indicated experiment as a test set, and scored using the F1 metric (y-axis) for the prediction of the presence and absence of RPE. Selected classifiers were subjected to hyperparameter tuning first (tuned). Raw data of the figure plots have been deposited as Extended Data 15. B The indicated classifiers were trained and evaluated as in A, but for the emergence of lenses. Raw data of the figure plots have been deposited as Extended Data 16. (TIF) [file pbio.3003597.s007.tif]

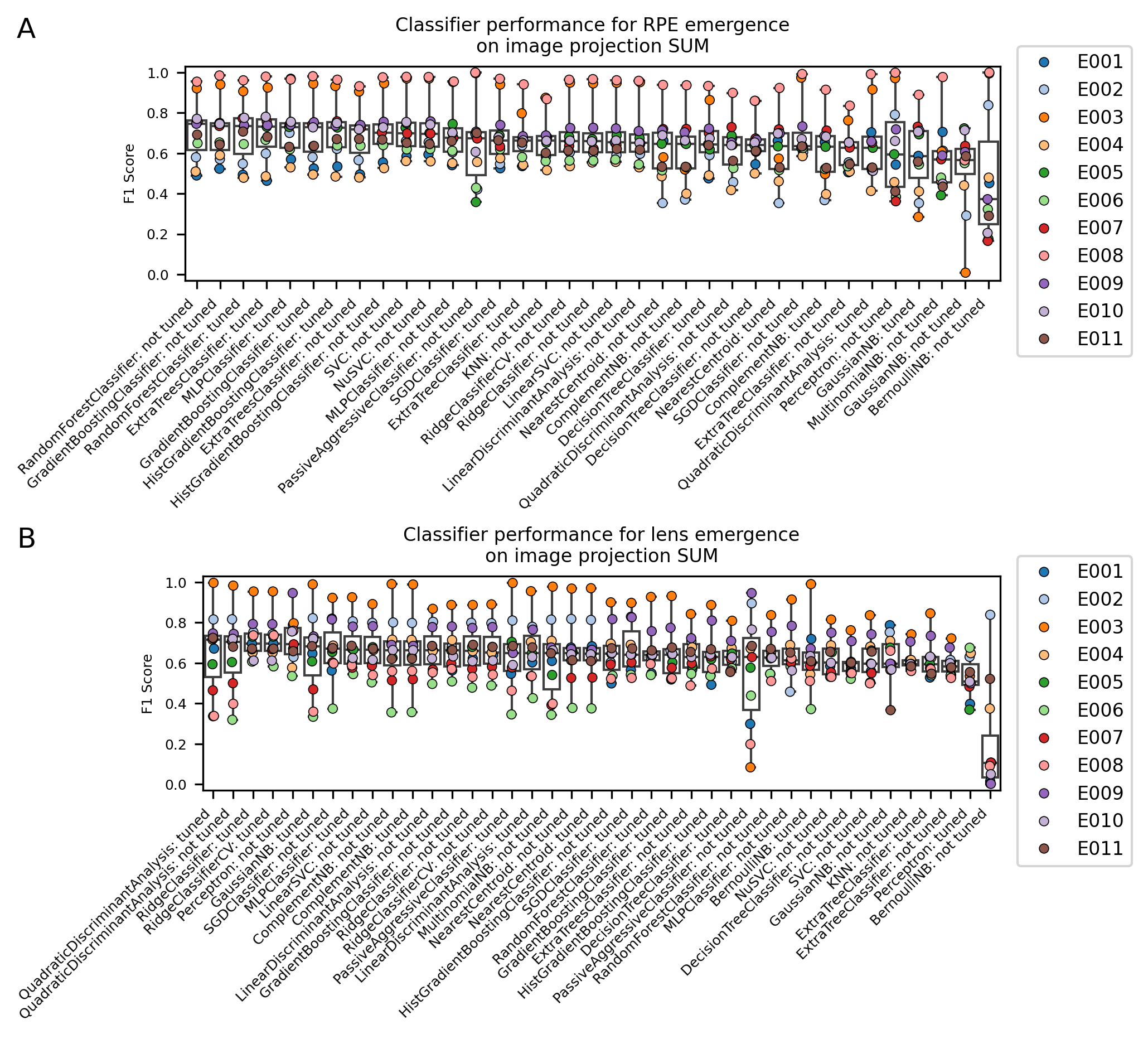

Supplement: S5 Fig — This analysis was performed on morphometrics data from sum-intensity z-projections of all acquired 5 image slices. A The indicated classifiers were trained by cross-validation, using the indicated experiment as a test set, and scored using the F1 metric (y-axis) for the prediction of the presence and absence of RPE. Selected classifiers were subjected to hyperparameter tuning first (tuned). Raw data of the figure plots have been deposited as Extended Data 17. B The indicated classifiers were trained and evaluated as in A, but for the emergence of lenses. Raw data of the figure plots have been deposited as Extended Data 18. (TIF) [file pbio.3003597.s008.tif]

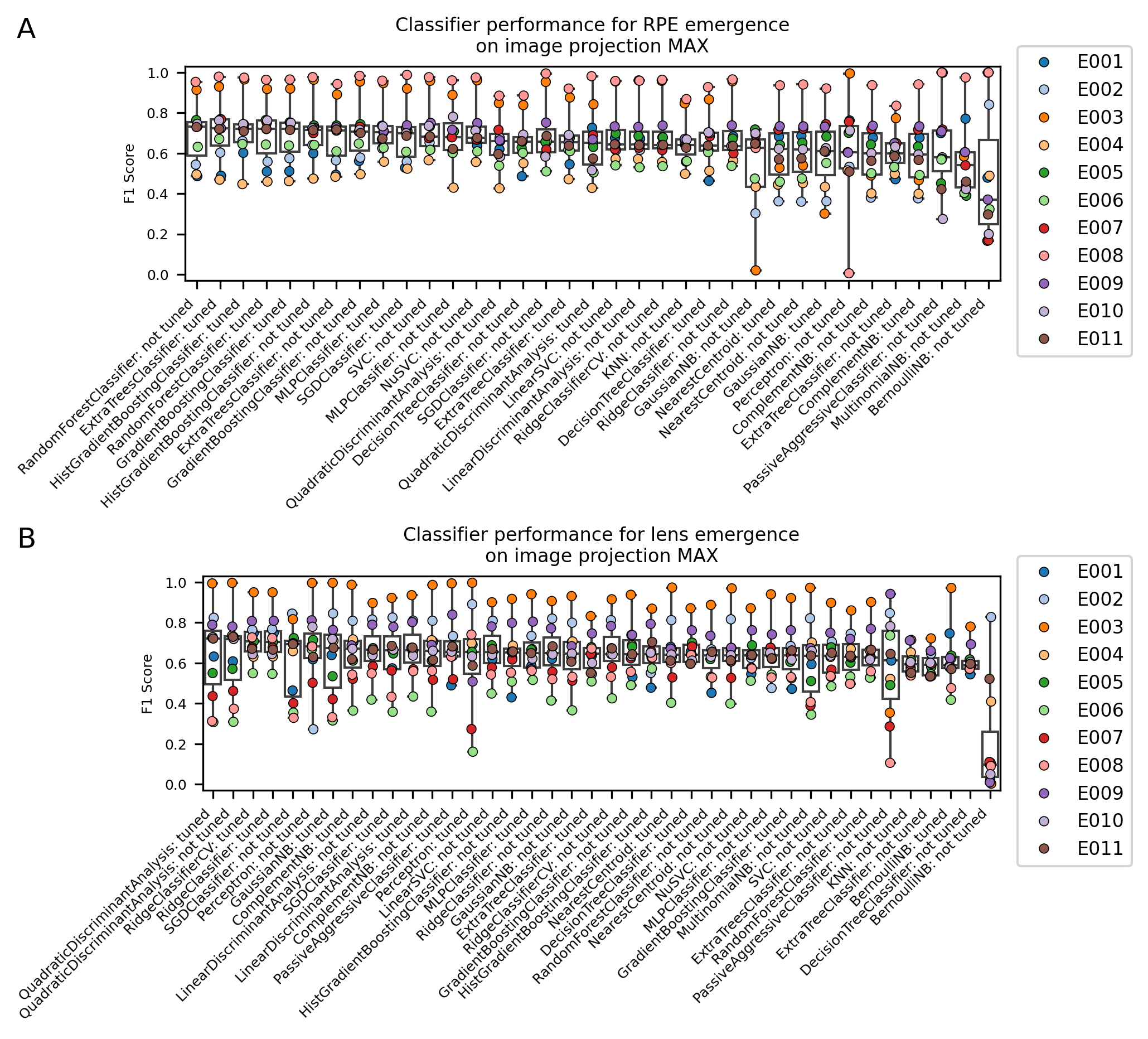

Supplement: S6 Fig — This analysis was performed on morphometrics data from maximum-intensity z-projections of all 5 image slices. A The indicated classifiers were trained by cross-validation, using the indicated experiment as a test set, and scored using the F1 metric (y-axis) for the prediction of the presence and absence of RPE. Selected classifiers were subjected to hyperparameter tuning first (tuned). Raw data of the figure plots have been deposited as Extended Data 19. B The indicated classifiers were trained and evaluated as in A, but for the emergence of lenses. Raw data of the figure plots have been deposited as Extended Data 20. (TIF) [file pbio.3003597.s009.tif]

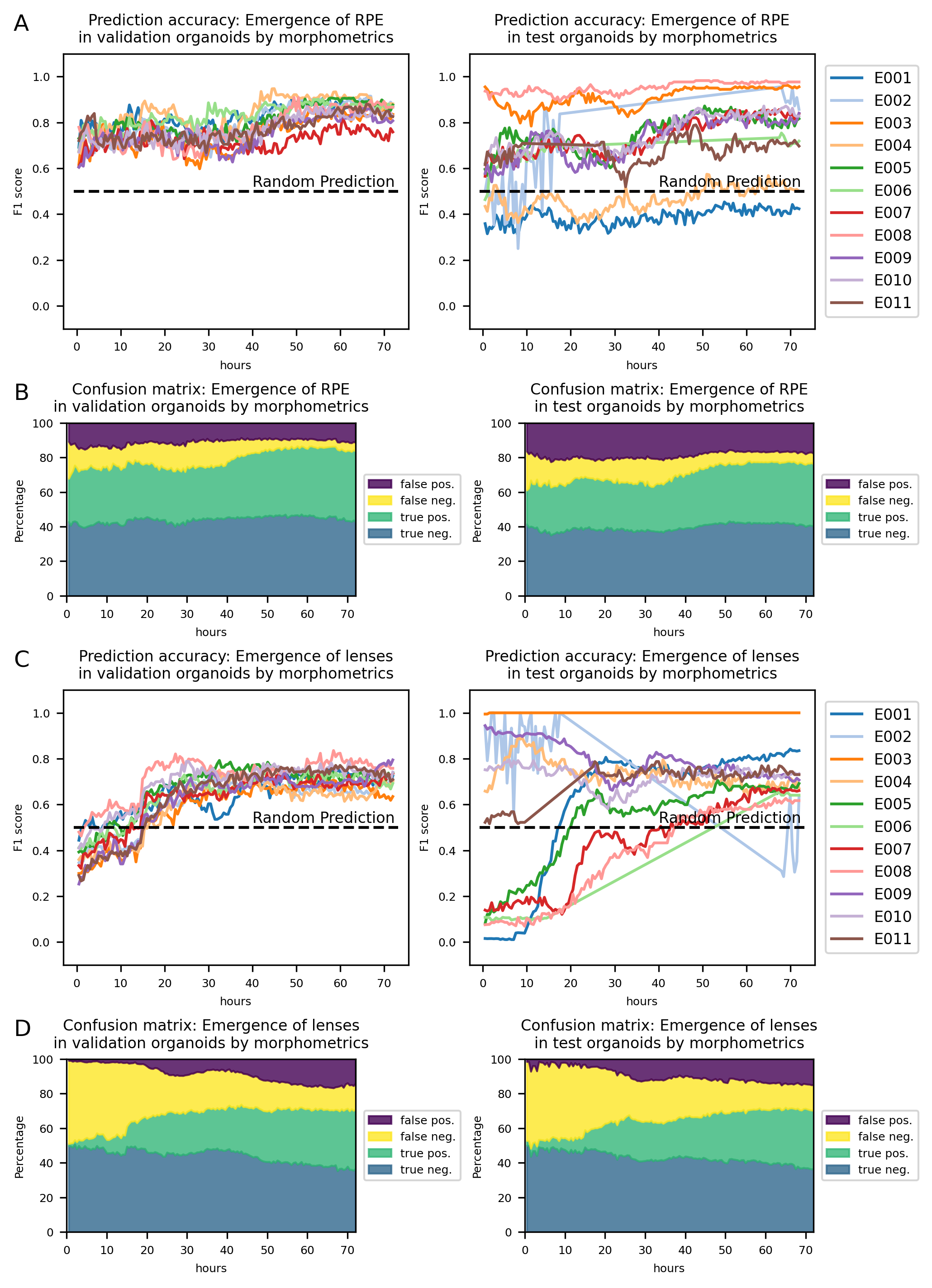

Supplement: S7 Fig — Machine learning classifiers were evaluated on the ability to predict RPE emergence (A, B) and lens emergence (C, D) on the validation (left graph) and test (right graph) data sets (for the data partitioning strategy refer to Fig 3A and Methods). A/C: The data correspond directly to the data shown in Fig 3B (RPE emergence) and Fig 3C (lens emergence) but are split for the individual experiments. Raw data of the figure plots have been deposited as Extended Data 21 and 23, respectively. B/D: Confusion matrices. The data correspond to A and C, respectively. The x-axis denotes the respective imaging time points while the y-axes show the relative percentage of true-positive, true-negative, false-positive and false-negative predictions as indicated. Raw data of the figure plots have been deposited as Extended Data 22 and 24, respectively. Predictions were calculated using the function ‘get_classification_f1_data’ of the module orgAInoid.figures.figure_data_generation (compare source code). (TIF) [file pbio.3003597.s010.tif]

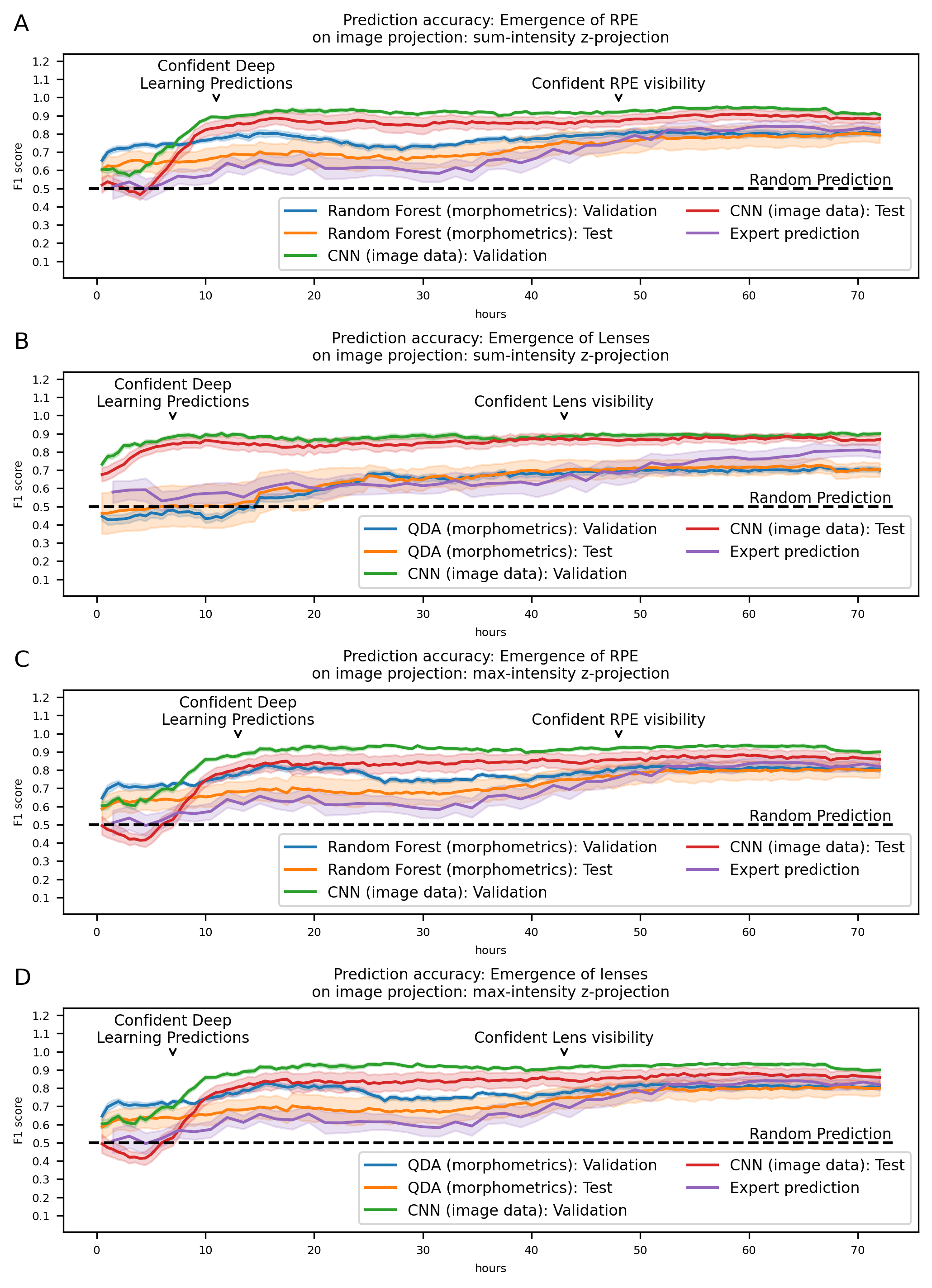

Supplement: S8 Fig — Prediction of RPE emergence (A/C) and lens emergence (B/D) in images generated from sum- (A/B) and maximum-intensity z-projections (C/D). Sum- and maximum-intensity z-projection of the images did not gain significant performance enhancements compared to single-slice analysis (compare to Fig 3). QDA: Quadratic Discriminant Analysis; HGBC: Histogram Gradient Boosting Classifier. Raw data of the figure plots have been deposited as Extended Data 25–28 (A–D). Predictions were calculated using the function ‘get_classification_f1_data’ of the module orgAInoid.figures.figure_data_generation (compare source code). (TIF) [file pbio.3003597.s011.tif]

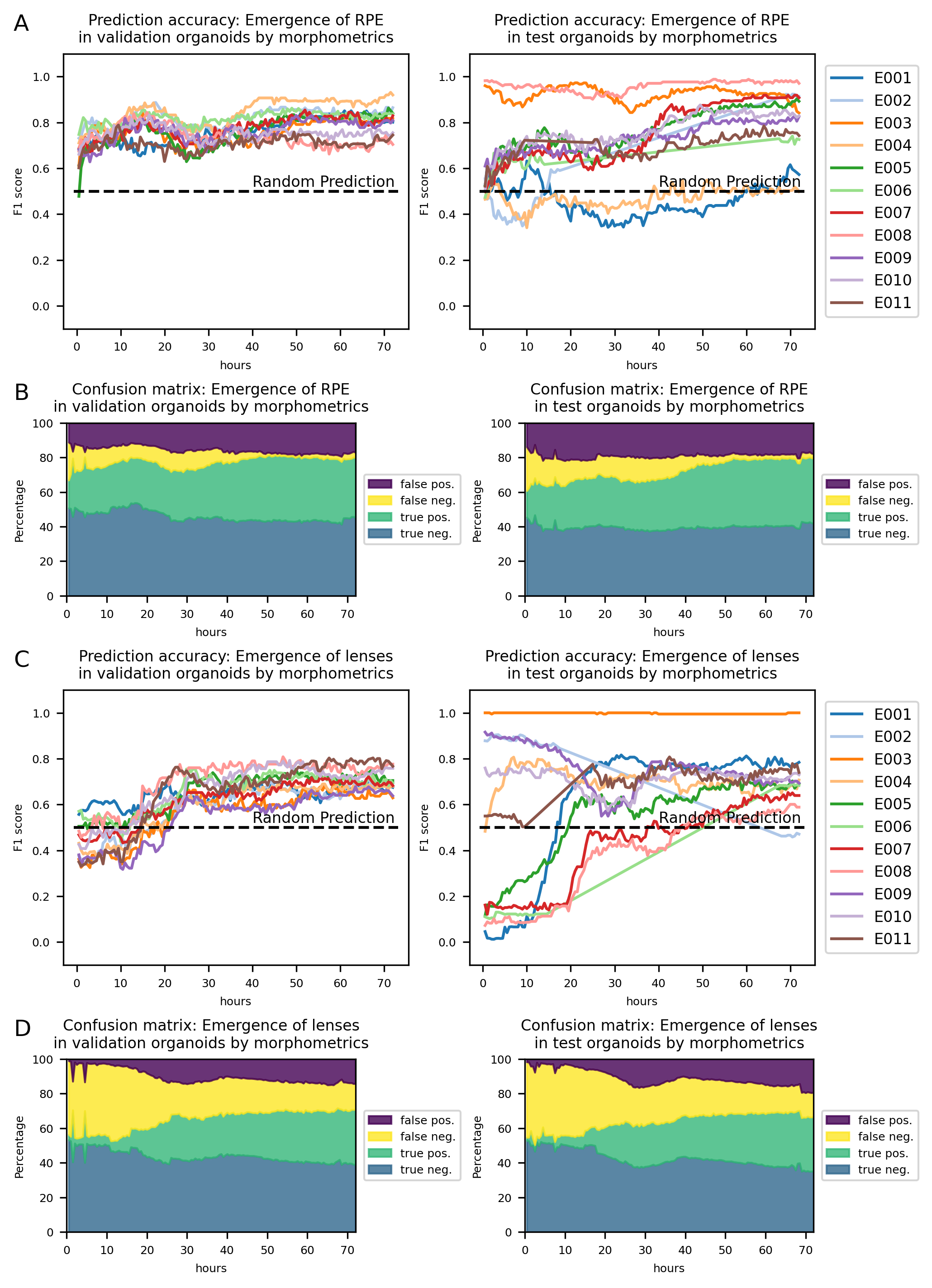

Supplement: S9 Fig — Machine learning classifiers were evaluated on the ability to predict RPE emergence (A, B) and lens emergence (C, D) on the validation (left graph) and test (right graph) data sets (for the data partitioning strategy refer to Fig 3A and Methods). A/C: The data correspond directly to the data shown in S8 Fig but are split for the individual experiments. Raw data of the figure plots have been deposited as Extended Data 29 and 31, respectively. B/D: Confusion matrices. The data correspond to A and C, respectively. The x-axis denotes the respective imaging time points while the y-axes show the relative percentage of true-positive, true-negative, false-positive and false-negative predictions as indicated. Raw data of the figure plots have been deposited as Extended Data 30 and 32, respectively. Predictions were calculated using the function ‘get_classification_f1_data’ of the module orgAInoid.figures.figure_data_generation (compare source code). (TIF) [file pbio.3003597.s012.tif]

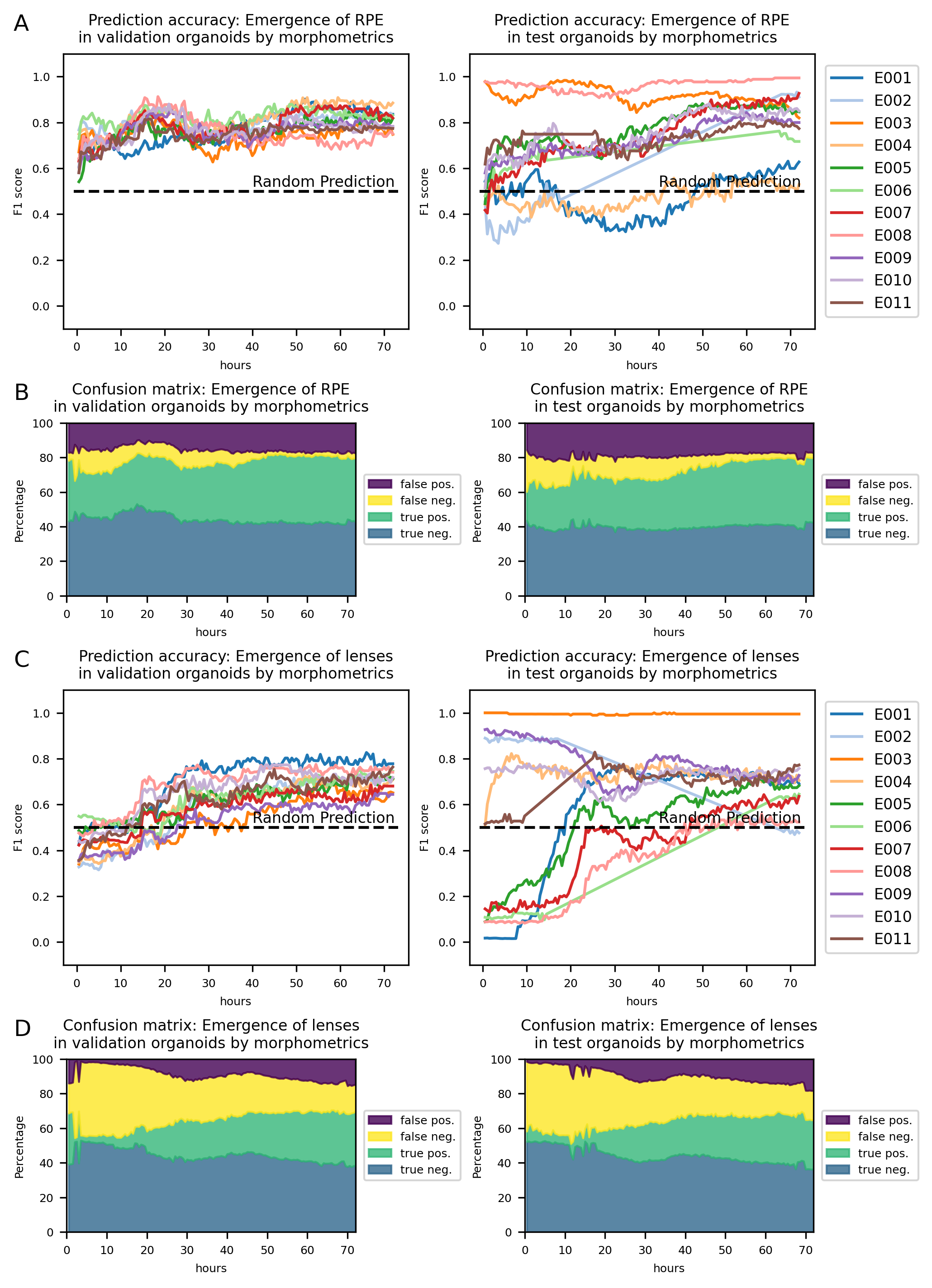

Supplement: S10 Fig — Machine learning classifiers were evaluated on the ability to predict RPE emergence (A, B) and lens emergence (C, D) on the validation (left graph) and test (right graph) data sets (for the data partitioning strategy refer to Fig 3A and Methods). A/C: The data correspond directly to the data shown in S8 Fig but are split for the individual experiments. Raw data of the figure plots have been deposited as Extended Data 33 and 35, respectively. B/D: Confusion matrices. The data correspond to A and C, respectively. The x-axis denotes the respective imaging time points while the y-axes show the relative percentage of true-positive, true-negative, false-positive and false-negative predictions as indicated. Raw data of the figure plots have been deposited as Extended Data 34 and 36, respectively. Predictions were calculated using the function ‘get_classification_f1_data’ of the module orgAInoid.figures.figure_data_generation (compare source code). (TIF) [file pbio.3003597.s013.tif]

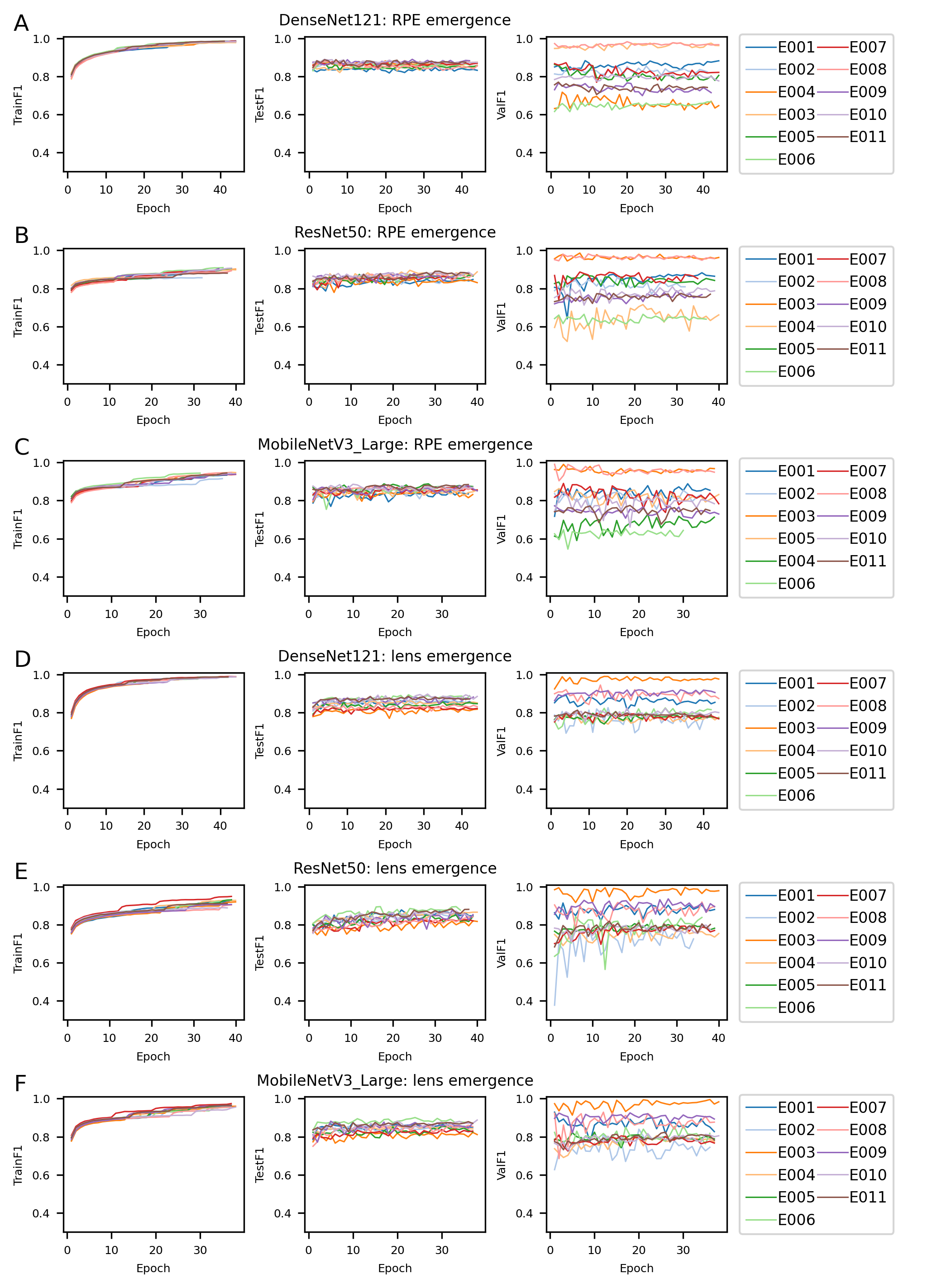

Supplement: S11 Fig — CNNs were trained to predict the emergence of RPE (A–C) and lenses (D–F) from time-lapse single-slice images for the indicated amount of epochs and scored for the F1-metric in the training set (left graph), the validation set (middle graph) and the test set (right graph). The architecture of the respective CNN is noted within the respective title. Raw data of the figure plots have been deposited as Extended Data 37. (TIF) [file pbio.3003597.s014.tif]

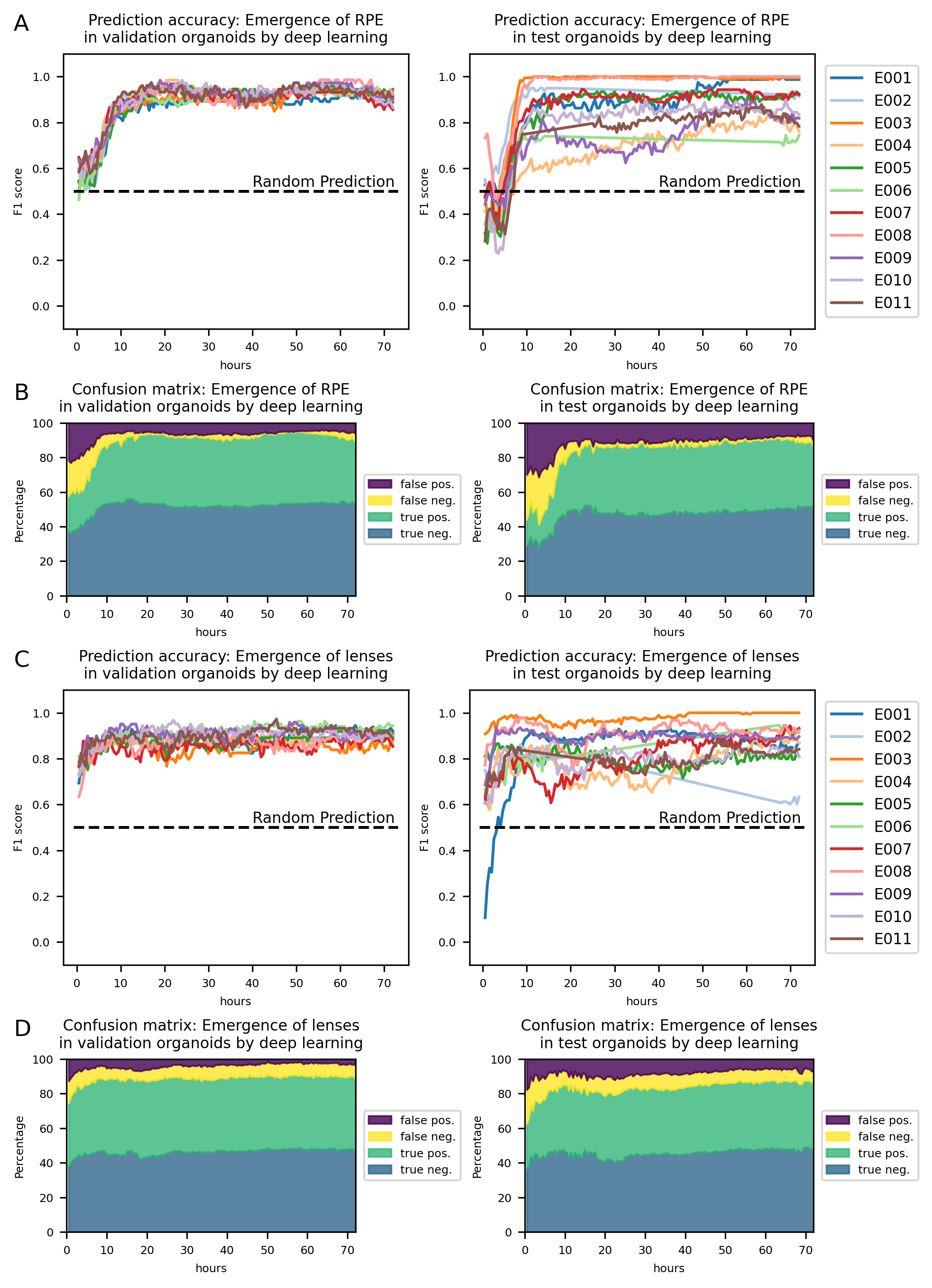

Supplement: S12 Fig — Deep learning classifiers were evaluated on the ability to predict RPE emergence (A, B) and lens emergence (C, D) on the validation (left graph) and test (right graph) data sets (for the data partitioning strategy refer to Fig 3A and Methods). A/C: The data correspond directly to the data shown in Fig 3B (RPE emergence) and Fig 3C (lens emergence) but are split for the individual experiments. Raw data of the figure plots have been deposited as Extended Data 38 and 40, respectively. B/D: Confusion matrices. The data correspond to A and C, respectively. The x-axis denotes the respective imaging time points while the y-axes show the relative percentage of true-positive, true-negative, false-positive and false-negative predictions as indicated. Raw data of the figure plots have been deposited as Extended Data 39 and 41, respectively. Predictions were calculated using the function ‘get_classification_f1_data’ of the module orgAInoid.figures.figure_data_generation (compare source code). (TIF) [file pbio.3003597.s015.tif]

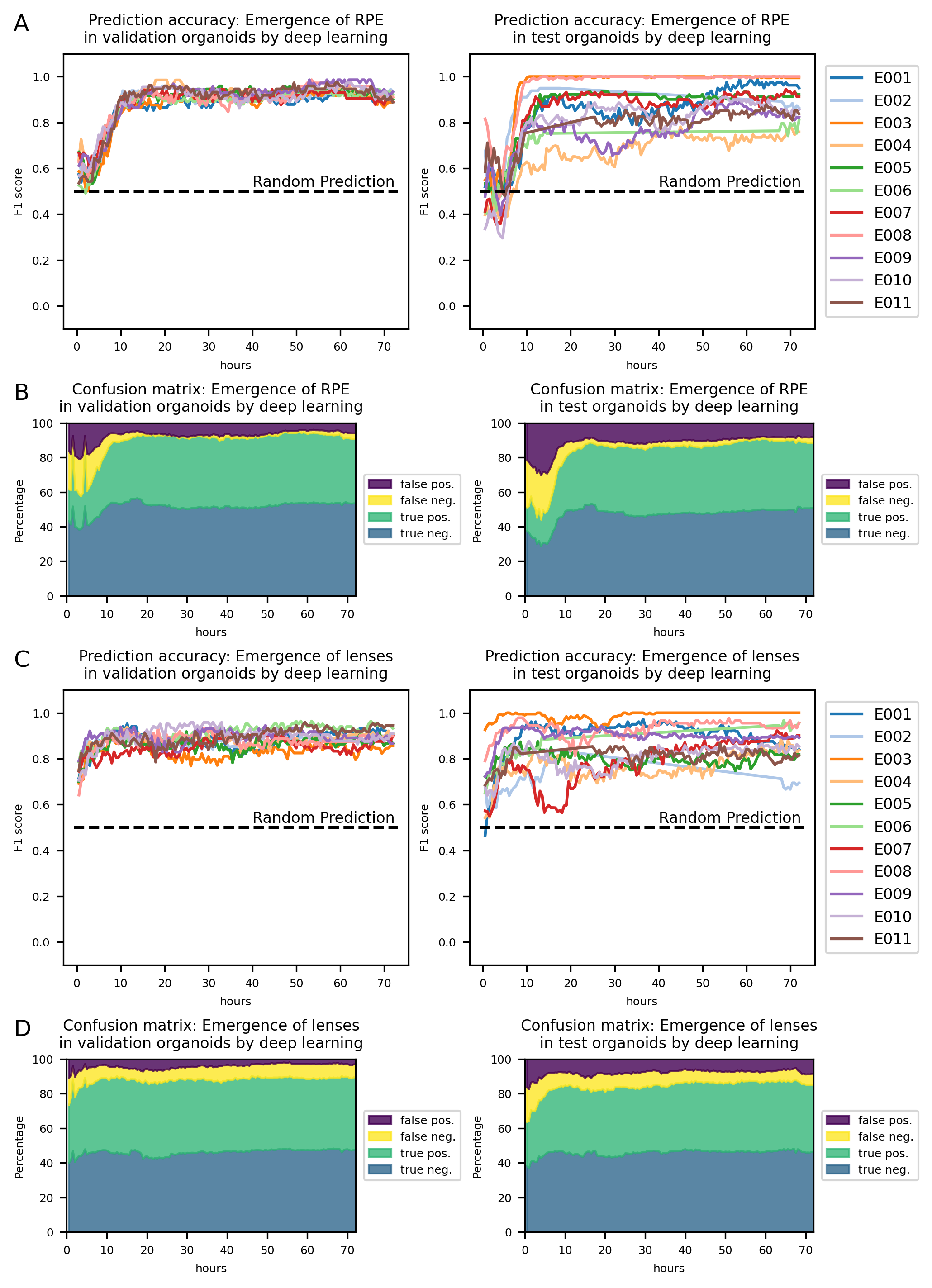

Supplement: S13 Fig — Deep learning classifiers were evaluated on the ability to predict RPE emergence (A, B) and lens emergence (C, D) on the validation (left graph) and test (right graph) data sets (for the data partitioning strategy refer to Fig 3A and Methods). A/C: The data correspond directly to the data shown in S8 Fig, but are split for the individual experiments. Raw data of the figure plots have been deposited as Extended Data 42 and 44, respectively. B/D: Confusion matrices. The data correspond to A and C, respectively. The x-axis denotes the respective imaging time points while the y-axes show the relative percentage of true-positive, true-negative, false-positive and false-negative predictions as indicated. Raw data of the figure plots have been deposited as Extended Data 43 and 45, respectively. Predictions were calculated using the function ‘get_classification_f1_data’ of the module orgAInoid.figures.figure_data_generation (compare source code). (TIF) [file pbio.3003597.s016.tif]

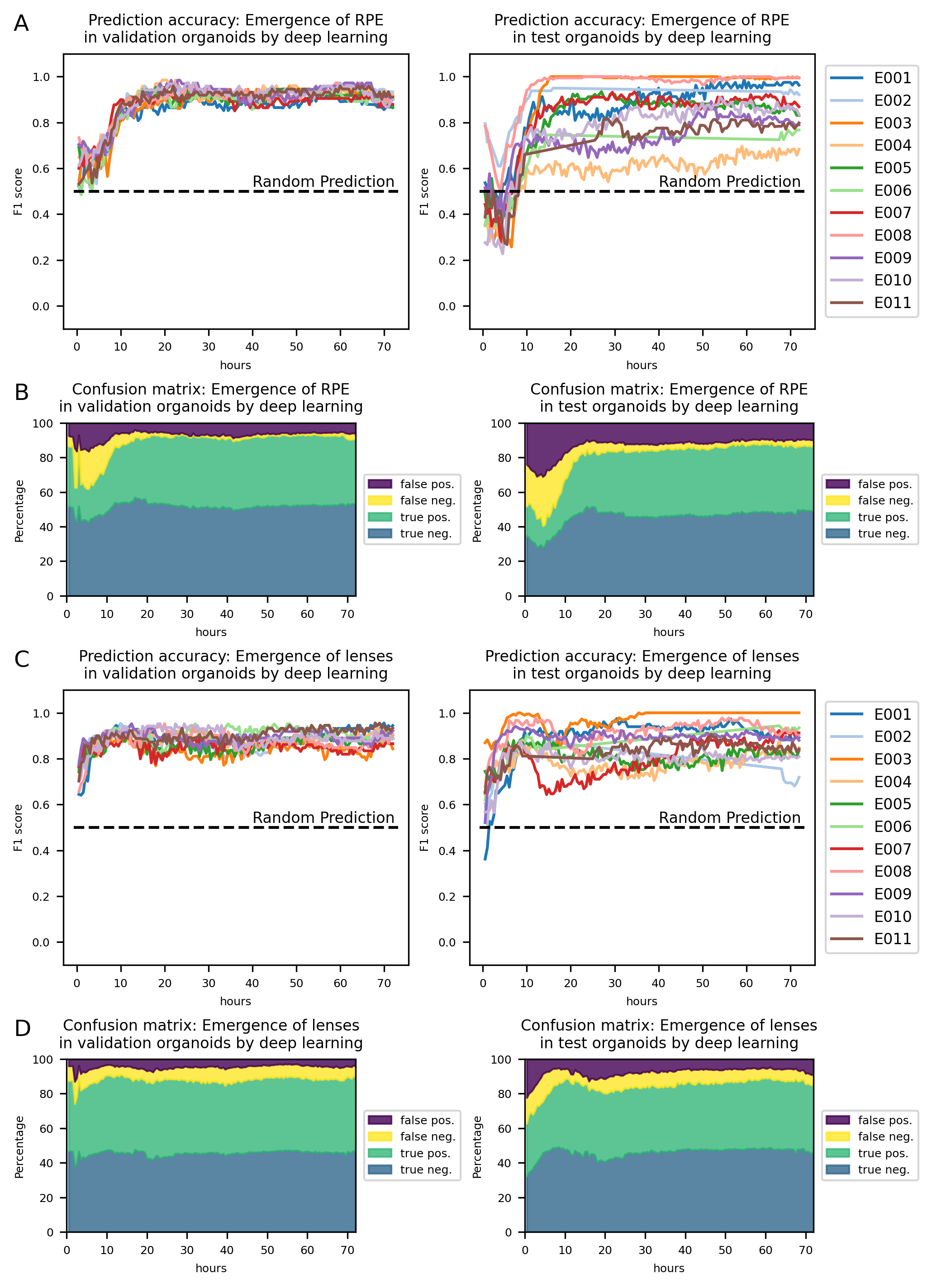

Supplement: S14 Fig — Deep learning classifiers were evaluated on the ability to predict RPE emergence (A, B) and lens emergence (C, D) on the validation (left graph) and test (right graph) data sets (for the data partitioning strategy refer to Fig 3A and Methods). A/C: The data correspond directly to the data shown in S8 Fig but are split for the individual experiments. Raw data of the figure plots have been deposited as Extended Data 46 and 48, respectively. B/D: Confusion matrices. The data correspond to A and C, respectively. The x-axis denotes the respective imaging time points while the y-axes show the relative percentage of true-positive, true-negative, false-positive and false-negative predictions as indicated. Raw data of the figure plots have been deposited as Extended Data 47 and 49, respectively. Predictions were calculated using the function ‘get_classification_f1_data’ of the module orgAInoid.figures.figure_data_generation (compare source code). (TIF) [file pbio.3003597.s017.tif]

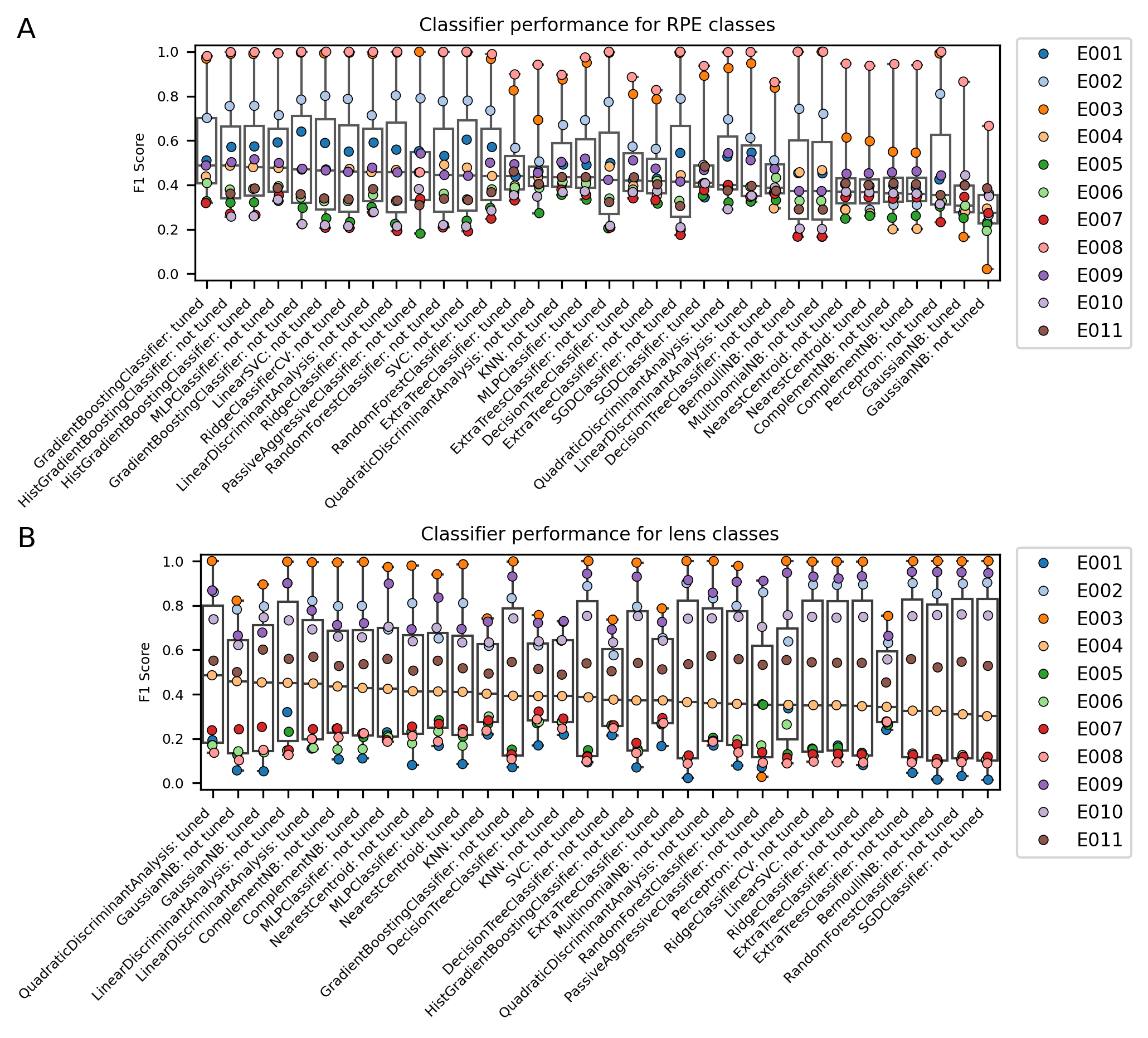

Supplement: S15 Fig — A The indicated classifiers were trained by cross-validation, using the indicated experiment as a test set, and scored using the F1 metric (y-axis) for the prediction of RPE area class. Selected classifiers were subjected to hyperparameter tuning first (tuned). Raw data of the figure plots have been deposited as Extended Data 50. B The indicated classifiers were trained and evaluated as in A, but for the class of lens area. Raw data of the figure plots have been deposited as Extended Data 51. (TIF) [file pbio.3003597.s018.tif]

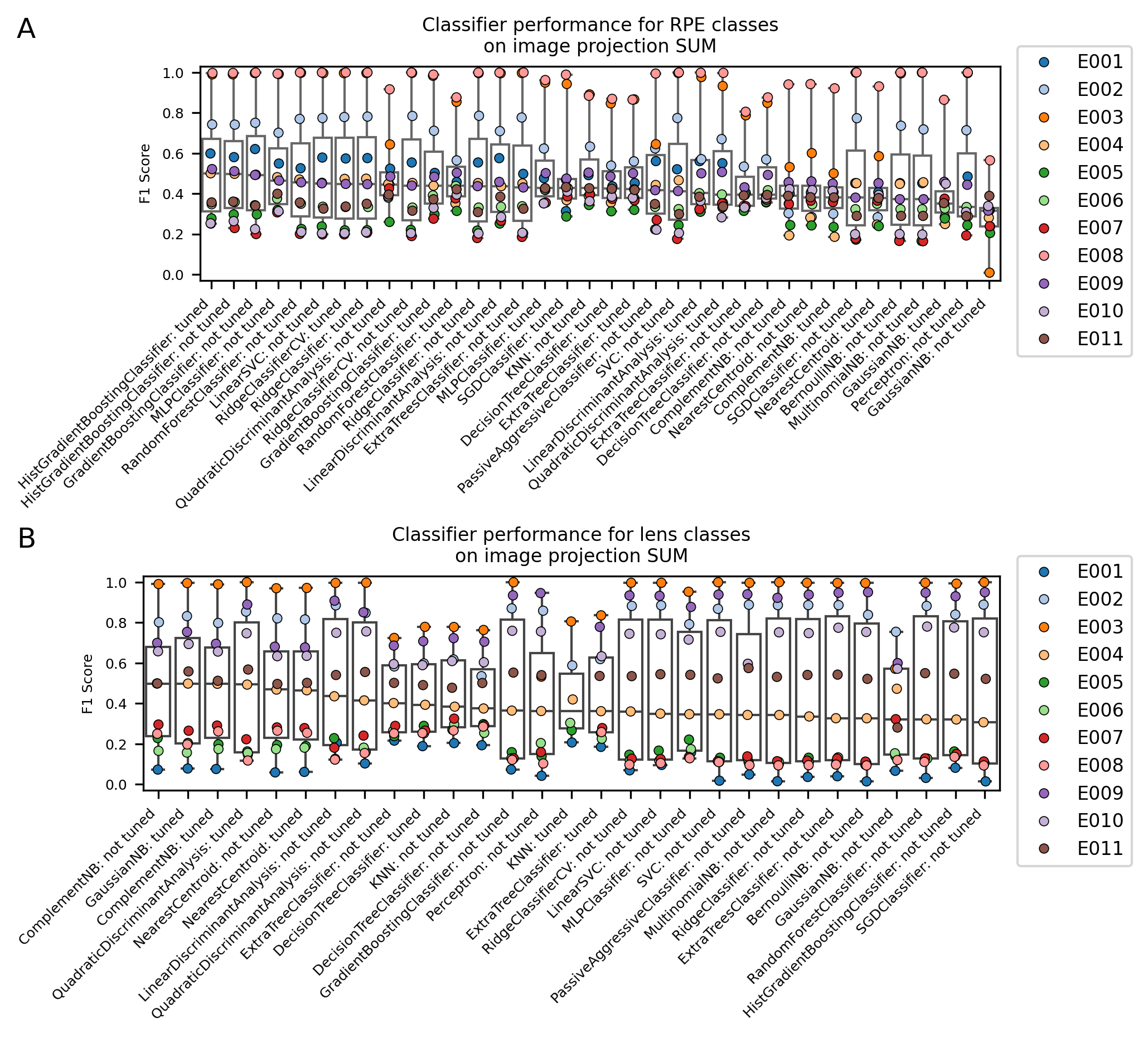

Supplement: S16 Fig — A The indicated classifiers were trained by cross-validation, using the indicated experiment as a test set, and scored using the F1 metric (y-axis) for the prediction of RPE area class. Selected classifiers were subjected to hyperparameter tuning first (tuned). Raw data of the figure plots have been deposited as Extended Data 52. B The indicated classifiers were trained and evaluated as in A, but for the class of lens area. Raw data of the figure plots have been deposited as Extended Data 53. (TIF) [file pbio.3003597.s019.tif]

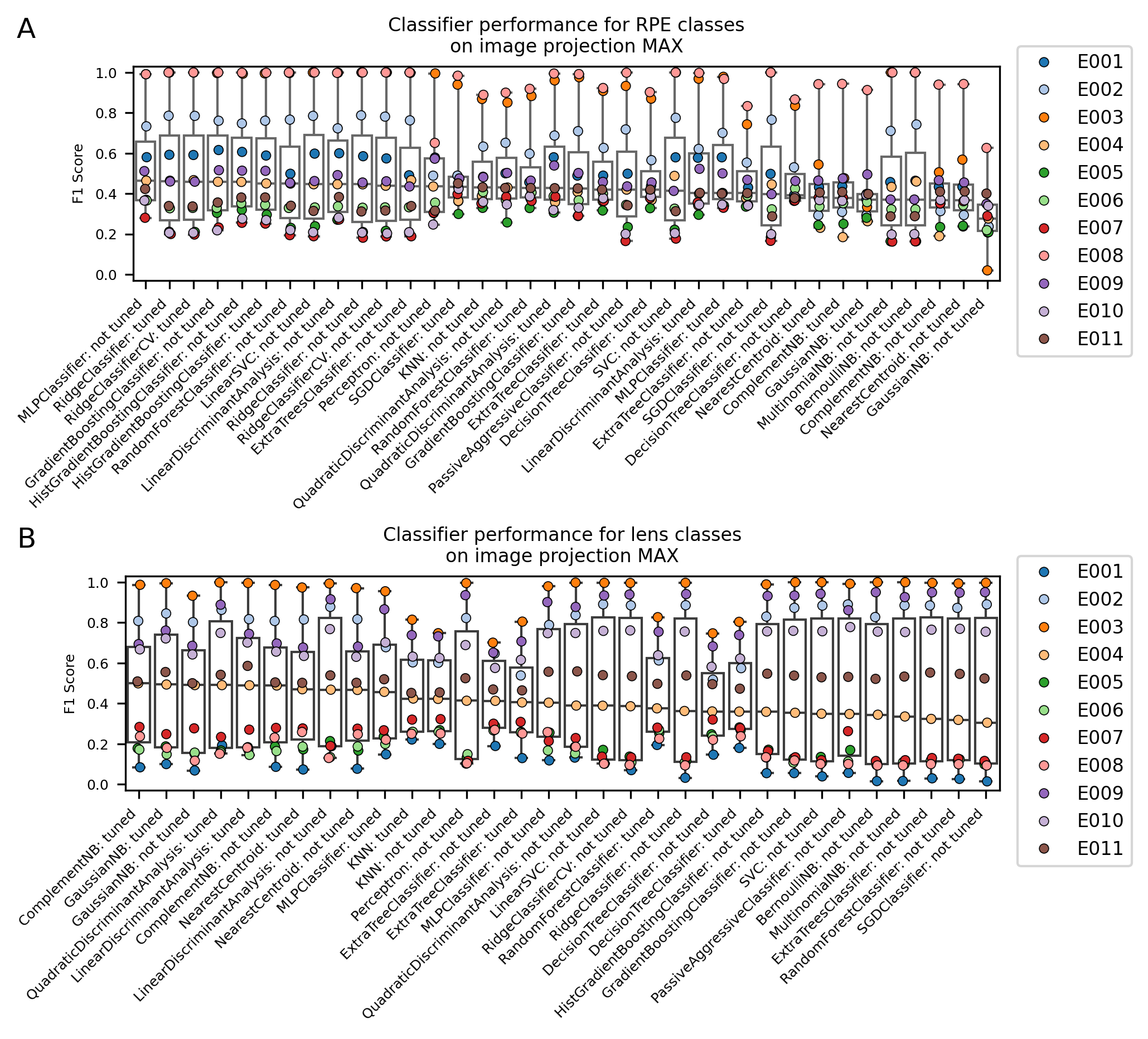

Supplement: S17 Fig — A The indicated classifiers were trained by cross-validation, using the indicated experiment as a test set, and scored using the F1 metric (y-axis) for the prediction of RPE area class. Selected classifiers were subjected to hyperparameter tuning first (tuned). Raw data of the figure plots have been deposited as Extended Data 54. B The indicated classifiers were trained and evaluated as in A, but for the class of lens area. Raw data of the figure plots have been deposited as Extended Data 55. (TIF) [file pbio.3003597.s020.tif]

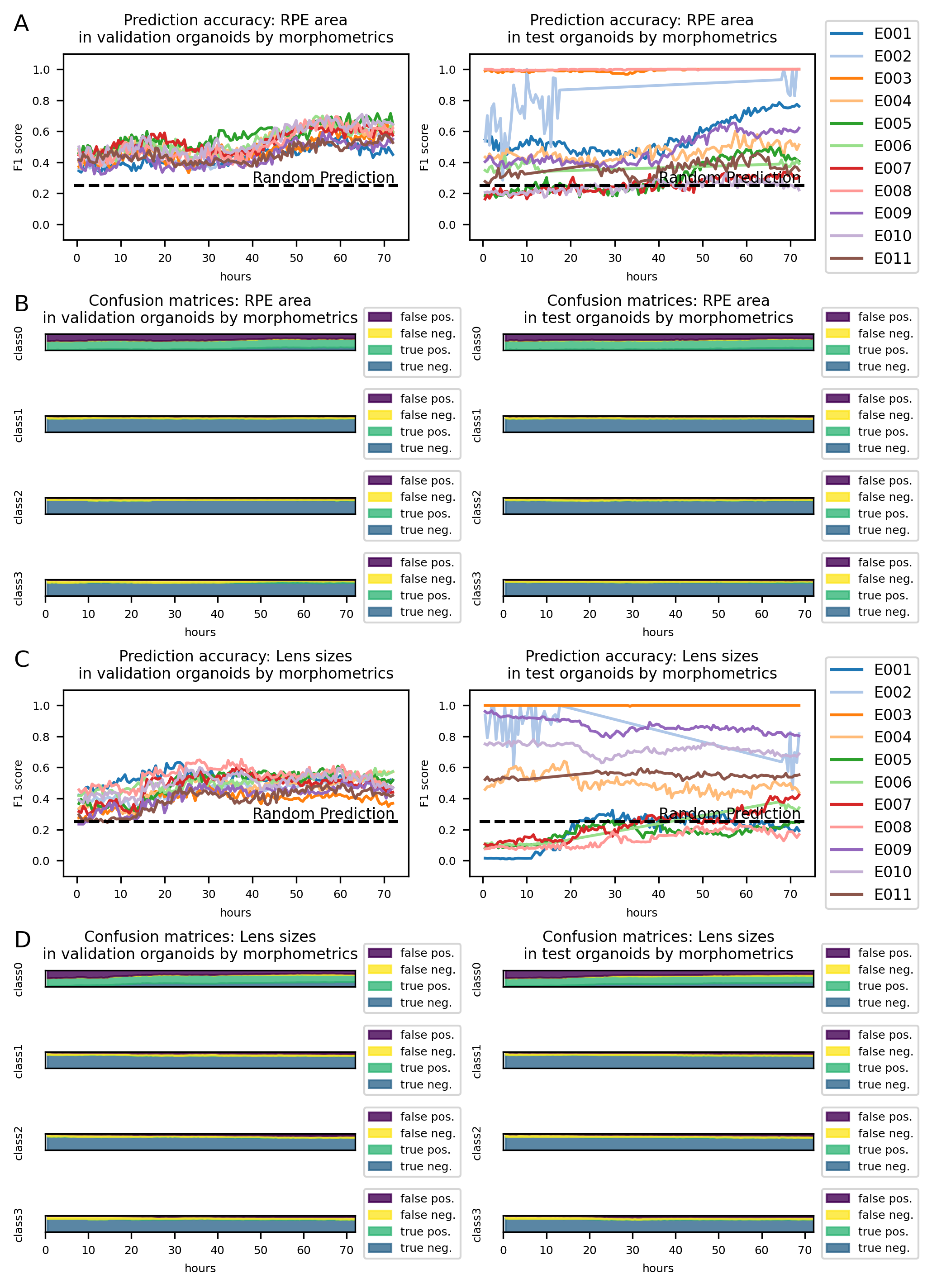

Supplement: S18 Fig — Machine learning classifiers were evaluated on the ability to predict RPE areas (A, B) and lens areas (C, D) on the validation (left graph) and test (right graph) data sets (for the data partitioning strategy refer to Fig 3A and Methods). A/C: The data correspond directly to the data shown in Fig 4A (RPE emergence) and Fig 4B (lens emergence) but are split for the individual experiments. Raw data of the figure plots have been deposited as Extended Data 56 and 58, respectively. B/D: Confusion matrices. The data correspond to A and C, respectively. The x-axis denotes the respective imaging time points while the y-axes show the relative percentage of true-positive, true-negative, false-positive and false-negative predictions as indicated. Raw data of the figure plots have been deposited as Extended Data 57 and 59, respectively. Predictions were calculated using the function ‘get_classification_f1_data’ of the module orgAInoid.figures.figure_data_generation (compare source code). (TIF) [file pbio.3003597.s021.tif]

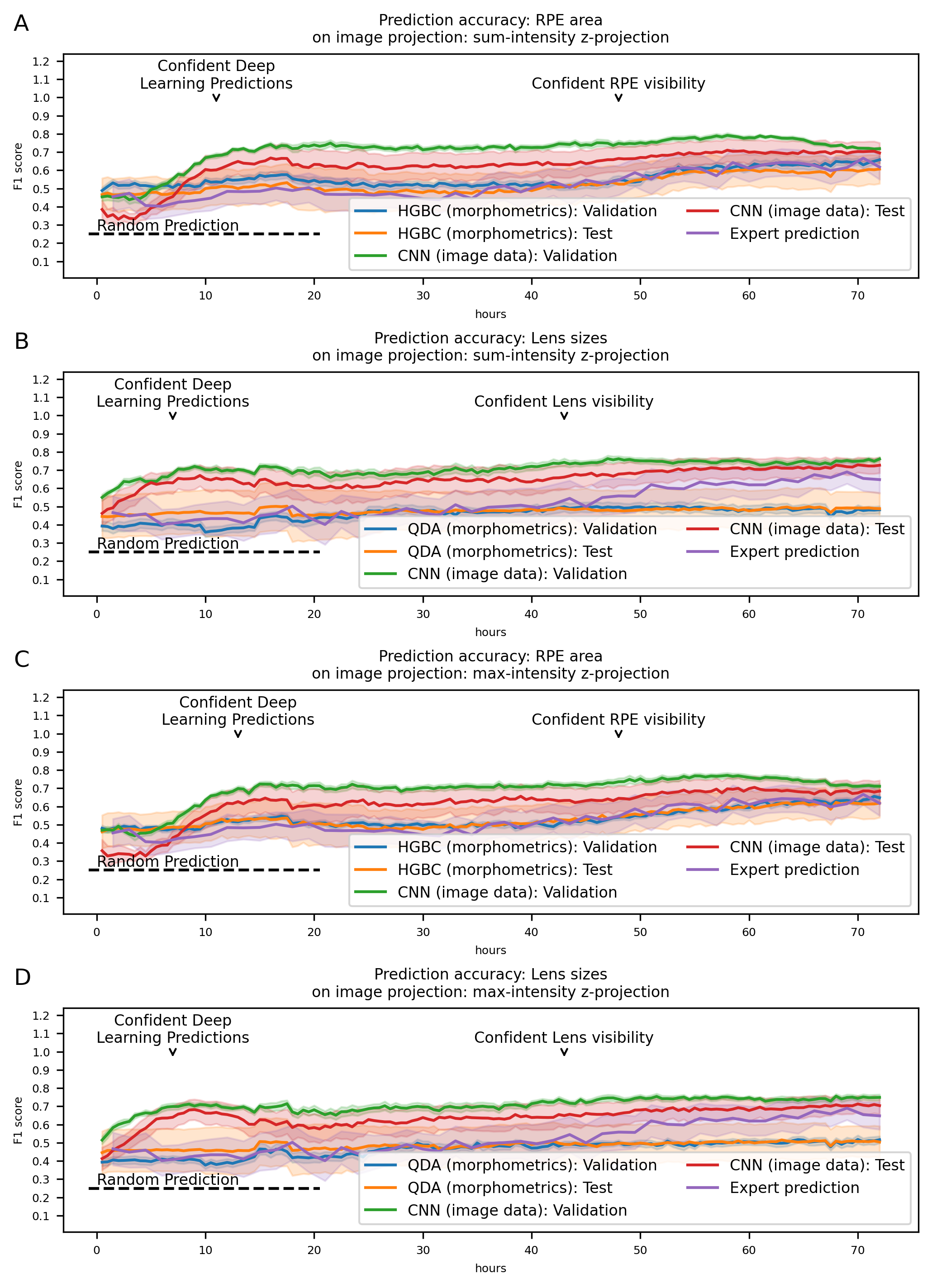

Supplement: S19 Fig — Prediction of RPE (A/C) and lens (B/D) tissue sizes by deep learning well before visibility on images derived from sum- (A/B) or maximum-intensity z-projections (C/D). Sum- and maximum-intensity z-projection of the images did not gain significant performance enhancements compared to single-slice analysis (compare Fig 4). QDA: Quadratic Discriminant Analysis; HGBC: Histogram Gradient Boosting Classifier. Raw data of the figure plots have been deposited as Extended Data 60–63(A–D). Predictions were calculated using the function ‘get_classification_f1_data’ of the module orgAInoid.figures.figure_data_generation (compare source code). (TIF) [file pbio.3003597.s022.tif]

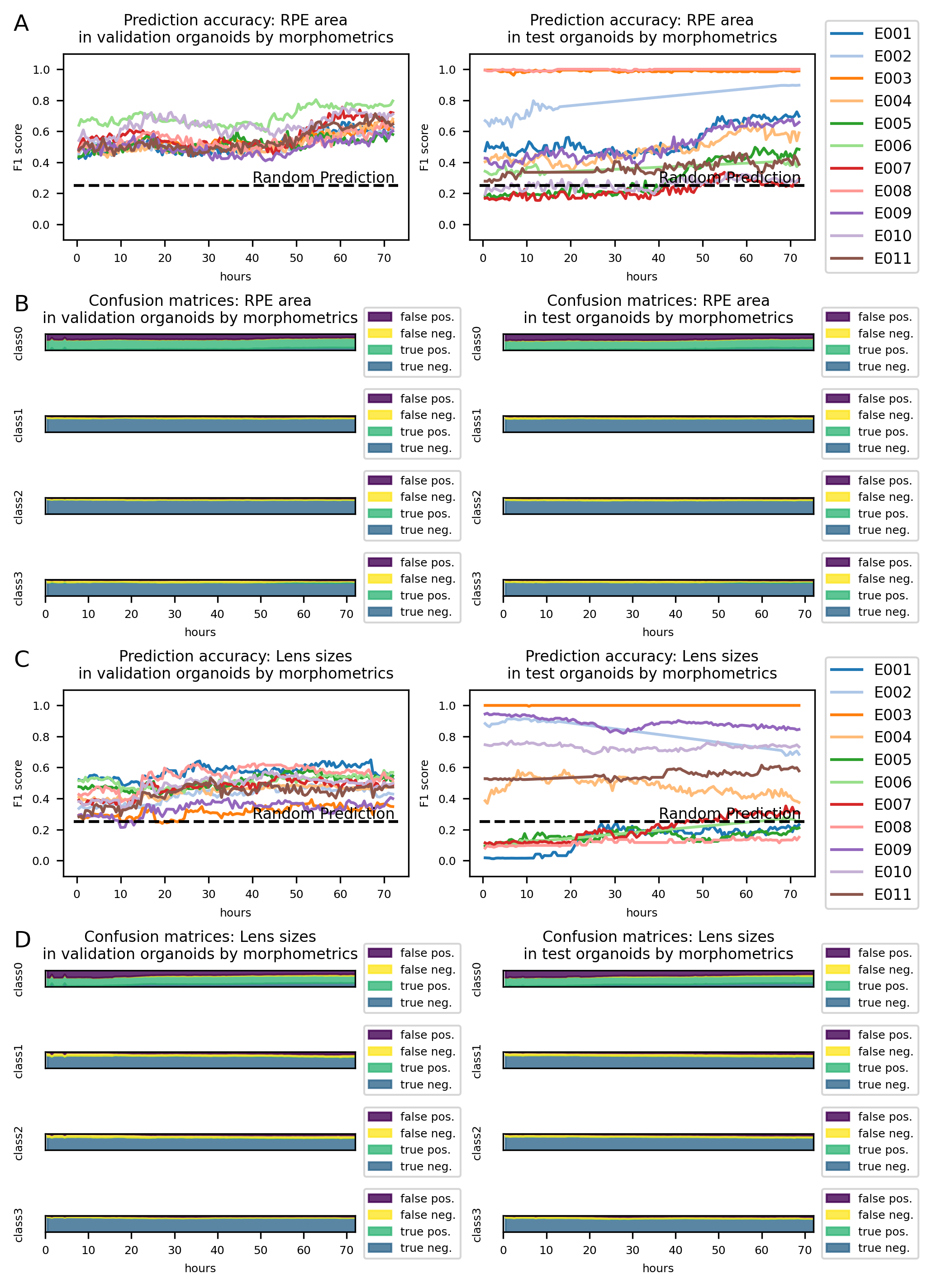

Supplement: S20 Fig — Machine learning classifiers were evaluated on the ability to predict RPE areas (A, B) and lens areas (C, D) on the validation (left graph) and test (right graph) data sets (for the data partitioning strategy refer to Fig 3A and Methods). A/C: The data correspond directly to the data shown in S19 Fig but are split for the individual experiments. Raw data of the figure plots have been deposited as Extended Data 64 and 66, respectively. B/D: Confusion matrices. The data correspond to A and C, respectively. The x-axis denotes the respective imaging time points while the y-axes show the relative percentage of true-positive, true-negative, false-positive and false-negative predictions as indicated. Raw data of the figure plots have been deposited as Extended Data 65 and 67, respectively. Predictions were calculated using the function ‘get_classification_f1_data’ of the module orgAInoid.figures.figure_data_generation (compare source code). (TIF) [file pbio.3003597.s023.tif]

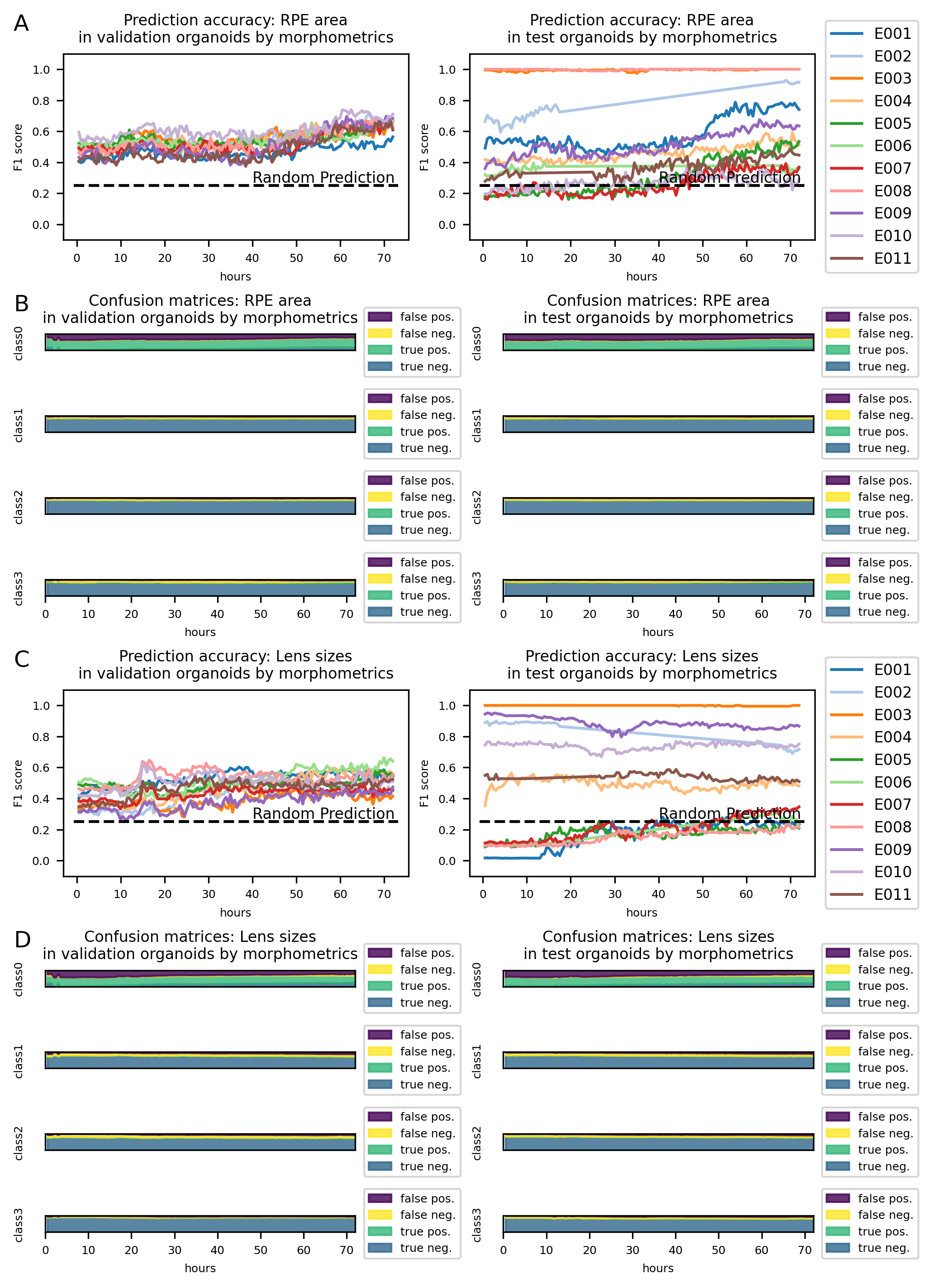

Supplement: S21 Fig — Machine learning classifiers were evaluated on the ability to predict RPE areas (A, B) and lens areas (C, D) on the validation (left graph) and test (right graph) data sets (for the data partitioning strategy refer to Fig 3A and Methods). A/C: The data correspond directly to the data shown in S19 Fig but are split for the individual experiments. Raw data of the figure plots have been deposited as Extended Data 68 and 70, respectively. B/D: Confusion matrices. The data correspond to A and C, respectively. The x-axis denotes the respective imaging time points while the y-axes show the relative percentage of true-positive, true-negative, false-positive and false-negative predictions as indicated. Raw data of the figure plots have been deposited as Extended Data 69 and 71, respectively. Predictions were calculated using the function ‘get_classification_f1_data’ of the module orgAInoid.figures.figure_data_generation (compare source code). (TIF) [file pbio.3003597.s024.tif]

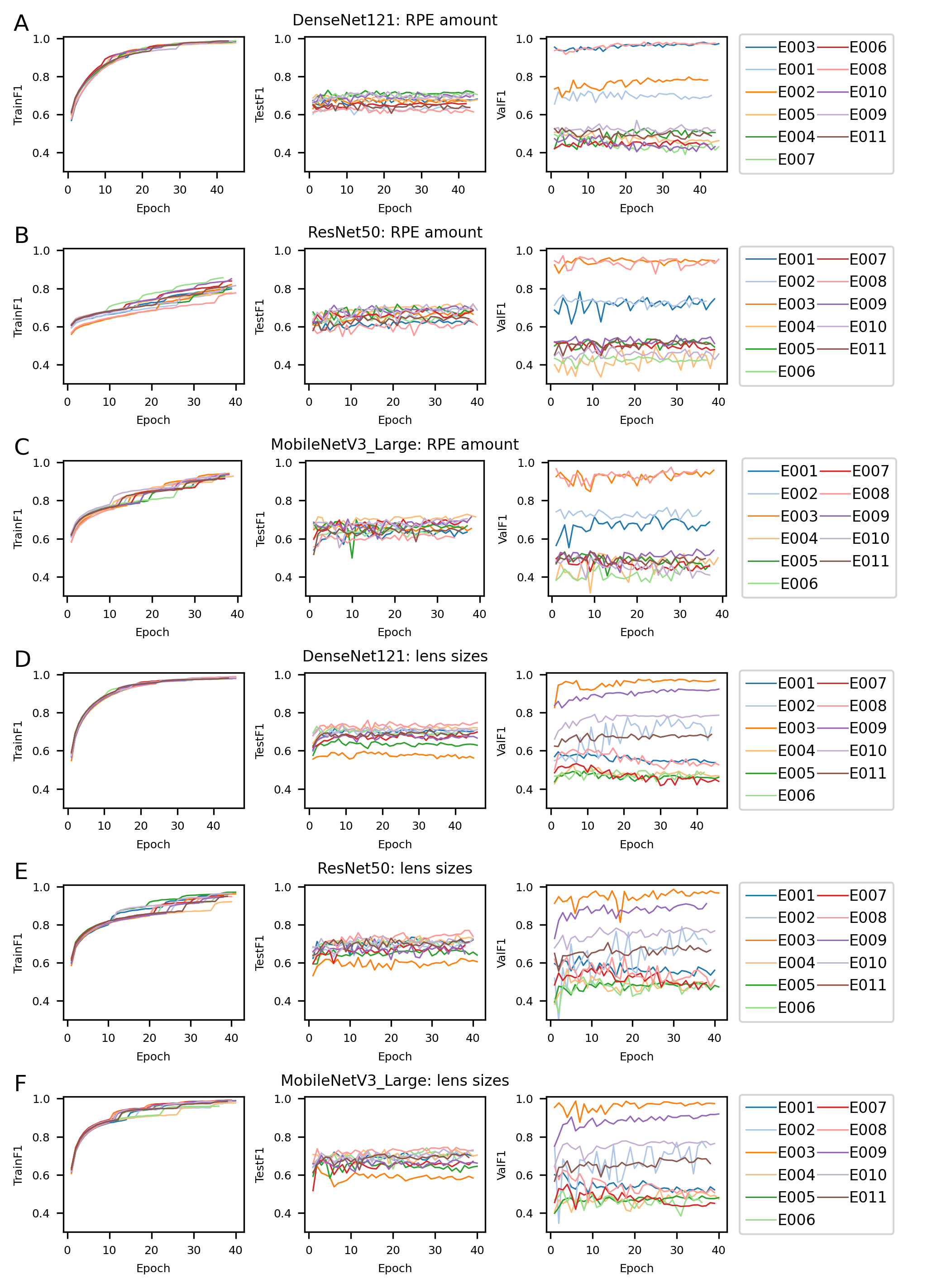

Supplement: S22 Fig — CNNs were trained to predict the areas of RPE (A–C) and lenses (D–F) from time-lapse images for the indicated amount of epochs and scored for the F1 metric in the training set (left graph), the validation set (middle graph) and the test set (right graph). The architecture of the respective CNN is noted within the title. Raw data of the figure plots have been deposited as Extended Data 72. (TIF) [file pbio.3003597.s025.tif]

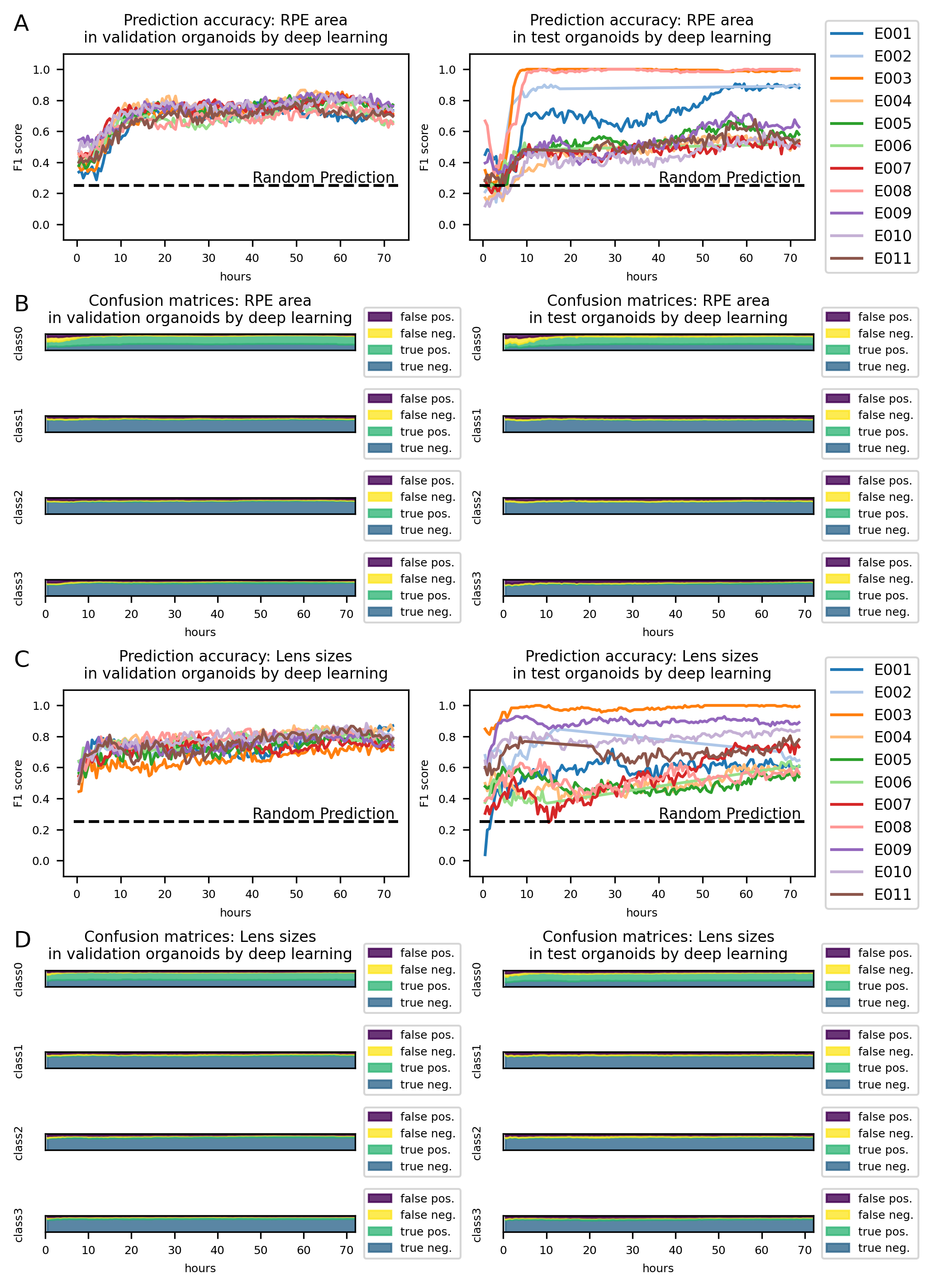

Supplement: S23 Fig — Deep learning classifiers were evaluated on the ability to predict RPE areas (A, B) and lens areas (C, D) on the validation (left graph) and test (right graph) data sets (for the data partitioning strategy refer to Fig 3A and Methods). A/C: The data correspond directly to the data shown in Fig 4A (RPE emergence) and Fig 4B (lens emergence) but are split for the individual experiments. Raw data of the figure plots have been deposited as Extended Data 73 and 75, respectively. B/D: Confusion matrices. The data correspond to A and C, respectively. The x-axis denotes the respective imaging time points while the y-axes show the relative percentage of true-positive, true-negative, false-positive and false-negative predictions as indicated. Raw data of the figure plots have been deposited as Extended Data 74 and 76, respectively. Predictions were calculated using the function ‘get_classification_f1_data’ of the module orgAInoid.figures.figure_data_generation (compare source code). (TIF) [file pbio.3003597.s026.tif]

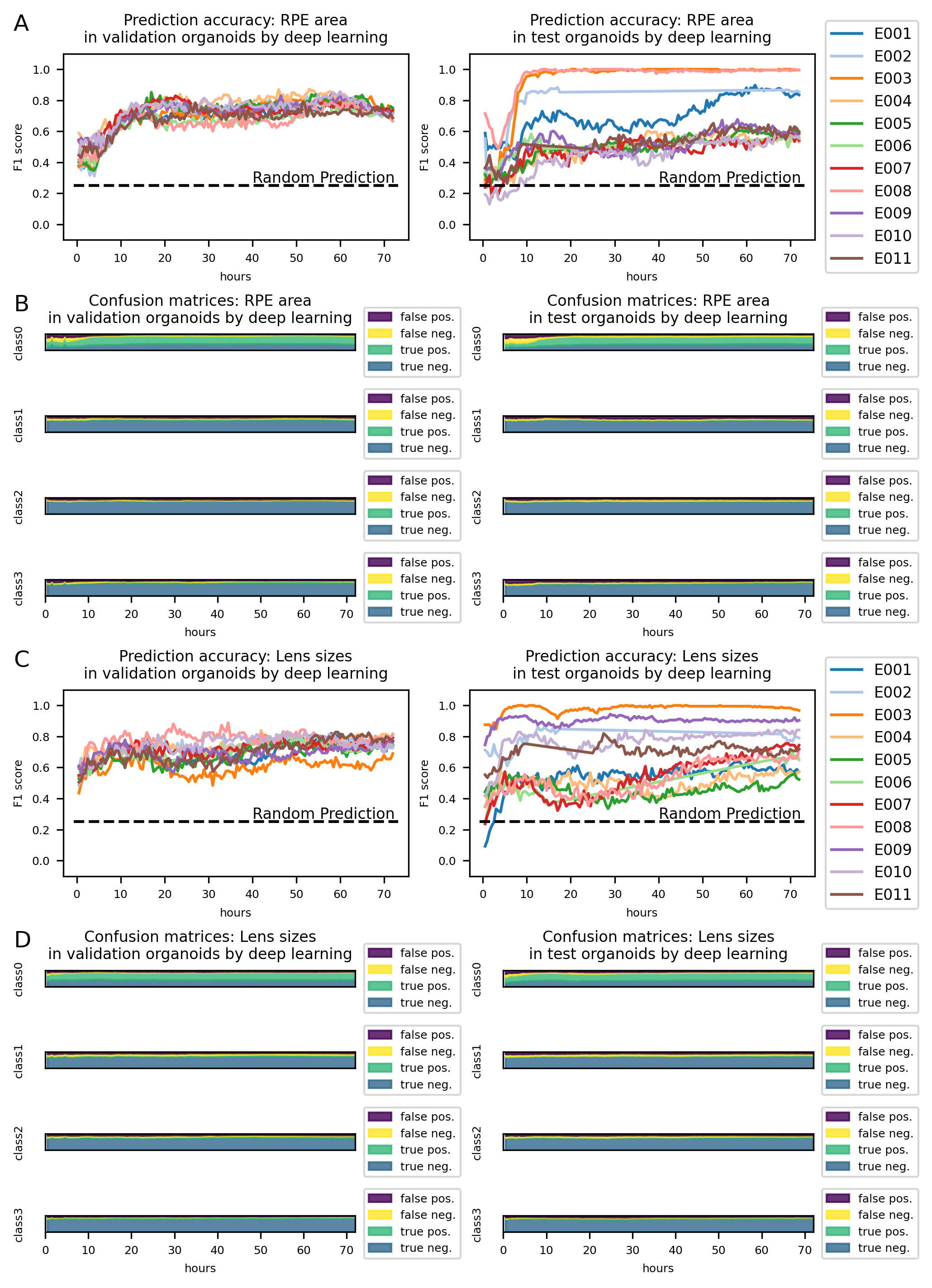

Supplement: S24 Fig — Deep learning classifiers were evaluated on the ability to predict RPE areas (A, B) and lens areas (C, D) on the validation (left graph) and test (right graph) data sets (for the data partitioning strategy refer to Fig 3A and Methods). A/C: The data correspond directly to the data shown in S19 Fig but are split for the individual experiments. Raw data of the figure plots have been deposited as Extended Data 77 and 79, respectively. B/D: Confusion matrices. The data correspond to A and C, respectively. The x-axis denotes the respective imaging time points while the y-axes show the relative percentage of true-positive, true-negative, false-positive and false-negative predictions as indicated. Raw data of the figure plots have been deposited as Extended Data 78 and 80, respectively. Predictions were calculated using the function ‘get_classification_f1_data’ of the module orgAInoid.figures.figure_data_generation (compare source code). (TIF) [file pbio.3003597.s027.tif]

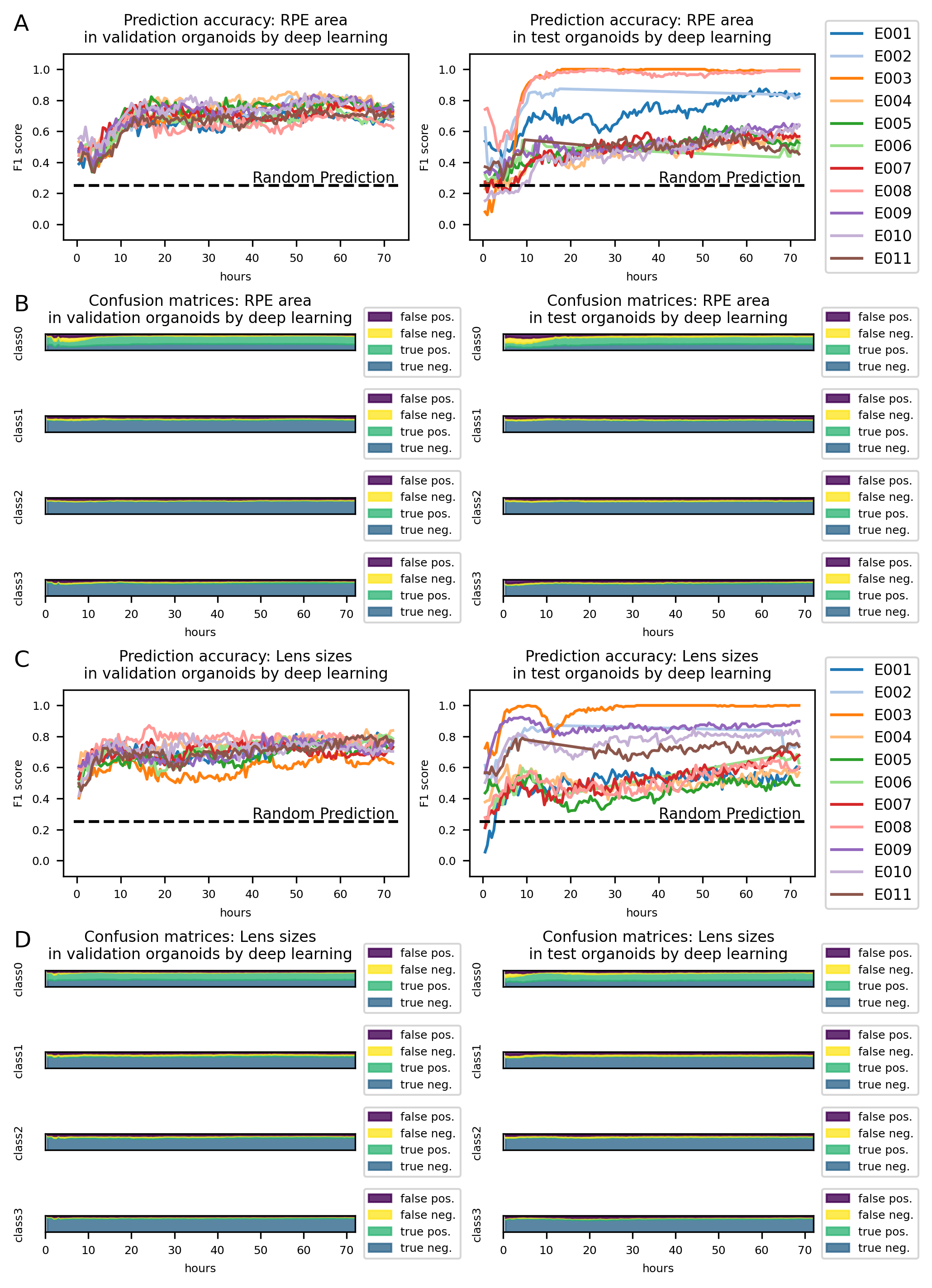

Supplement: S25 Fig — Deep learning classifiers were evaluated on the ability to predict RPE areas (A, B) and lens areas (C, D) on the validation (left graph) and test (right graph) data sets (for the data partitioning strategy refer to Fig 3A and Methods). A/C: The data correspond directly to the data shown in S19 Fig but are split for the individual experiments. Raw data of the figure plots have been deposited as Extended Data 81 and 83, respectively. B/D: Confusion matrices. The data correspond to A and C, respectively. The x-axis denotes the respective imaging time points while the y-axes show the relative percentage of true-positive, true-negative, false-positive and false-negative predictions as indicated. Raw data of the figure plots have been deposited as Extended Data 82 and 84, respectively. Predictions were calculated using the function ‘get_classification_f1_data’ of the module orgAInoid.figures.figure_data_generation (compare source code). (TIF) [file pbio.3003597.s028.tif]

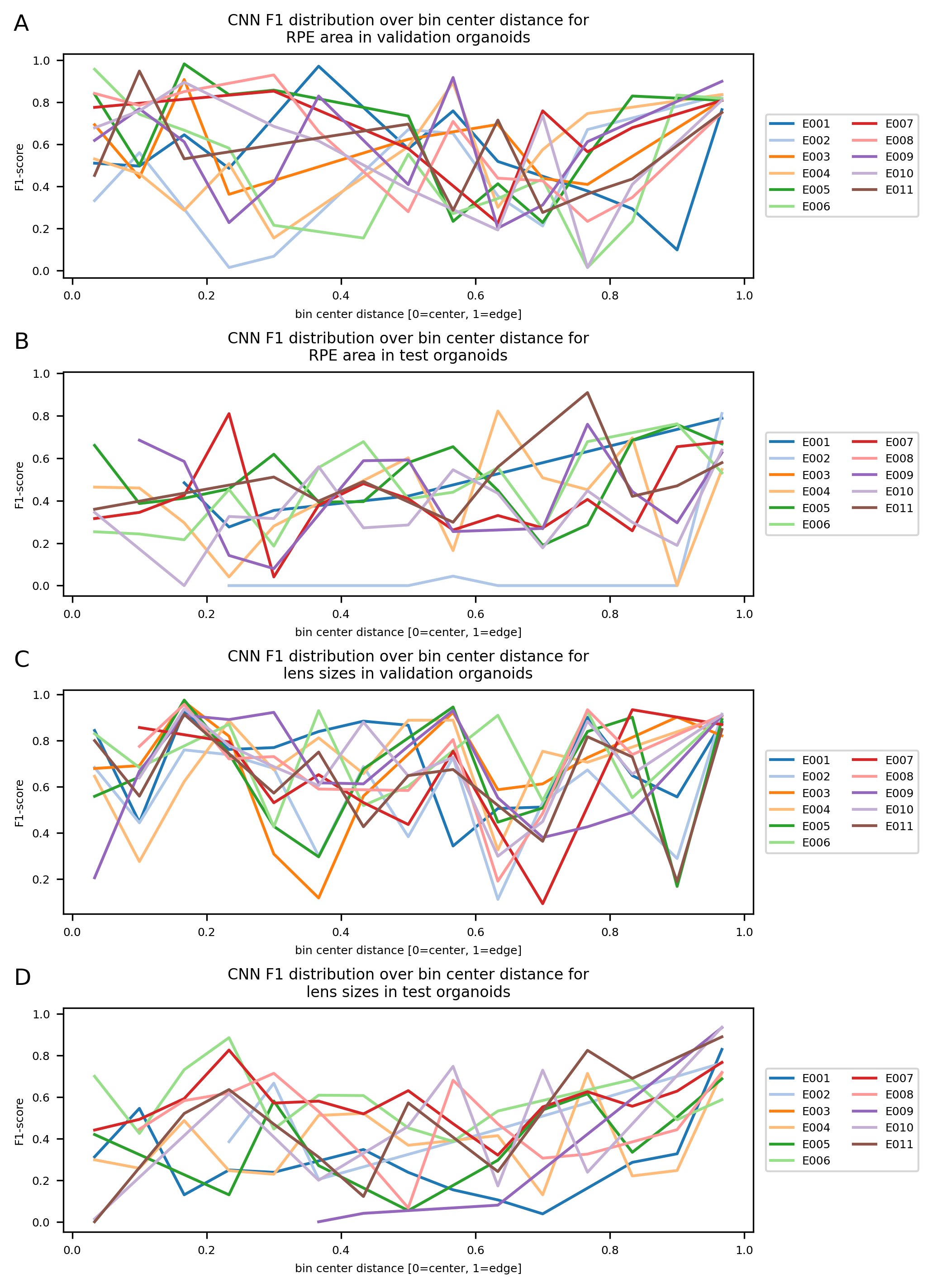

Supplement: S26 Fig — A–D Weighted F1 scores for RPE area (A/B) and lens size (C/D) in validation (A/C) and test (B/D) organoids. CNNs demonstrate a more balanced performance across bin distances, without systematic differences between center- and edge-proximal samples. Raw data of the figure plots have been deposited as Extended Data 85. (TIF) [file pbio.3003597.s029.tif]

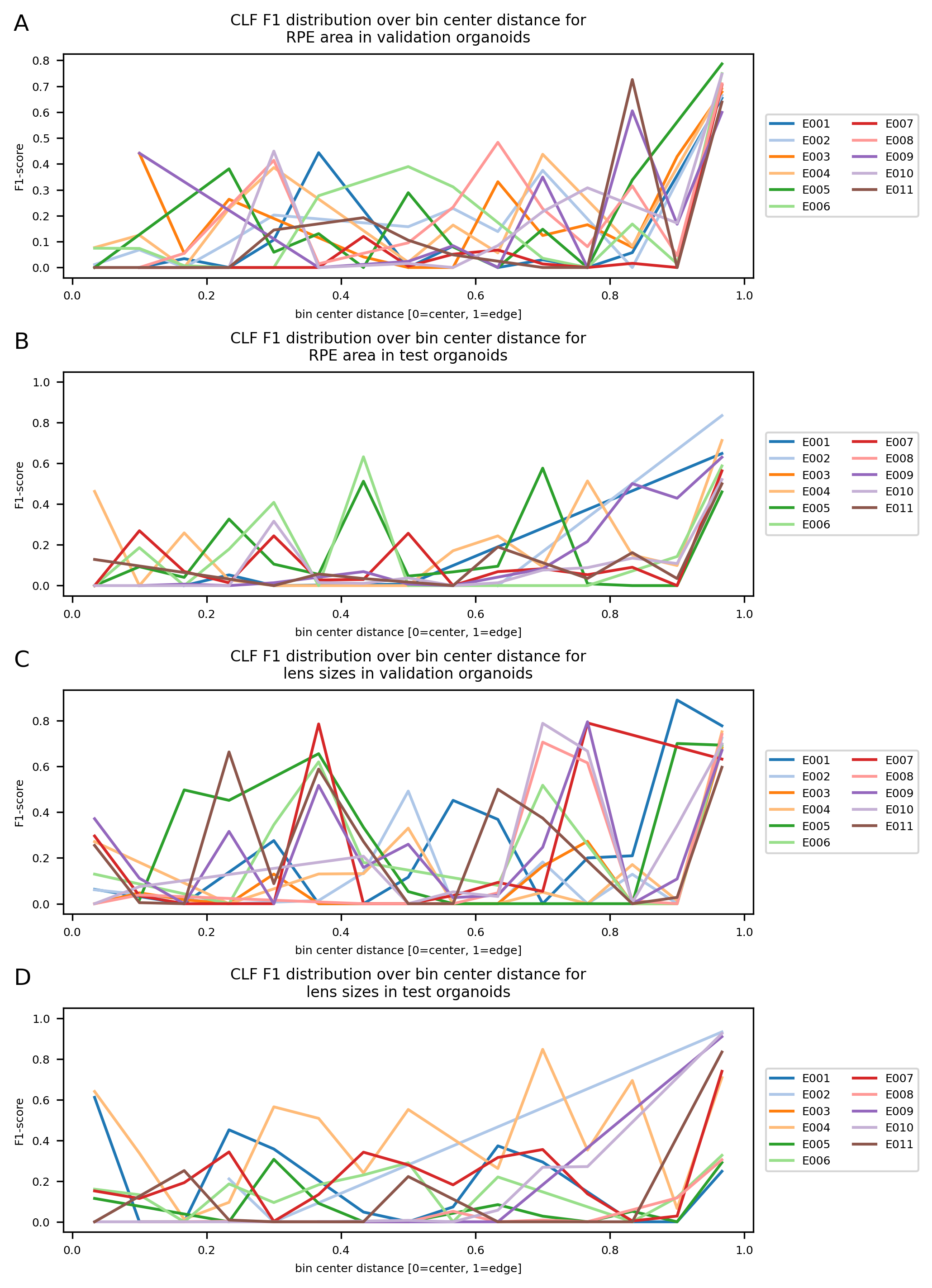

Supplement: S27 Fig — A–D Weighted F1 scores for RPE area (A/B) and lens size (C/D) in validation (A/C) and test (B/D) organoids. The x-axis indicates the normalized distance from the bin center (0 = center, 1 = edge), and the y-axis shows the weighted F1 score. Each line represents one test experiment (E001–E011). Classifiers show a tendency for higher F1 scores near bin edges. Raw data of the figure plots have been deposited as Extended Data 86. (TIF) [file pbio.3003597.s030.tif]

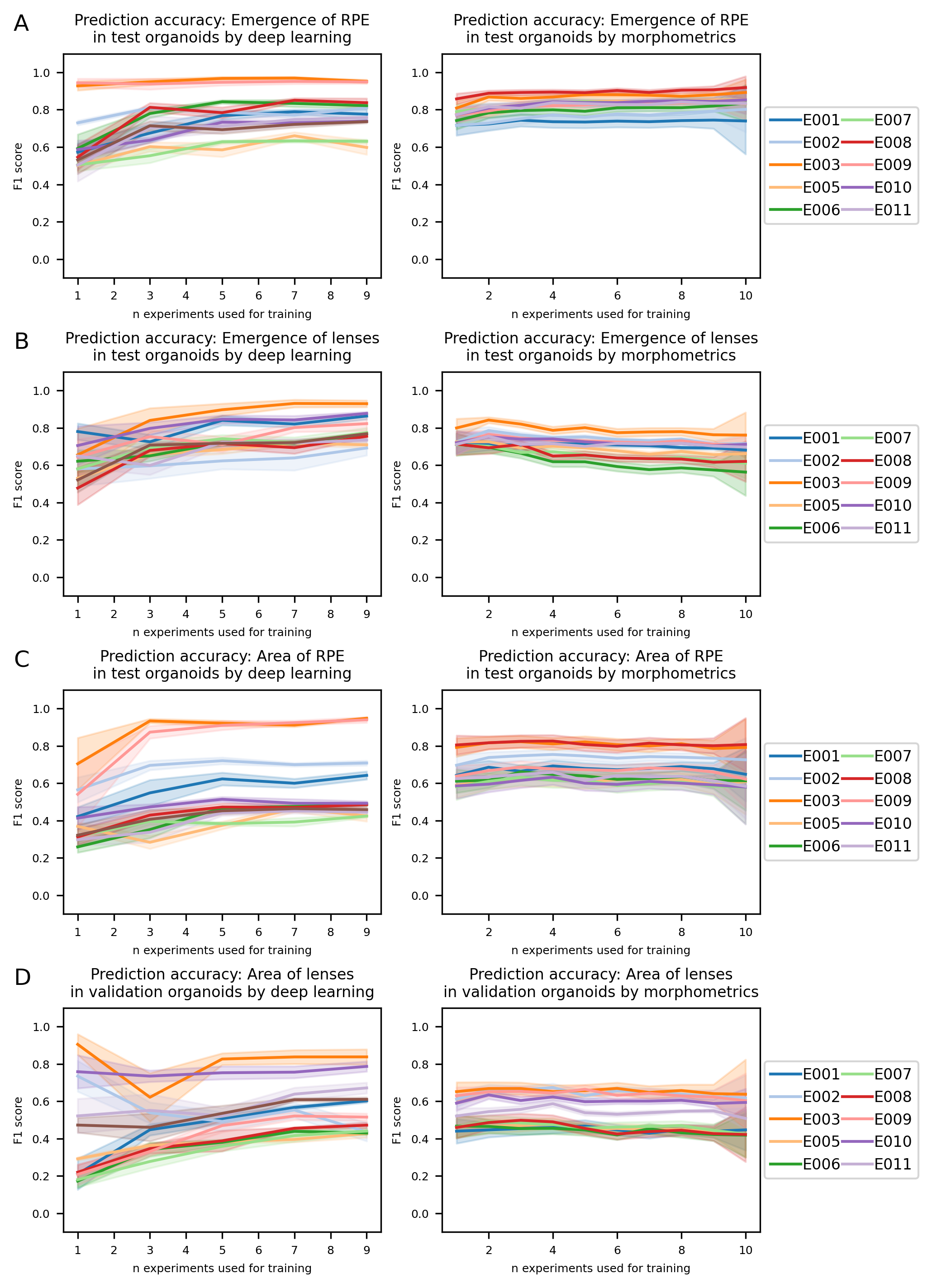

Supplement: S28 Fig — Number of experiments needed for an accurate prediction of RPE (A) and lens (B) emergence, and RPE (C) and lens (D) area. The experiments corresponding to Figs 3 and 4 were repeated with the indicated number of total experiments (x-axis) used for training. We chose MobileNetV3_Large as the CNN due to computational resources, which was trained for exactly one epoch (left graph), as we did not observe a striking increase in validation accuracy after a higher number of epochs (compare S11 and S22 Figs). The machine learning classifiers (right graph) were used similarly to Figs 3 and 4, dependent on the classification task. While the curves plateaued at approximately 6 experiments for the classification by neural networks, we could not observe a similar trend for the machine learning classifiers, indicating that tabular data guided classification needs fewer training experiments for a comparable accuracy on the test set. Raw data of the figure plots have been deposited as Extended Data 87 and 88(deep learning and morphometrics, respectively). (TIF) [file pbio.3003597.s031.tif]

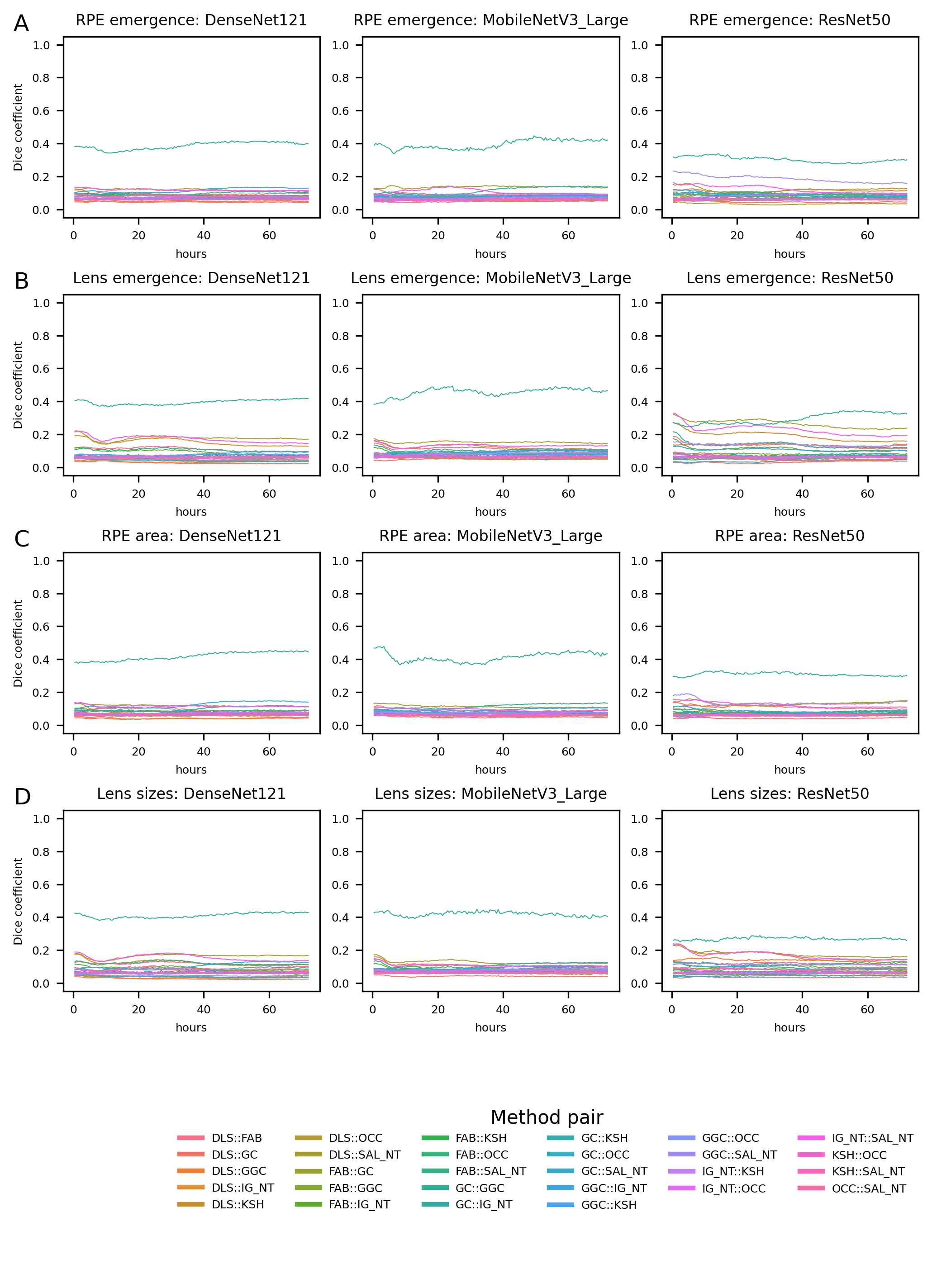

Supplement: S29 Fig — Saliency maps were generated for three CNN architectures (DenseNet121, MobileNetV3_Large, ResNet50) using eight attribution methods: integrated gradients (IG_NT), simple saliency (SAL_NT), DeepLIFT SHAP (DLS), Grad-CAM (GC), guided Grad-CAM (GGC), smooth occlusion (OCC), feature ablation (FAB), and kernel SHAP (KSH). For each method pair within a given model, Dice coefficients were calculated by thresholding the top 1%, 5%, and 10% of saliency values inside the organoid mask and averaging across thresholds. Shown are mean Dice coefficients over time for each readout (A, RPE emergence; B, lens emergence; C, RPE area; D, lens size). Most method pairs displayed low overlap close to baseline levels, while Grad-CAM and guided Grad-CAM consistently achieved higher agreement. Raw data of the figure plots have been deposited as Extended Data 89. (TIF) [file pbio.3003597.s032.tif]

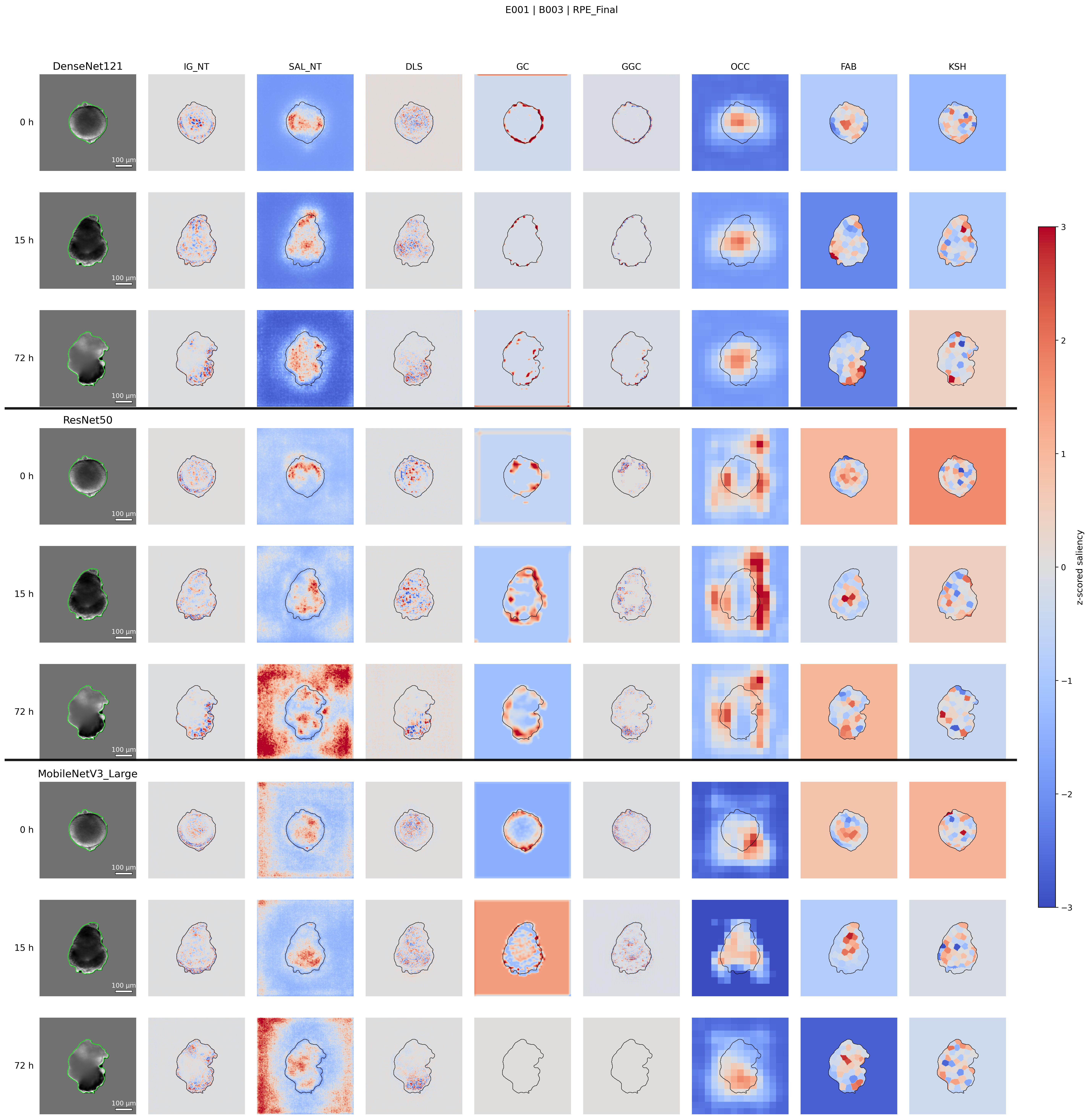

Supplement: S30 Fig — Shown are saliency maps for a representative organoid (experiment E001, well B003) across three convolutional neural network architectures (DenseNet121, ResNet50, MobileNetV3_Large) and three time points (0 h, 15 h, 72 h). Columns display attribution results for eight methods: Integrated Gradients (IG_NT), Saliency (SAL_NT), DeepLiftSHAP (DLS), Grad-CAM (GC), Guided Grad-CAM (GGC), Smooth Occlusion (OCC), Feature Ablation (FAB), and Kernel SHAP (KSH). Input images with organoid masks (green) are shown in the first column of each block. Saliencies were z-scored within the organoid mask, and heatmaps are displayed on a common color scale (blue = low, red = high). The comparison highlights differences in how CNN architectures and attribution methods assign relevance, with CAM-based approaches showing more localized hotspots, perturbation-based methods distributing relevance across discrete patches, and gradient-based methods yielding broader and more diffuse patterns. (TIF) [file pbio.3003597.s033.tif]

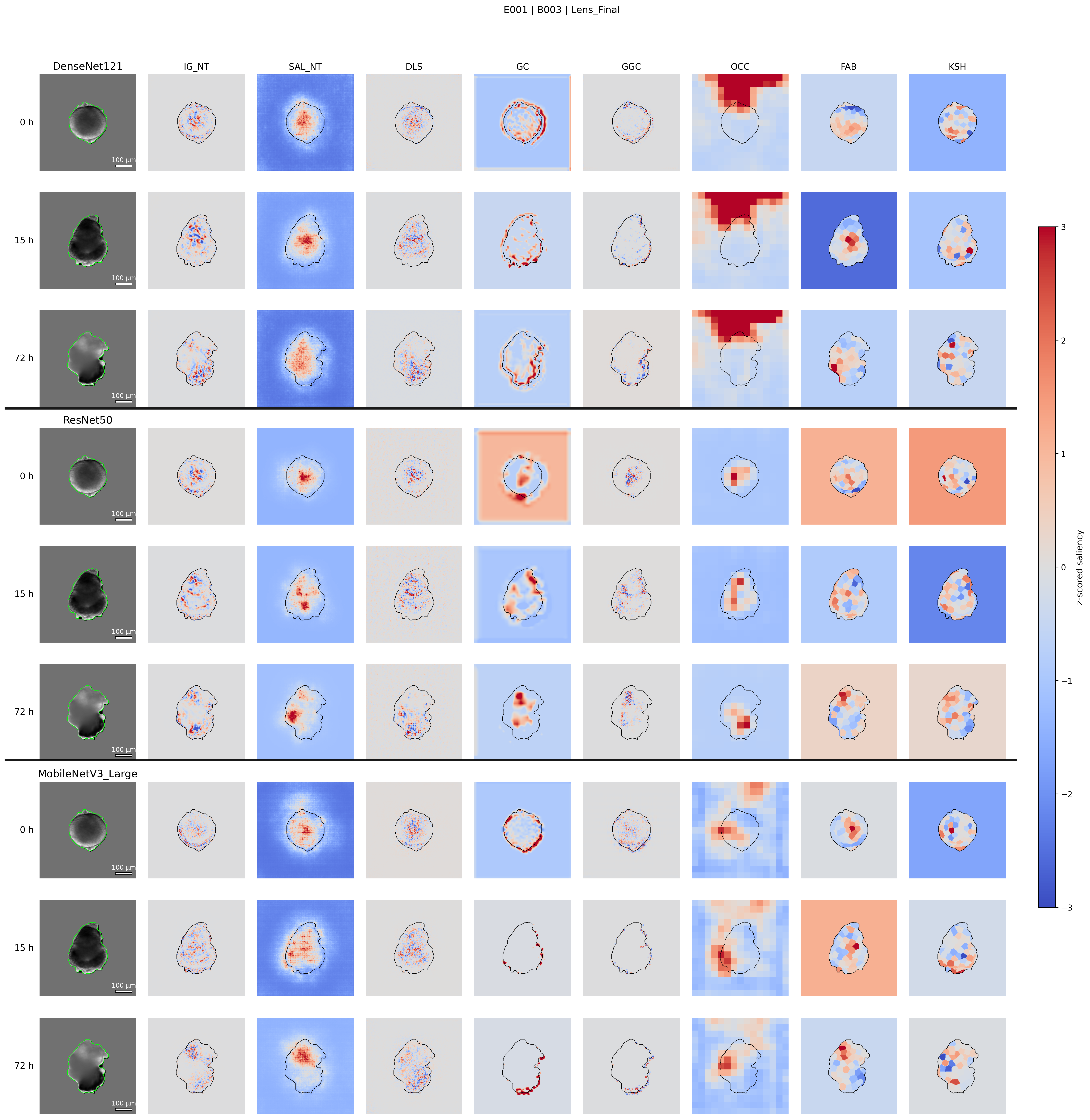

Supplement: S31 Fig — Shown are saliency maps for a representative organoid (experiment E001, well B003) across three convolutional neural network architectures (DenseNet121, ResNet50, MobileNetV3_Large) and three time points (0 h, 15 h, 72 h). Columns display attribution results for eight methods: Integrated Gradients (IG_NT), Saliency (SAL_NT), DeepLiftSHAP (DLS), Grad-CAM (GC), Guided Grad-CAM (GGC), Smooth Occlusion (OCC), Feature Ablation (FAB), and Kernel SHAP (KSH). Input images with organoid masks (green) are shown in the first column of each block. Saliencies were z-scored within the organoid mask, and heatmaps are displayed on a common color scale (blue = low, red = high). The comparison highlights differences in how CNN architectures and attribution methods assign relevance, with CAM-based approaches showing more localized hotspots, perturbation-based methods distributing relevance across discrete patches, and gradient-based methods yielding broader and more diffuse patterns. (TIF) [file pbio.3003597.s034.tif]

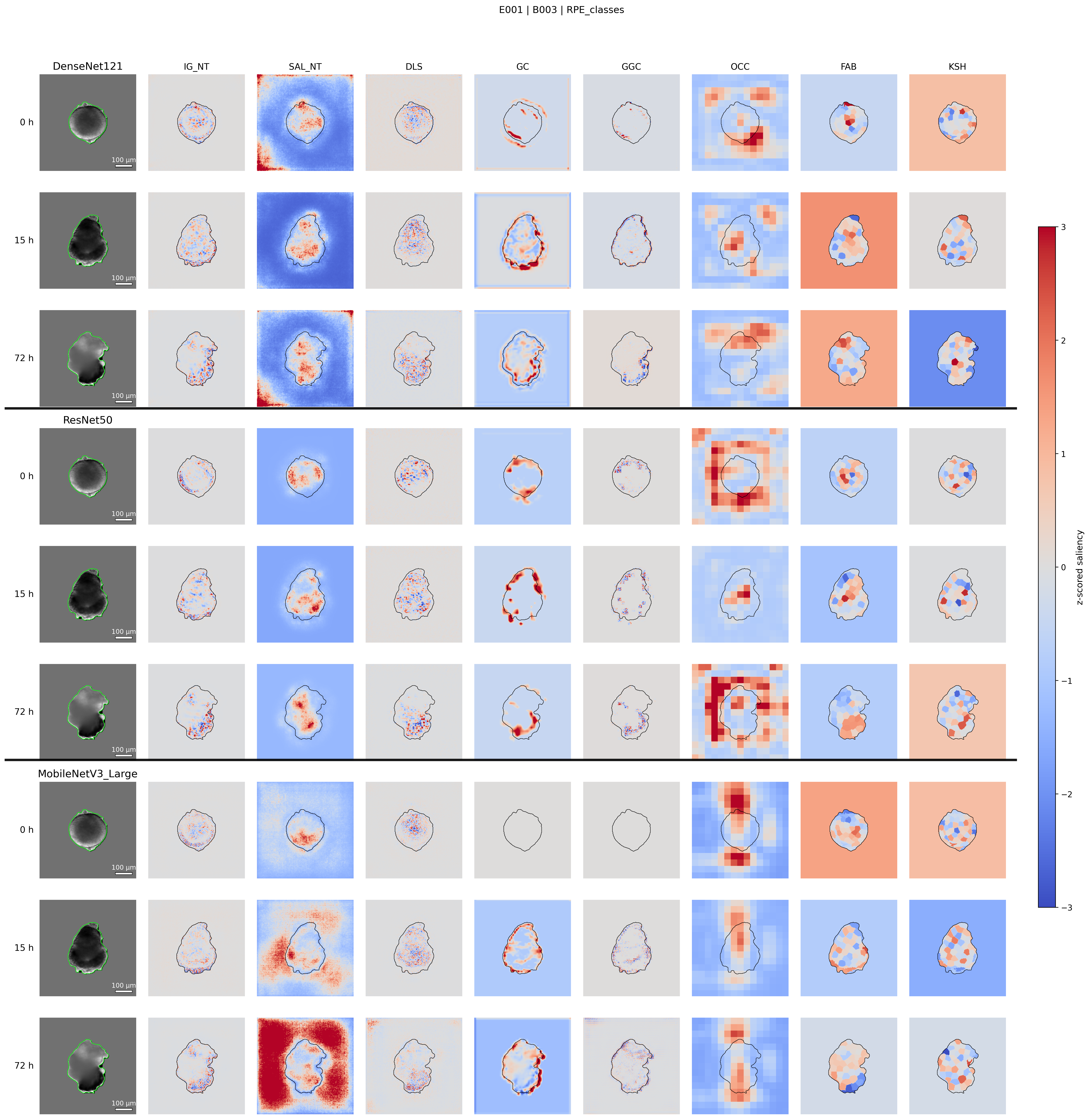

Supplement: S32 Fig — Shown are saliency maps for a representative organoid (experiment E001, well B003) across three convolutional neural network architectures (DenseNet121, ResNet50, MobileNetV3_Large) and three time points (0 h, 15 h, 72 h). Columns display attribution results for eight methods: Integrated Gradients (IG_NT), Saliency (SAL_NT), DeepLiftSHAP (DLS), Grad-CAM (GC), Guided Grad-CAM (GGC), Smooth Occlusion (OCC), Feature Ablation (FAB), and Kernel SHAP (KSH). Input images with organoid masks (green) are shown in the first column of each block. Saliencies were z-scored within the organoid mask, and heatmaps are displayed on a common color scale (blue = low, red = high). The comparison highlights differences in how CNN architectures and attribution methods assign relevance, with CAM-based approaches showing more localized hotspots, perturbation-based methods distributing relevance across discrete patches, and gradient-based methods yielding broader and more diffuse patterns. (TIF) [file pbio.3003597.s035.tif]

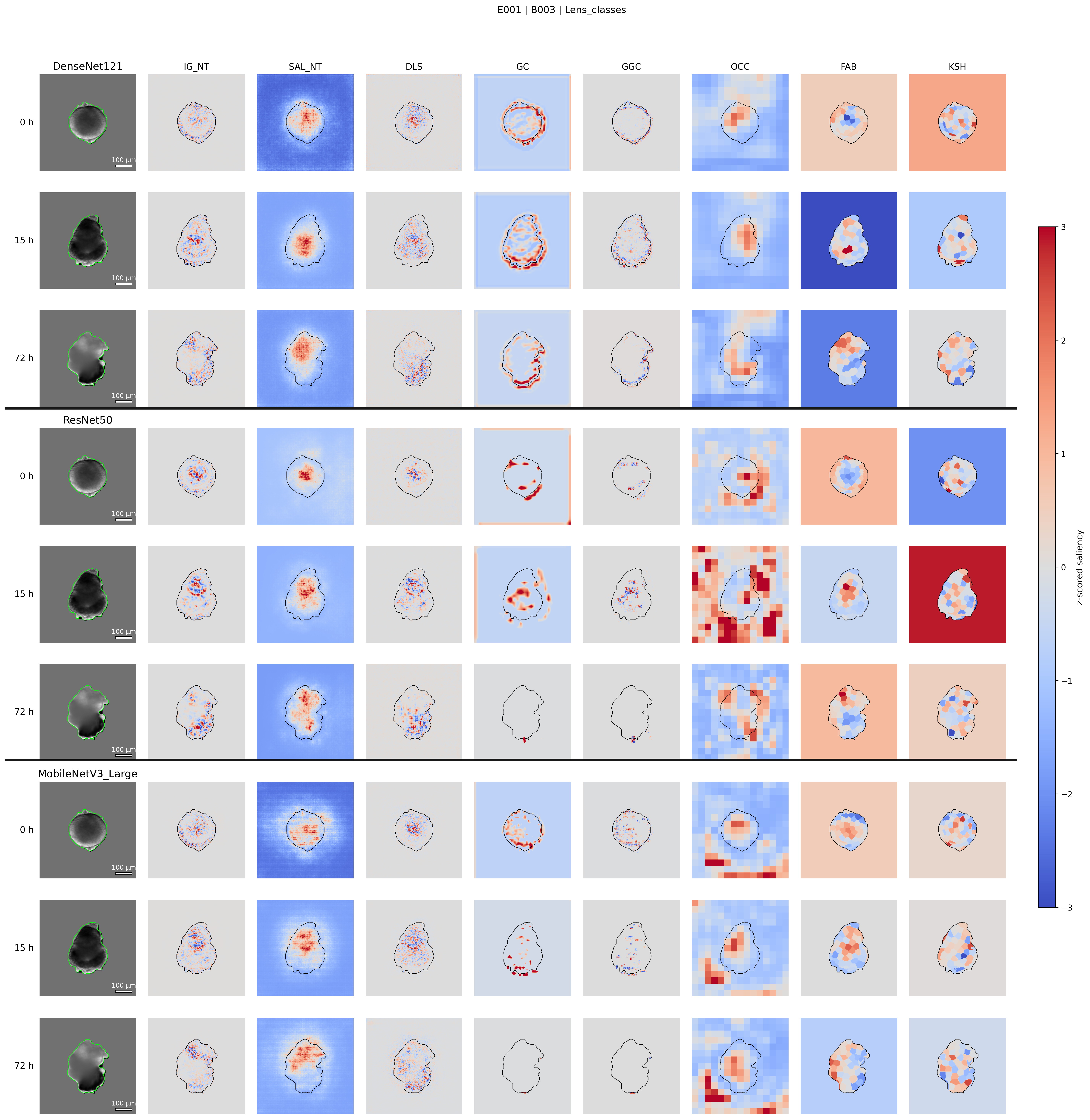

Supplement: S33 Fig — Shown are saliency maps for a representative organoid (experiment E001, well B003) across three convolutional neural network architectures (DenseNet121, ResNet50, MobileNetV3_Large) and three time points (0 h, 15 h, 72 h). Columns display attribution results for eight methods: Integrated Gradients (IG_NT), Saliency (SAL_NT), DeepLiftSHAP (DLS), Grad-CAM (GC), Guided Grad-CAM (GGC), Smooth Occlusion (OCC), Feature Ablation (FAB), and Kernel SHAP (KSH). Input images with organoid masks (green) are shown in the first column of each block. Saliencies were z-scored within the organoid mask, and heatmaps are displayed on a common color scale (blue = low, red = high). The comparison highlights differences in how CNN architectures and attribution methods assign relevance, with CAM-based approaches showing more localized hotspots, perturbation-based methods distributing relevance across discrete patches, and gradient-based methods yielding broader and more diffuse patterns. (TIF) [file pbio.3003597.s036.tif]

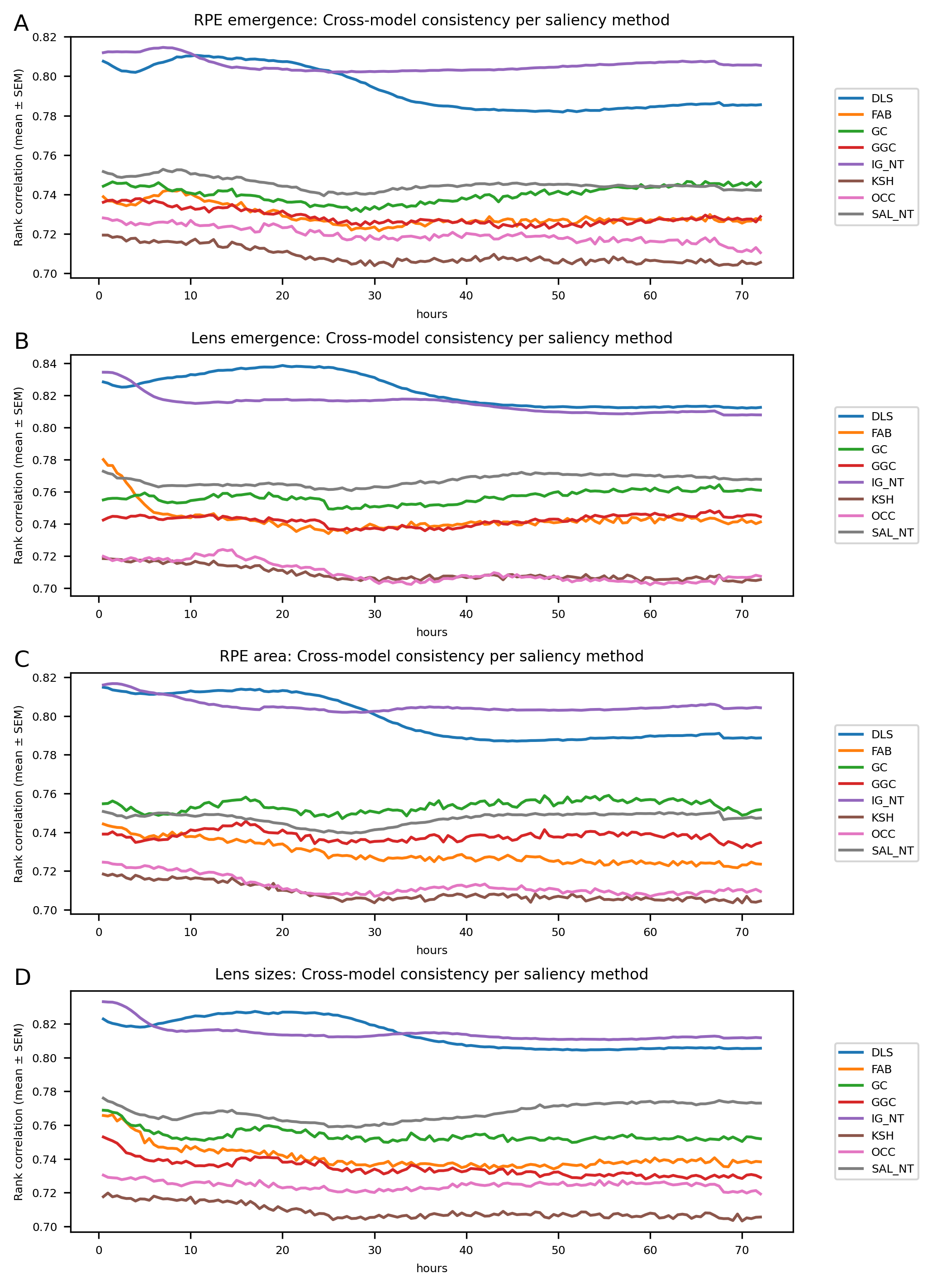

Supplement: S34 Fig — Saliency maps were generated for three CNN architectures (DenseNet121, MobileNetV3_Large, ResNet50) using eight attribution methods: integrated gradients (IG_NT), simple saliency (SAL_NT), DeepLIFT SHAP (DLS), Grad-CAM (GC), guided Grad-CAM (GGC), smooth occlusion (OCC), feature ablation (FAB), and kernel SHAP (KSH). For each method, saliency maps were converted to ranked pixel values (absolute saliency inside the organoid mask) and compared across models using a Spearman-like correlation. Shown are mean correlations ± SEM over time for each readout (A, RPE emergence; B, lens emergence; C, RPE area; D, lens size). Gradient-based approaches such as DeepLIFT SHAP and integrated gradients achieved the highest and most stable consistency across models, while CAM- and perturbation-based methods were less consistent. Raw data of the figure plots have been deposited as Extended Data 90. (TIF) [file pbio.3003597.s037.tif]

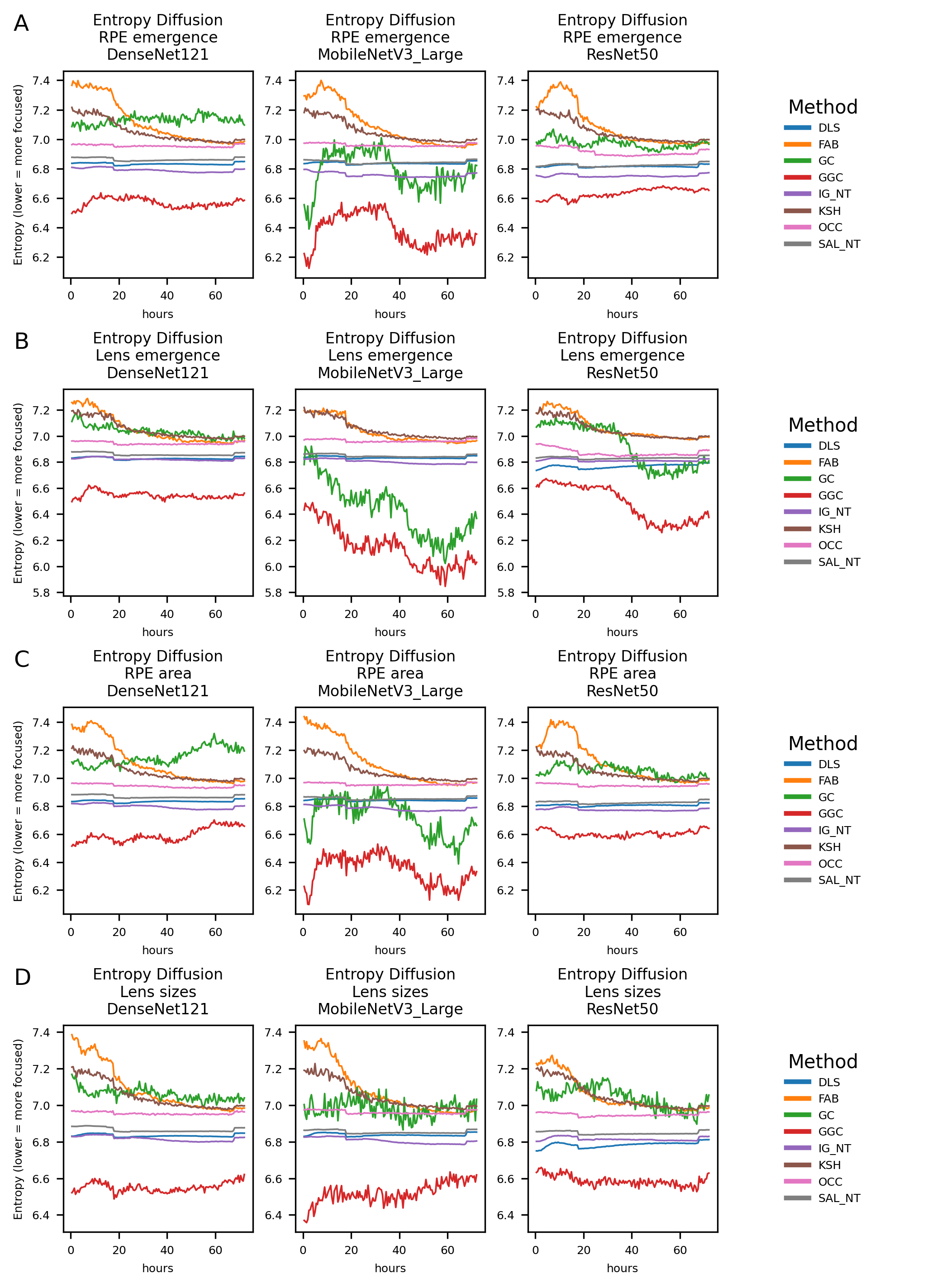

Supplement: S35 Fig — Entropy values of saliency maps were computed for three convolutional neural network architectures (DenseNet121, MobileNetV3_Large, ResNet50) and four readouts: (A) RPE emergence, (B) lens emergence, (C) RPE area, and (D) lens size. Eight attribution methods were compared: DeepLIFT SHAP (DLS), feature ablation (FAB), Grad-CAM (GC), guided Grad-CAM (GGC), integrated gradients (IG_NT), kernel SHAP (KSH), smooth occlusion (OCC), and simple saliency (SAL_NT). Entropy was calculated on the top 10% of saliency values inside the organoid mask (lower entropy = more focused attribution). Grad-CAM and guided Grad-CAM consistently showed the lowest entropy and further decreased over time, indicating increasingly focused saliency. FAB and kernel SHAP also decreased in several settings, while gradient-based (DLS, IG_NT, SAL_NT) and occlusion-based (OCC) methods remained more diffuse with higher entropy. Raw data of the figure plots have been deposited as Extended Data 91. (TIF) [file pbio.3003597.s038.tif]

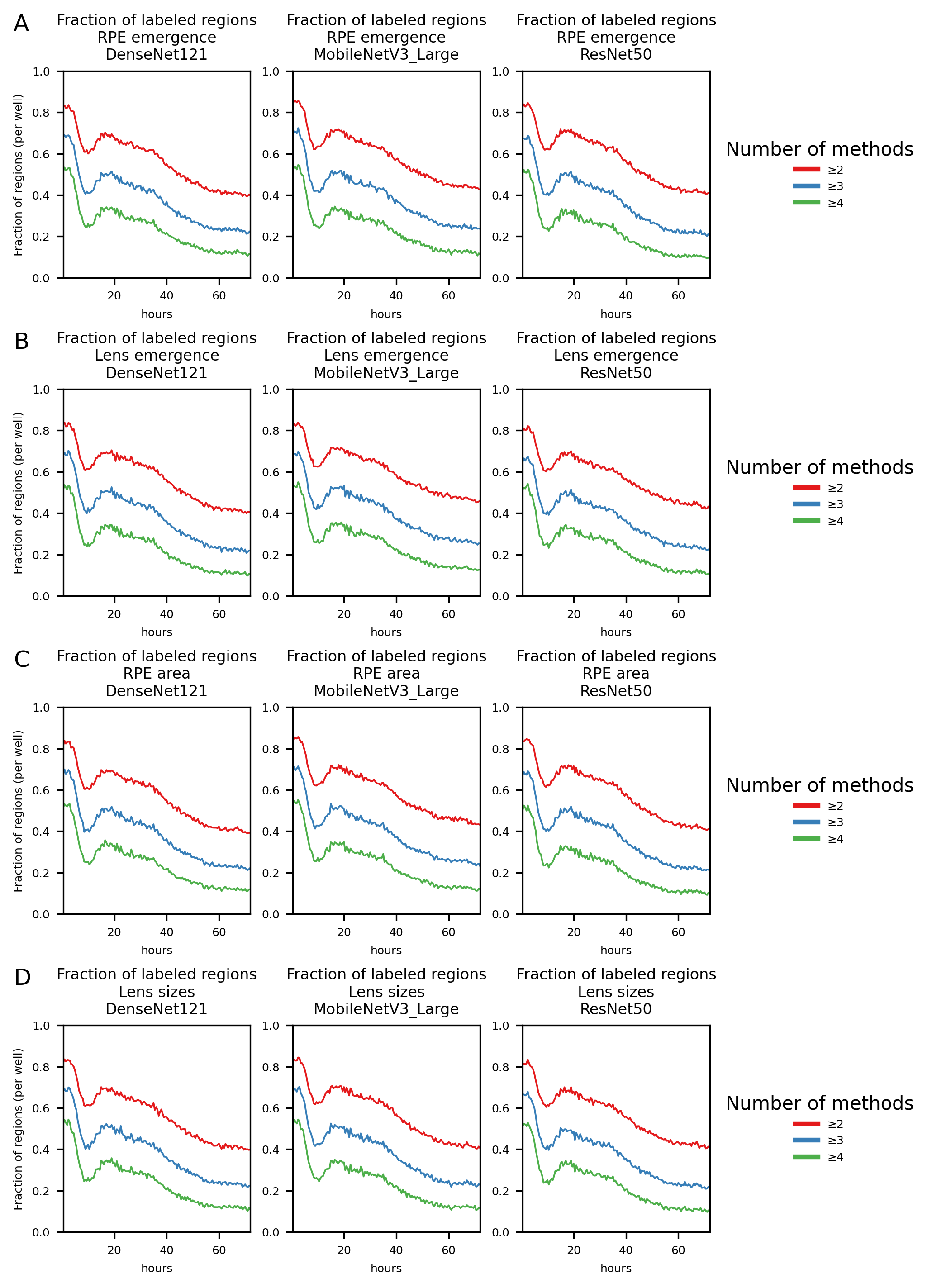

Supplement: S36 Fig — Saliency maps from three convolutional neural network architectures (DenseNet121, MobileNetV3_Large, ResNet50) were segmented into superpixels, and each method assigned votes to the top 10% most salient regions within the organoid mask. Shown is the fraction of regions per well that were consistently labeled by at least two (red), three (blue), or four (green) different attribution methods over time. Results are displayed for four readouts: (A) RPE emergence, (B) lens emergence, (C) RPE area, and (D) lens size. Agreement between methods was highest at early time points, with many regions receiving votes from multiple methods, but this overlap progressively declined as development advanced, indicating that saliency methods increasingly diverged in the regions they prioritized. Raw data of the figure plots have been deposited as Extended Data 92. (TIF) [file pbio.3003597.s039.tif]

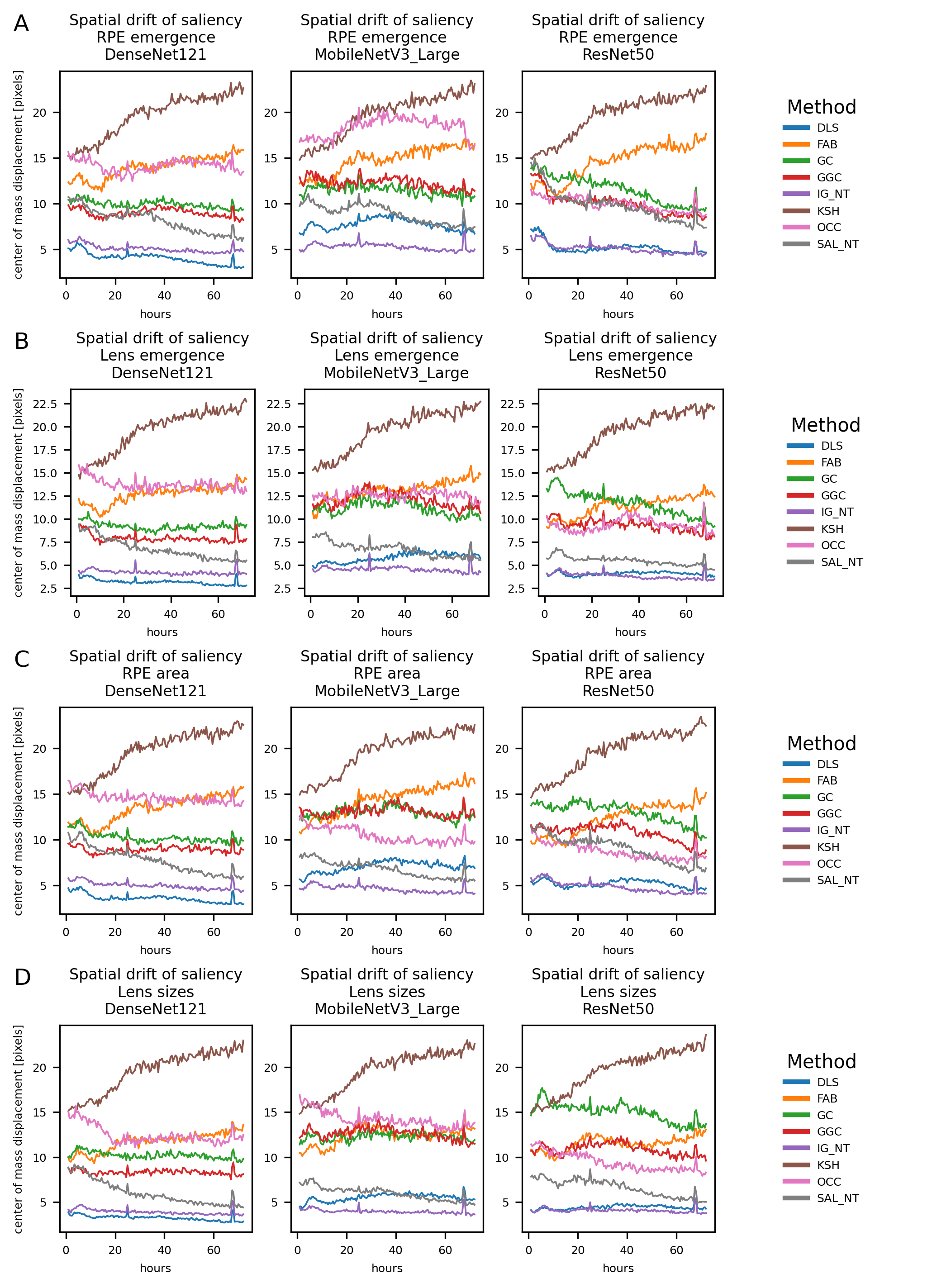

Supplement: S37 Fig — The displacement of the saliency map center of mass between consecutive timepoints is shown for three convolutional neural network architectures (DenseNet121, MobileNetV3_Large, ResNet50) across four prediction tasks: (A) RPE emergence, (B) lens emergence, (C) RPE area, and (D) lens size. Each attribution method is represented separately (DLS: DeepLIFT SHAP, IG_NT: Integrated Gradients, SAL_NT: Simple Saliency, GC: Grad-CAM, GGC: Guided Grad-CAM, OCC: Smooth Occlusion, FAB: Feature Ablation, KSH: Kernel SHAP). Lower drift values indicate that relevance remains in similar image regions across time, while higher values reflect more dynamic relocation of relevance. CAM-based methods (Grad-CAM, Guided Grad-CAM) and perturbation-based methods (Feature Ablation, Kernel SHAP) generally showed the strongest drift, whereas gradient-based methods (DeepLIFT SHAP, Integrated Gradients, Saliency) and Smooth Occlusion exhibited lower and more stable displacement over time. Raw data of the figure plots have been deposited as Extended Data 93. (TIF) [file pbio.3003597.s040.tif]
