# Supplementary material for: A deep learning-based computational pipeline predicts developmental outcome in retinal organoids
Source: PLoS Biol. 2026 Jan 27;24(1):e3003597. doi: 10.1371/journal.pbio.3003597 (PMC12843511; doi:10.1371/journal.pbio.3003597)
Supplement: Attachment — Submitted filename: 155345_1_rebuttal_3549172_t4b2tm.docx [file pbio.3003597.s042.docx]

**Manuscript number:** RC-2025-02933

**Corresponding author(s):** Joachim Wittbrodt and Tarik Exner

*[Please use this template only if the submitted manuscript should be considered by the affiliate journal as a full revision in response to the points raised by the reviewers.*

*If you wish to submit a preliminary revision with a revision plan, please use our "*[*Revision Plan*](https://docs.google.com/document/u/0/d/1HHp62Tfeb44nd3Gu_3ChB0b8gmMdEog7GjZcZFwHSiQ/edit)*" template.* ***It is important to use the appropriate template to clearly inform the editors of your intentions.****]*

## 1. General Statements

We thank the reviewers for their thoughtful and constructive feedback, which helped us strengthen the study on both the computational and biological side. In response, we added substantial new analyses and results in a total of 26 new supplementary figures and a new supplementary note. Importantly, we demonstrated that our approach generalizes beyond tissue outcomes by predicting final-timepoint morphology clusters from early frames with good accuracy as new Figure 4C. Furthermore, we completely restructured and expanded the human expert panel: six experts now provided >30,000 annotations across evenly spaced time intervals, allowing us to benchmark human predictions against CNNs and classical models under comparable conditions. We verified that morphometric trajectories are robust: PCA-based reductions and nearest-neighbor checks confirmed that patterns seen in t-SNE/UMAP are genuine, not projection artifacts. To test whether z-stacks are required, we re-did all analyses with sum- and maximum-intensity projections across five slices; results were unchanged, showing that single-slice imaging is sufficient. From a bioinformatics perspective, we performed negative-label baselines, downsampling analyses to quantify dataset needs, and statistical tests confirming CNNs significantly outperform classical models. Biologically, we clarified that each well contains one organoid, further introduced the Latent Determination Horizon concept tied to expert visibility thresholds, and discussed limits in cross-experiment transfer alongside strategies for domain adaptation and adaptive interventions. Finally, we clarified methods, corrected terminology and a scaler leak, and made all code and raw data publicly available.

Together, these revisions in our opinion provide an even clearer, more reproducible, and stronger case for the utility of predictive modeling in retinal organoid development.

## 2. Point-by-point description of the revisions

*This section is mandatory.* *Please insert a point-by-point reply describing the revisions that were already carried out and included in the transferred manuscript.*

------------------------------------------------------------------------------
Reviewer #1 (Evidence, reproducibility and clarity (Required)):

This study presents predictive modeling for developmental outcome in retinal organoids based on high-content imaging. Specifically, it compares the predictive performance of an ensemble of deep learning models with classical machine learning based on morphometric image features and predictions from human experts for four different task: prediction of RPE presence and lense presence (at the end of development) as well as the respective sizes. It finds that the DL model outperforms the other approaches and is predictive from early timepoints on, strongly indicating a time-frame for important decision steps in the developmental trajectory.

Response: We thank the reviewer for the constructive and thoughtful feedback. In response to the review as found below, we have made substantial revisions and additions to the manuscript. Specifically, we clarified key aspects of the experimental setup, changed terminology regarding training/validation/test sets, and restructured our human expert baseline analysis by collecting and integrating a substantially larger dataset of expert annotations according to suggestion. We introduced the Latent Determination Horizon concept with clearer rationale and grounding. Most importantly, we significantly expanded our interpretability analyses across three CNN architectures and eight attribution methods, providing comprehensive quantitative evaluations and supplementary figures that extend beyond the initial DenseNet121 examples (new Supplementary Figures S29-S37). We also ensured full reproducibility by making both code and raw data publicly available with documentation. While certain advanced interpretability methods (e.g., Discover) could not be integrated despite considerable effort, we believe the revised manuscript presents a robust, well-documented, and carefully qualified analysis of CNN predictions in retinal organoid development.

Major comments: I find the paper over-all well written and easy to understand. The findings are relevant (see significance statement for details) and well supported. However, I have some remarks on the description and details of the experimental set-up, the data availability and reproducibility / re-usability of the data.

1. Some details about the experimental set-up are unclear to me. In particular, it seems like there is a single organoid per well, as the manuscript does not mention any need for instance segmentation or tracking to distinguish organoids in the images and associate them over time. Is that correct? If yes, it should be explicitly stated so. Are there any specific steps in the organoid preparation necessary to avoid multiple organoids per well? Having multiple organoids per well would require the aforementioned image analysis steps (instance segmentation and tracking) and potentially add significant complexity to the analysis procedure, so this information is important to estimate the effort for setting up a similar approach in other organoid cultures (for example cancer organoids, where multiple organoids per well are common / may not be preventable in certain experimental settings).

Response: We thank the reviewer for this question. We agree that these preprocessing steps would add more complexity to our presented preprocessing steps and would definitely be required in some organoid systems. In our experimental setup, there is only one organoid per well which forms spontaneously after cell seeding from (almost) all seeded cells. There are no additional steps necessary in order to ensure this behaviour in our setup. We amended the Methods section to now explicitly state this accordingly (paragraph ‘Organoid timelapse imaging’).

2. The terminology used with respect to the test and validation set is contrary to the field, and reporting the results on the test set (should be called validation set), should be avoided since it is used to select models. In more detail: the terms "test set" and "validation set" (introduced in 213-221) are used with the opposite meaning to their typical use in the deep learning literature. Typically, the validation set refers to a separate split that is used to monitor convergence / avoid overfitting during training, and the test set refers to an external set that is used to evaluate the performance of trained models. The study uses these terms in an opposite manner, which becomes apparent from line 624: "best performing model ... judged by the loss of the test set.". Please exchange this terminology, it is confusing to a machine learning domain expert. Furthermore, the performance on the test set (should be called validation set) is typically not reported in graphs, as this data was used for model selection, and thus does not provide an unbiased estimate of model performance. I would remove the respective curves from Figures 3 and 4.

Response: We are thankful for the reviewers comments on this matter. Indeed, we were using an opposite terminology compared to what is commonly used within the field. We have adjusted the Results, Discussion and Methods sections as well as the figures accordingly. Further, we added a corresponding disclaimer for the code base in the github repository. However, we prefer to not remove the respective curves from the figures. We think that this information is crucial to interpret the variability in accuracy between organoids from the same experiments and organoids acquired from a different, independent experiment. The results suggest that the accuracy for organoids within the same experiments is still higher, indicating to users the potential accuracy drop resulting from independent experiments. As we think that this is crucial information for the interpretability of our results, we would like to still include it side-by-side with the test data in the figures.

3. The experimental set-up for the human expert baseline is quite different to the evaluation of the machine learning models. The former is based on the annotation of 4,000 images by seven expert, the latter based on a cross-validation experiments on a larger dataset. First of all, the details on the human expert labeling procedure is very sparse, I could only find a very short description in the paragraph 136-144, but did not find any further details in the methods section. Please add a methods section paragraph that explains in more detail how the images were chosen, how they were assigned to annotators, and if there was any redundancy in annotation, and if yes how this was resolved / evaluated. Second, the fact that the set-up for human experts and ML models is quite different means that these values are not quite comparable in a statistical sense. Ideally, human estimators would follow the same set-up as in ML (as in, evaluate the same test sets). However, this would likely prohibitive in the required effort, so I think it's enough to state this fact clearly, for example by adding a comment on this to the captions of Figure 3 and 4.

Response: We thank the reviewer for this constructive suggestion. We agree that the curves for human evaluations in the original draft were calculated differently compared to the curves for the classification algorithms, mostly stemming from feasibility of data set annotation at the time. In order to still address this suggestion, we went on to repeat and substantially expand the number of images annotated and thus revised the full human expert annotation. Each one of 6 human experts was asked to predict/interpret 6 images of each organoid within the full dataset. In order to select the images, we divided the time course (0-72h) into 6 evenly spaced intervals of 12 hours. For each interval, one image per organoid and human expert was randomly selected and assigned. This resulted in a total of 31,626 classified images (up from 4000 in the original version of the manuscript), from which the assigned images were overlapping between experts for each source interval but not for the individual images. We then changed the calculation of the curves to be the same as for the classification analysis: F1 data were calculated for each experiment over 6 timeframes and all experts, and plotted within the respective figure. We have amended the Methods section accordingly and replaced the respective curves within Figures 3 and 4 and Supplementary Figures S1, S8 and S19.

4. It is unclear to me where the theoretical time window for the Latent Determination Horizon in Figure 5 (also mentioned in line 350) comes from? Please explain this in more detail and provide a citation for it.

Response: We thank the reviewer for this important point. The *Latent Determination Horizon (LDH)* is a conceptual framework we introduced in this study to describe the theoretical period during which the eventual presence of a tissue outcome of interest (TOI) is being determined but not yet detectable. It is derived from two main observations in our dataset: (i) the inherent intra- and inter-experimental heterogeneity of organoid outcomes despite standardized protocols, and (ii) the progressive increase in predictive performance of our deep learning models over time, which suggests that informative morphological features only emerge gradually. We have now clarified this rationale in the manuscript (Discussion section) further and explicitly stated that the LDH is a concept we introduce here, rather than a previously described or cited term.

The timewindow is defined by the TOI visibility, which is defined empirically as indicated by the results of our human expert panel (compare also Supplementary Figure S1).

5. The intepretability analysis (Figure 4, 634-639) based on relevance backpropagation was performed based on DenseNet121 only. Why did you choose this model and not the ResNet / MobileNet? I think it is quite crucial to see if there are any differences between these model, as this would show how much weight can be put on the evidence from this analysis and I would suggest to add an additional experiment and supplementary figure on this.

Response: We thank the reviewer for this important comment regarding the interpretability analysis and the choice of model. In the original submission, we restricted the attribution analyses shown in originial Figure 4C to DenseNet121, which served as our main reference model throughout the study. This choice was made primarily for clarity and to avoid redundancy in the main figures, as all three convolutional neural network (CNN) architectures (DenseNet121, ResNet50, MobileNetV3_Large) achieved comparable classification performance on our tasks.

In response to the reviewer’s concern, we have now extended the interpretability analyses to include all three CNN architectures and a total of eight attribution methods (new Supplementary Note 1). Specifically, we generated saliency maps for DenseNet121, ResNet50, and MobileNetV3_Large across multiple time points and evaluated them using a systematic set of metrics: pairwise method agreement within each model (new Supplementary Figure S29), cross-model consistency per method (new Supplementary Figure S34), entropy and diffusion of saliencies over time (new Supplementary Figure S35), regional voting overlap across methods (new Supplementary Figure S36), and spatial drift of saliency centers of mass (new Supplementary Figure S37).

These pooled analyses consistently showed that attribution methods differ markedly in the regions they prioritize, but that their relative behaviors were mostly stable across the three CNN architectures. For example, Grad-CAM and Guided Grad-CAM exhibited strong internal agreement and progressively focused relevance into smaller regions, while gradient-based methods such as DeepLiftSHAP and Integrated Gradients maintained broader and more diffuse relevance patterns but were the most consistent across models. Perturbation-based methods like Feature Ablation and Kernel SHAP often showed decreasing entropy and higher spatial drift, again similarly across architectures.

To further address the reviewer’s point, we visualized the organoid depicted in original Figure 4C across all three CNNs and all eight attribution methods (new Supplementary Figures S30-S33). These comparisons confirm and extend analysis of the qualitative patterns described in original Figure 4C and show that they are not specific to DenseNet121, but are representative of the general behavior across architectures.

In sum, we observed notable differences in how relevance was assigned and how consistently these assignments aligned. Highlighted organoid patterns were not consistent enough across attribution methods for us to be comfortable to base unequivocal biological interpretation on them. Nevertheless we believe that the analyses in response to the reviewer’s suggestions (new Supplementary Note 1 and new Supplementary Figures S29-S37) add valuable context to what can be expected from machine learning models in an organoid research setting.

As we did not base further unequivocal biological claims on the relevance backpropagation, we decided to move the analyses to the Supporting Information and now show a new model predicting organoid morphology by morphometrics clustering at the final imaging timepoint in new Figure 4C in line with suggestions by Reviewer #3.

6. The code referenced in the code availability statement is not yet present. Please make it available and ensure a good documentation for reproducibility. Similarly, it is unclear to me what is meant by "The data that supports the findings will be made available on HeiDoc". Does this only refer to the intermediate results used for statistical analysis? I would also recommend to make the image data of this study available. This could for example be done through a dedicated data deposition service such as BioImageArchive or BioStudies, or with less effort via zenodo. This would ensure both reproducibility as well as potential re-use of the data. I think the latter point is quite interesting in this context; as the authors state themselves it is unclear if prediction of the TOIs isn't even possible at an earlier point that could be achieved through model advances, which could be studied by making this data available.

Response: We thank the reviewer for this comment. We have now made the repository and raw data public on the suggested platform (Zenodo) and apologize for this oversight. The links are contained within the github repository which is stated in the manuscript under “Data availability”.

Minor comments:

Line 315: Please add a citation for relevance backpropagation here.

Response: We have included citations for all relevance backpropagation methods used in the paper.

Line 591: There seems to be typo: "[...] classification of binary classification [...]"

Response: Corrected as suggested.

Line 608: "[...] where the images of individual organoids served as groups [...]" It is unclear to me what this means.

Response: We wanted to express that organoid images belonging to one organoid were assigned in full to a training/validation set. We have now stated this more clearly in the Methods section.

Reviewer #1 (Significance (Required)):

General assessment: This study demonstrates that (retinal) organoid development can be predicted from early timepoints with deep learning, where these cannot be discerned by human experts or simpler machine learning models. This fact is very interesting in itself due to its implication for organoid development, and could provide a valuable tool for molecular analysis of different organoid populations, as outlined by the authors. The contribution could be strengthened by providing a more thorough investigation of what features in the image are predictive at early timepoints, using a more sophisticated approach than relevance backprop, e.g. Discover (https://www.nature.com/articles/s41467-024-51136-9). This could provide further biological insight into the underlying developmental processes and enhance the understanding of retinal organoid development.

Response: We thank the reviewer for this assessment and suggestion. We agree that identifying image features predictive at early timepoints would add important biological context. We therefore attempted to apply *Discover* to our dataset. However, we were unable to get the system to run successfully. After considerable effort, we concluded that this approach could not be integrated into our current analysis. Instead, we report our substantially expanded results obtained with relevance backpropagation, which provided the most interpretable and reproducible insights for our study as described above (New Supplementary Note 1, new Supplementary Figures S29-S37).

Advance: similar studies that predict developmental outcome based on image data, for example cell proliferation or developmental outcome exist. However, to the best of my knowledge, this study is the first to apply such a methodology to organoids and convincingly shows is efficacy and argues is potential practical benefits. It thus constitutes a solid technical advance, that could be especially impactful if it could be translated to other organoid systems in the future.

Response: We thank the reviewer for this positive assessment of our work and for highlighting its novelty and potential impact. We are encouraged that the reviewer recognizes the value of applying predictive modeling to organoids and the opportunities this creates for translation to other organoid systems.

Audience: This research is of interest to a technical audience. It will be of immediate interest to researchers working on retinal organoids, who could adapt and use the proposed system to support experiments by better distinguishing organoids during development. To enable this application, code and data availability should be ensured (see above comments on reproducibility). It is also of interest to researchers in other organoid systems, who may be able to adapt the methodology to different developmental outcome predictions. Finally, it may also be of interest to image analysis / deep learning researchers as a dataset to improve architectures for predictive time series modeling.

My research background: I am an expert in computer vision and deep learning for biomedical imaging, especially in microscopy. I have some experience developing image analysis for (cancer) organoids. I don't have any experience on the wet lab side of this work.

Response: We thank the reviewer for this encouraging feedback and for recognizing the broad relevance of our work across retinal organoid research, other organoid systems, and the image analysis community. We are pleased that the potential utility of our dataset and methodology is appreciated by experts in computer vision and biomedical imaging. We have now made the repository and raw data public and apologize for this oversight. The links are provided in the manuscript under “Data availability”.

Constantin Pape

Reviewer #2 (Evidence, reproducibility and clarity (Required)):

Summary: Afting et al. present a computational pipeline for analyzing timelapse brightfield images of retinal organoids derived from Medaka fish. Their pipeline processes images along two paths: 1) morphometrics (based on computer vision features from skimage) and 2) deep learning. They discovered, through extensive manual annotation of ground truth, that their deep learning method could predict retinal pigmented epithelium and lens tissue emergence in time points earlier than either morphometrics or expert predictions. Our review is formatted based on the review commons recommendation.

Response: We thank the reviewer for the detailed and constructive feedback, which has greatly improved the clarity and rigor of our manuscript. In response, we have corrected a potential data leakage issue, re-ran the affected analyses, and confirmed that results remain unchanged. We clarified the use of data augmentation in CNN training, tempered some claims throughout the text, and provided stronger justification for our discretization approach together with new supplementary analyses (New Supplementary Figures S26, S27). We substantially expanded our interpretability analyses across three CNN architectures and eight attribution methods, quantified their consistency and differences (new Supplementary Figures S29, S34-S37, new Supplementary Note 1), and added comprehensive visualizations (New S30-S33). We also addressed technical artifact controls, provided downsampling analyses to support our statement on sample size sufficiency (new Supplementary Figure S28), and included negative-control baselines with shuffled labels in Figures 3 and 4. Furthermore, we improved the clarity of terminology, figures, and methodological descriptions, and we have now made both code and raw data publicly available with documentation. Together, we believe these changes further strengthen the robustness, reproducibility, and interpretability of our study while carefully qualifying the claims.

Major comments:

Are the key conclusions convincing?

Yes, the key conclusion that deep learning outperforms morphometric approaches is convincing. However, several methodological details require clarification. For instance, were the data splitting procedures conducted in the same manner for both approaches? Additionally, the authors note in the methods: "The validation data were scaled to the same range as the training data using the fitted scalers obtained from the training data." This represents a classic case of data leakage, which could artificially inflate performance metrics in traditional machine learning models. It is unclear whether the deep learning model was subject to the same issue. Furthermore, the convolutional neural network was trained with random augmentations, effectively increasing the diversity of the training data. Would the performance advantage still hold if the sample size had not been artificially expanded through augmentation?

Response: We thank the reviewer for raising these important methodological points. As Reviewer #1 correctly noted, our use of the terms *validation* and *test* may have contributed to confusion. To clarify: in the original analysis the scalers were fitted on the training and validation data and then applied to the test data. This indeed constitutes a form of data leakage. We have corrected the respective code, re-ran all analyses that were potentially affected, and did not observe any meaningful change in the reported results. The Methods section has been amended to clarify this important detail.

For the neural networks, each image was normalized independently (per image), without using dataset-level statistics, thereby avoiding any risk of data leakage.

Regarding data augmentation, the convolutional neural network was indeed trained with augmentations. Early experiments without augmentation led to severe overfitting, confirming that the performance advantage would not hold without artificially increasing the effective sample size. We have added a clarifying statement in the Methods section to make this explicit.

Should the authors qualify some of their claims as preliminary or speculative, or remove them altogether?
Their claims are currently preliminary, pending increased clarity and additional computational experiments described below.

Response: We believe our additionally performed computational experiments qualify all the claims we make in the revised version of the manuscript.

Would additional experiments be essential to support the claims of the paper? Request additional experiments only where necessary for the paper as it is, and do not ask authors to open new lines of experimentation.

- The authors discretize continuous variables into four bins for classification. However, a regression framework may be more appropriate for preserving the full resolution of the data. At a minimum, the authors should provide a stronger justification for this binning strategy and include an analysis of bin performance. For example, do samples near bin boundaries perform comparably to those near the bin centers? This would help determine whether the discretization introduces artifacts or obscures signals.

Response: We thank the reviewer for this thoughtful suggestion. We agree that regression frameworks can, in principle, preserve the full resolution of continuous outcome variables. However, in our setting we deliberately chose a discretization approach. First, the discretized outcome categories correspond to ranges of tissue sizes that are biologically meaningful and allow direct comparison to expert annotations. In practice, human experts also tend to judge tissue presence and size in categorical rather than strictly continuous terms, which was mirrored by our human expert annotation strategy. As we aimed to compare deep learning with classical machine learning models and with expert annotations across the same prediction tasks, a categorical outcome formulation provided the most consistent and fair framework. Secondly, the underlying outcome variables did not follow a normal distribution, but instead exhibited a skewed and heterogeneous spread. Regression models trained on such distributions often show biases toward the most frequent value ranges, which may obscure less common but biologically important outcomes. Discretization mitigated this issue by balancing the prediction task across defined size categories.

In line with the reviewer’s request, we have now analyzed the performance in relation to the distance of each sample from the bin center. These results are provided as new Supplementary Figures S26 and S27. Interestingly, for the classical machine learning classifiers, F1 scores tended to be somewhat higher for samples close to bin edges. For the convolutional neural networks, however, F1 scores were more evenly distributed across distances from bin centers. While the reason for this difference remains unclear, the analysis demonstrates that the discretization did not obscure predictive signals in either framework. We have amended the results section accordingly.

- The relevance backpropagation interpretation analysis is not convincing. The authors argue that the model's use of pixels across the entire image (rather than just the RPE region) indicates that the deep learning approach captures holistic information. However, only three example images are shown out of hundreds, with no explanation for their selection, limiting the generalizability of the interpretation. Additionally, it is unclear how this interpretability approach would work at all in earlier time points, particularly before the model begins making confident predictions around the 8-hour mark. It is also not specified whether the input used for GradSHAP matches the input used during CNN training. The authors should consider expanding this analysis by quantifying pixel importance inside versus outside annotated regions over time. Lastly, Figure 4C is missing a scale bar, which would aid in interpretability.

Response: We thank the reviewer for raising these important concerns. In the initial version we showed examples of relevance backpropagation that suggested CNNs rely on visible RPE or lens tissue for their predictions (original Figure 4C). Following the reviewer’s comment, we expanded the analysis extensively across all models and attribution methods (compare new Supplementary Note 1), and quantified agreement, consistency, entropy, regional overlap, and drift (new Supplementary Figures S29 and S34-S37), as well as providing comprehensive visualizations across models and methods (new Supplementary Figures S30-S33).

This extended analysis showed that attribution methods behave very differently from each other, but consistently so across the three CNN architectures. Each method displayed characteristic patterns, for example in entropy or center-of-mass drift, but the overlap between methods was generally low. While integrated gradients and DeepLiftSHAP tended to concentrate on tissue regions, other methods produced broader or shifting relevance patterns, and overall we could not establish robust or interpretable signals from a biological point of view that would support stronger conclusions.

We have therefore revised the text to focus on descriptive results only, without making claims about early structural information or tissue-specific cues being used by the networks. We also added missing scale bars and clarified methodological details. Together, the revised section now reflects the extensive work performed while remaining cautious about what can and cannot be inferred from saliency methods in this setting.

- The authors claim that they removed technical artifacts to the best of their ability, but it is unclear if the authors performed any adjustment beyond manual quality checks for contamination. Did the authors observe any illumination artifacts (either within a single image or over time)? Any other artifacts or procedures to adjust?

Response: We thank the reviewer for this comment. We have not performed any adjustment beyond manual quality control post organoid seeding. The aforementioned removal of technical artifacts included, among others, seeding at the same time of day, seeding and cell processing by the same investigator according to a standardized protocol, usage of reproducible chemicals (same LOT, frozen only once, etc.) and temperature control during image acquisition. We adhered strictly to internal, previously published workflows that were aimed to reduce any variability due to technical variations during cell harvesting, organoid preparation and imaging. We have clarified this important point in the Methods section.

- In line 434-436 the authors state "In this work, we used 1,000 organoids in total, to achieve the reported prediction accuracies. Yet, we suspect that as little as ~500 organoids are sufficient to reliably recapitulate our findings." It is unclear what evidence the authors use to support this claim? The authors could perform a downsampling analysis to determine tradeoff between performance and sample size.

Response: We thank the reviewer for this important comment. To clarify, our statement regarding the sufficiency of ~500 organoids was based on a downsampling-style analysis we had already performed. In this analysis, we systematically reduced the number of experiments used for training and assessed predictive performance for both CNN- and classifier-based approaches (former Supplementary Figure S11, new Supplementary Figure S28). For CNNs, performance curves plateaued at approximately six experiments (corresponding to ~500 organoids), suggesting that increasing the sample size further only marginally improved prediction accuracy. In contrast, we did not observe a clear plateau for the machine learning classifiers, indicating that these models can achieve comparable performance with fewer training experiments. We have revised the manuscript text to clarify that this conclusion is derived from these analyses, and continue to include Supplementary Figure S11 as new Supplementary Figure S28 for transparency (compare Supplementary Note 1).

Are the suggested experiments realistic in terms of time and resources? It would help if you could add an estimated cost and time investment for substantial experiments.
Yes, we believe all experiments are realistic in terms of time and resources. We estimate all experiments could be completed in 3-6 months.

Response: We confirm that the suggested experiments are realistic in terms of time and resources and have been able to complete them within 6 months.

Are the data and the methods presented in such a way that they can be reproduced?
No, the code is not currently available. We were not able to review the source code.

Response: We have now made the repository public. We apologize for this initial oversight. The links are provided in the revised version of the manuscript under “Data availability”.

Are the experiments adequately replicated and statistical analysis adequate?

- The experiments are adequately replicated.

- The statistical analysis (deep learning) is lacking a negative control baseline, which would be helpful to observe if performance is inflated.

Response: We thank the reviewer for this comment. We have calculated the respective curves with neural networks and machine learning classifiers that were trained on data with shuffled labels and have included these results as a separate curve in the respective Figures 3 and 4. We have also amended the Methods section accordingly.

Minor comments:

Specific experimental issues that are easily addressable.

Are prior studies referenced appropriately?

Yes.

Are the text and figures clear and accurate?

The authors must improve clarity on terminology. For example, they should define a comprehensive dataset, significant, and provide clarity on their morphometrics feature space. They should elaborate on what they mean by "confounding factor of heterogeneity".

Response: We thank the reviewer for highlighting the need to clarify terminology. We have revised the manuscript accordingly. Specifically, we now explicitly define *comprehensive dataset* as longitudinal brightfield imaging of ~1,000 organoids from 11 independent experiments, imaged every 30 minutes over several days, covering a wide range of developmental outcomes at high temporal resolution. Furthermore, we replaced the term *significantly* with wording that avoids implying statistical significance, where appropriate. We have clarified the morphometrics feature space in the Methods section in a more detailed fashion, describing the custom parameters that we used to enhance the regionprops_table function of skimage.

Do you have suggestions that would help the authors improve the presentation of their data and conclusions?
- Figure 2C describes a distance between what? The y axis is likely too simple. Same confusion over Figure 2D. Was distance computed based on tsne coordinates?

Response: We thank the reviewer for pointing out this potential source of confusion. The distances shown in original Figures 2C and 2D were not calculated in tSNE space. Instead, morphometrics features were first Z-scaled, and then dimensionality reduction by PCA was applied, with the first 20 principal components retaining ~93% of the variance. Euclidean distances were subsequently computed in this 20-dimensional PC space. For inter-organoid distances (Figure 2C), we calculated mean pairwise Euclidean distances between all organoids at each imaging time point, capturing the global divergence of organoid morphologies over time in an experiment-specific manner. For intra-organoid distances (Figure 2D), we calculated Euclidean distances between consecutive time points (n vs. n+1) for each individual organoid, thereby quantifying the extent of morphological change within organoids over time. We have revised the Figure legend and Methods section to make these definitions clearer.

- The authors perform a Herculean analysis comparing dozens of different machine learning classifiers. They select two, but they should provide justification for this decision.

Response: We thank the reviewer for this comment. In our initial machine learning analyses, we systematically benchmarked a broad set of classifiers on the morphometrics feature space, using cross-validation and hyperparameter tuning where appropriate. The classifiers that we ultimately focused on were those that consistently achieved the best performance in these comparisons. This process is described in the Methods and summarized in the Supplementary Figures S4 and S15 (for sum- and maximum-intensity z-projections new Supplementary Figures S5/6 and S16/17), which show the results of the benchmarking. We have clarified the text to state that the selected classifiers were chosen on the basis of their superior performance in these evaluations.

- It would be good to get a sense for how these retinal organoids grow - are they moving all over the place? They are in Matrigel so maybe not, but are they rotating?

Can the author's approach predict an entire non-emergence experiment? The authors tried to standardize protocol, but ultimately if It's deriving this much heterogeneity, then how well it will actually generalize to a different lab is a limitation.

Response: We thank the reviewer for these thoughtful questions. The retinal organoids in our study were embedded in low concentrations of Matrigel and remained relatively stable in position throughout imaging. We did not observe substantial displacement or lateral movement of organoids, and no systematic rotation could be detected in our dataset. Small morphological rearrangements within organoids were observed, but the gross positioning of organoids within the wells remained consistent across time-lapse recordings.

Regarding generalization across laboratories, we agree with the reviewer that this is an important limitation. While we minimized technical variability by adhering to a highly standardized, published protocol (see Methods), considerable heterogeneity remained at both intra- and inter-experimental levels. This variability likely reflects inherent properties of the system, similar the reportings in the literature across organoid systems, rather than technical artifacts, and poses a potential challenge for applying our models to independently generated datasets. We therefore highlight the need for future work to test the robustness of our models across laboratories, which will be essential to determine the true generalizability of our approach. We have amended the Discussion accordingly.

- The authors should dampen claims throughout. For example, in the abstract they state, "by combining expert annotations with advanced image analysis". The image analysis pipelines use common approaches.

Response: We thank the reviewer for this comment. We agree that the individual image analysis steps we used, such as morphometric feature extraction, are based on well-established algorithms. By referring to “advanced image analysis,” we intended to highlight not the novelty of each single algorithm, but rather the way in which we systematically combined a large number of quantitative parameters and leveraged them through machine learning models to generate predictive insights into organoid development.

- The authors state: "the presence of RPE and lenses were disagreed upon by the two independently annotating experts in a considerable fraction of organoids (3.9 % for RPE, 2.9% for lenses).", but it is unclear why there were two independently annotating experts. The supplements say images were split between nine experts for annotation.

Response: We thank the reviewer for pointing out this ambiguity. To clarify, the ground truth definition at the final time point was established by two experts who annotated all organoids. These two annotators were part of the larger group of six experts who contributed to the earlier human expert annotation tasks. Thus, while six experts provided annotations for subsets of images during the expert prediction experiments, the final annotation for every single organoid at its last time frame was consistently performed by the same two experts to ensure a uniform ground truth. We have amended this in the revised manuscript to make this distinction clear.

- Details on the image analysis pipeline would be helpful to clarify. For example, why did they choose to measure these 165 morphology features? Which descriptors were used to quantify blur? Did the authors apply blur metrics per FOV or per segmented organoid?

Response: We thank the reviewer for this comment. To clarify, we extracted 165 morphometric features per segmented organoid, combining standard *scikit-image* region properties with custom implementations (e.g., blur quantified as the variance of the Laplace filter response within the organoid mask). All metrics, including blur, were calculated per segmented organoid rather than per full field of view. This broad feature space was deliberately chosen to capture size, shape, and intensity distributions in a comprehensive and unbiased manner. We now provide a more detailed description of the preprocessing steps, the full feature list, and the exact code implementations are provided in the Methods section (“Large-scale time-lapse Image analysis”) of the revised version of the manuscript as well as in the source code github repository.

- The description of the number of images is confusing and distracts from the number of organoids. The number of organoids and number of timepoints used would provide a better description of the data with more value. For example, does this image count include all five z slices?

Response: We thank the reviewer for this comment. The reported image count includes slice 3 only, which we based our models on. The five z-slices that we used to create the MAX- and SUM-intensity z-projections would increase this number 5-fold. While we agree that the number of organoids and time points are highly informative metrics and have provided these details in the manuscript, we also believe that reporting the image count is valuable, as it directly reflects the size of the dataset processed by our analysis pipelines. For this reason, we prefer to keep the current description.

- The authors should consider applying a maximum projection across the five z slices (rather than the middle z) as this is a common procedure in image analysis. Why not analyze three-dimensional morphometrics or deep learning features? Might this improve performance further?

Response: We thank the reviewer for this valuable suggestion. To address this point, we repeated all analyses using both sum- and maximum-intensity z-projections and have included the results as new Supplementary Figures S8-S10, S13/S14 for TOI emergence and new Supplementary Figures S19-S21, S24/S25 for TOI sizes (classifier benchmarking and hyperparameter tuning in new Supplementary Figures S5/S6 and S16/S17). These additional analyses did not reveal a noticeable improvement in performance, suggesting that projections incorporating all slices are not strictly necessary in our setting. An analysis that included all five z-slices separately for classification would indeed be of interest, but was not feasible within the scope of this study, as it would substantially increase the computational demands beyond the available resources and timeframe.

- There is a lot of manual annotation performed in this work, the authors could speculate how this could be streamlined for future studies. How does the approach presented enable streamlining?

Response: We thank the reviewer for raising this important point. The current study relied on expert visual review, which is time-intensive, but our findings suggest several ways to streamline future work. For instance, model-assisted prelabeling could be used to automatically accept high-confidence cases while routing only uncertain cases to experts. Active sampling strategies, focusing expert review on boundary cases or rare classes, as well as programmatic checks from morphometrics (e.g., blur or contrast to flag low-quality frames), could further reduce effort. Consensus annotation could be reserved only for cases where the model and expert disagree or confidence is low. Finally, new experiments could be bootstrapped with a small seed set of annotated organoids for fine-tuning before switching to such a model-assisted workflow. These possibilities are enabled by our approach, where organoids are imaged individually, morphometrics provide automated quality indicators, and the CNN achieves reliable performance at early developmental stages, making model-in-the-loop annotation a feasible and efficient strategy for future studies. We have added a clarifying paragraph to the Discussion accordingly.

Reviewer #2 (Significance (Required)):

Describe the nature and significance of the advance (e.g. conceptual, technical, clinical) for the field.
The paper's advance is technical (providing new methods for organoid quality control) and conceptual (providing proof of concept that earlier time points contain information to predict specific future outcomes in retinal organoids)

Place the work in the context of the existing literature (provide references, where appropriate).

- The authors do a good job of placing their work in context in the introduction.
- The work presents a simple image analysis pipeline (using only the middle z slice) to process timelapse organoid images. So not a 4D pipeline (time and space), just 3D (time). It is likely that more and more of these approaches will be developed over time, and this article is one of the early attempts.

- The work uses standard convolutional neural networks.

Response: We thank the reviewer for this assessment. We agree that our work represents one of the early attempts in this direction, applying a straightforward pipeline with standard convolutional neural networks, and we appreciate the reviewer’s acknowledgment of how the study has been placed in context within the Introduction.

State what audience might be interested in and influenced by the reported findings.
- Data scientists performing image-based profiling for time lapse imaging of organoids.

- Retinal organoid biologists

- Other organoid biologists who may have long growth times with indeterminate outcomes.

Response: We thank the reviewer for outlining the relevant audiences. We agree that the reported findings will be of interest to data scientists working on image-based profiling, retinal organoid biologists, and more broadly to organoid researchers facing long culture times with uncertain developmental outcomes.

Define your field of expertise with a few keywords to help the authors contextualize your point of view. Indicate if there are any parts of the paper that you do not have sufficient expertise to evaluate.
- Image-based profiling/morphometrics

- Organoid image analysis

- Computational biology

- Cell biology

- Data science/machine learning

- Software

This is a signed review:

Gregory P. Way, PhD

Erik Serrano

Jenna Tomkinson

Michael J. Lippincott

Cameron Mattson

Department of Biomedical Informatics, University of Colorado

Reviewer #3 (Evidence, reproducibility and clarity (Required)):

Summary:

This manuscript by Afting et. al. addresses the challenge of heterogeneity in retinal organoid development by using deep learning to predict eventual tissue outcomes from early-stage images. The central hypothesis is that deep learning can forecast which tissues an organoid will form (specifically retinal pigmented epithelium, RPE, and lens) well before those tissues become visibly apparent. To test this, the authors assembled a large-scale time-lapse imaging dataset of ~1,000 retinal organoids (~100,000 images) with expert annotations of tissue outcomes. They characterized the variability in organoid morphology and tissue formation over time, focusing on two tissues: RPE (which requires induction) and lens (which appears spontaneously). The core finding is that a deep learning model can accurately predict the emergence and size of RPE and lens in individual organoids at very early developmental stages. Notably, a convolutional neural network (CNN) ensemble achieved high predictive performance (F1-scores ~0.85-0.9) hours before the tissues were visible, significantly outperforming human experts and classical image-analysis-based classifiers. This approach effectively bypasses the issue of stochastic developmental heterogeneity and defines an early "determination window" for fate decisions. Overall, the study demonstrates a proof-of-concept that artificial intelligence can forecast organoid differentiation outcomes non-invasively, which could revolutionize how organoid experiments are analyzed and interpreted.

Recommendation:

While this manuscript addresses an important and timely scientific question using innovative deep learning methodologies, it currently cannot be recommended for acceptance in its present form. The authors must thoroughly address several critical limitations highlighted in this report. In particular, significant issues remain regarding the generalizability of the predictive models across different experimental conditions, the interpretability of deep learning predictions, and the use of Euclidean distance metrics in high-dimensional morphometric spaces-potentially leading to distorted interpretations of organoid heterogeneity. These revisions are essential for validating the general applicability of their approach and enhancing biological interpretability. After thoroughly addressing these concerns, the manuscript may become suitable for future consideration.

Response: We thank the reviewer for the thoughtful and constructive comments. In response, we expanded our analyses in several key ways. We clarified limitations regarding external datasets. Interpretability analyses were greatly extended across three CNN architectures and eight attribution methods (new Supplementary Figures S29-S37, new Supplementary Note 1), showing consistent but method-specific behaviors; as no reproducible biologically interpretable signals emerged, we now present these results descriptively and clearly state their limitations. We further demonstrated the flexibility of our framework by predicting morphometric clusters in addition to tissue outcomes (new Figure 4C), confirmed robustness of the morphometrics space using PCA and nearest-neighbor analyses (new Supplementary Figure S3), and added statistical tests confirming CNNs significantly outperform classical classifiers (Supplementary File 1). Finally, we made all code and raw data publicly available, clarified species context, and added forward-looking discussion on adaptive interventions. We believe these revisions now further improve the rigor and clarity of our work.

Major Issues (with Suggestions):

1. Generalization to Other Batches or Protocols: The drop in performance on independent validation experiments suggests the model may partially overfit to specific experimental conditions. A major concern is how well this approach would work on organoids from a different batch or produced by a slightly different differentiation protocol. Suggestion: The authors should clarify the extent of variability between their "independent experiment" and training data (e.g., were these done months apart, with different cell lines or minor protocol tweaks?). To strengthen confidence in the model's robustness, I recommend testing the trained model on one or more truly external datasets, if available (for instance, organoids generated in a separate lab or under a modified protocol). Even a modest analysis showing the model can be adapted (via transfer learning or re-training) to another dataset would be valuable. If new data cannot be added, the authors should explicitly discuss this limitation and perhaps propose strategies (like domain adaptation techniques or more robust training with diverse conditions) to handle batch effects in future applications.

Response: We thank the reviewer for this important comment. We fully agree with the reviewer that this would be an amazing addition to the manuscript. Unfortunately we are not able to obtain the requested external data set. Although retinal organoid systems exist and are widely used across different species lines, to the best of our knowledge our laboratory is the only one currently raising retinal organoids from primary embryonic pluripotent stem cells of *Oryzias latipes* and there is currently only one known (and published) differentiation protocol which allows the successful generation of these organoids. We note that our datasets were collected over the course of nine months, which already introduces variability across time and thus partially addresses concerns regarding batch effects. While we did not have access to truly external datasets (e.g., from other laboratories), we have clarified this limitation as suggested in the revised version of the manuscript and outlined strategies such as domain adaptation and training on more diverse conditions as promising future directions to improve robustness.

2. Biological Interpretation of Early Predictive Features: The study currently concludes that the CNN picks up on complex, non-intuitive features that neither human experts nor conventional analysis could identify. However, from a biological perspective, it would be highly insightful to know what these features are (e.g., subtle texture, cell distribution patterns, etc.). Suggestion: I encourage the authors to delve deeper into interpretability. They might try complementary explainability techniques (for example, occlusion tests where parts of the image are masked to see if predictions change, or activation visualization to see what patterns neurons detect) beyond GradientSHAP. Additionally, analyzing false predictions might provide clues: if the model is confident but wrong for certain organoids, what visual traits did those have? If possible, correlating the model's prediction confidence with measured morphometrics or known markers (if any early marker data exist) could hint at what the network sees. Even if definitive features remain unidentified, providing the reader with any hypothesis (for instance, "the network may be sensing a subtle rim of pigmentation or differences in tissue opacity") would add value. This would connect the AI predictions back to biology more strongly.

Response: We thank the reviewer for this thoughtful suggestion. We agree that linking CNN predictions to specific biological features would be highly valuable. In response, we expanded our interpretability analyses beyond GradientSHAP to a broad set of attribution methods and quantified their behavior across models and timepoints (new Supplementary Figures S29-S37, new Supplementary Note 1). While some methods (e.g., Integrated Gradients, DeepLiftSHAP) occasionally highlighted visible tissue regions, others produced diffuse or shifting relevance, and overall overlap was low. Therefore, our results did not yield reproducible, interpretable biological signals.

Given these results, we have refrained from speculating about specific early image features and now present the interpretability analyses descriptively. We agree that future studies integrating imaging with molecular markers will be required to directly link early predictive cues to defined biological processes.

3. Expansion to Other Outcomes or Multi-Outcome Prediction: The focus on RPE and lens is well-justified, but these are two outcomes within retinal organoids. A major question is whether the approach could be extended to predict other cell types or structures (e.g., presence of certain retinal neurons, or malformations) or even multiple outcomes at once. Suggestion: The authors should discuss the generality of their approach. Could the same pipeline be trained to predict, say, photoreceptor layer formation or other features if annotated? Are there limitations (like needing binary outcomes vs. multi-class)? Even if outside the scope of this study, a brief discussion would reassure readers that the method is not intrinsically limited to these two tissues. If data were available, it would be interesting to see a multi-label classification (predict both RPE and lens presence simultaneously) or an extension to other organoid systems in future. Including such commentary would highlight the broad applicability of this platform.

Response: We thank the reviewer for this helpful and important suggestion. While our study focused on RPE and lens as the most readily accessible tissues of interest in retinal organoids, our new analyses demonstrate that the pipeline is not limited to these outcomes. In addition to tissue-specific predictions, we trained both a convolutional neural network (on image data) and a decision tree classifier (on morphometrics features) to predict more abstract morphological clusters defined at the final timepoint using the morphometrics features, showing that both approaches could successfully capture non-tissue features from early frames (new Figure 4C). This illustrates that the framework can be extended beyond binary tissue outcomes to multi-class problems, and predict relevant outcomes like the overall organoid morphology. Given appropriate annotations, the framework could in principle be trained to detect additional structures such as photoreceptor layers or malformations. Furthermore, the CNN architecture we employed and the morphometrics feature space are compatible with multi-label classification, meaning simultaneous prediction of several outcomes would also be feasible. We have clarified this point in the discussion to highlight the methodological flexibility and potential generality of our approach and are excited to share this very interesting, additional model with the readership.

4. Curse of high dimensionality: Using Euclidean distance in a 165-dimensional morphometric space likely suffers from the curse of dimensionality, which diminishes the meaning of distances as dimensionality increases. In such high-dimensional settings, the range of pairwise distances tends to collapse, undermining the ability to discern meaningful intra- vs. inter-organoid differences. Suggestion: To address this, I would encourage the authors to apply principal component analysis (PCA) in place of (or prior to) tSNE. PCA would reduce the data to a few dominant axes of variation that capture most of the morphometric variance, directly revealing which features drive differences between organoids. These principal components are linear combinations of the original 165 parameters, so one can examine their loadings to identify which morphometric traits carry the most information - yielding interpretable axes of biological variation (e.g., organoid size, shape complexity, etc.). In addition, I would like to mention an important cautionary remark regarding tSNE embeddings. tSNE does not preserve global geometry of the data. Distances and cluster separations in a tSNE map are therefore not faithful to the original high-dimensional distances and should be interpreted with caution. See Chari T, Pachter L (2023), The specious art of single-cell genomics, PLoS Comput Biol 19(8): e1011288, for an enlightening discussion in the context of single cell genomics. The authors have shown that extreme dimensionality reduction to 2D can introduce significant distortions in the data's structure, meaning the apparent proximity or separation of points in a tSNE plot may be an artifact of the algorithm rather than a true reflection of morphometric similarity. Implementing PCA would mitigate high-dimensional distance issues by focusing on the most informative dimensions, while also providing clear, quantitative axes that summarize organoid heterogeneity. This change would strengthen the analysis by making the results more robust (avoiding distance artifacts) and biologically interpretable, as each principal component can be traced back to specific morphometric features of interest.

Response: We thank the reviewer for this mention. Indeed, high dimensionality and dimensionality reductions can lead to false interpretations. We approached this issue as follows: First, we calculated the same TSNE projections and distances using the first 20 PCs and supplied these data as the new Figure 2 and new Supplementary Figure 2. While the scale of the data shifted slightly, there were no differences in the data distribution that would contradict our prior conclusions.

In order to confirm the findings and further emphasize the validity of our dimensionality reduction, we calculated the intersection of 30 nearest neighbors in raw data space (or pca space) compared and 30 nearest neighbors in reduced space (TSNE or UMAP, as we wanted to emphasize that this was not an effect specific for TSNE projections and would also be valid in a dimensionality reduction which is more known to preserve global structure rather than local structure). As shown in the new Supplementary Figure S3 (A-D), the high jaccard index confirmed that our projections accurately reflect the data’s structure obtained from raw distance measurements. Moreover, the jaccard index generally increased over time, which is best explained by a stronger morphological similarity of organoids at timepoint 0 and reflected by the dense point cloud in the TSNE projections at that timepoint. The described effects were independent of the usage of data derived from 20 PCs versus data derived from all 165 dimensions.

We next wanted to confirm the conclusion that data points obtained from organoids at later timepoints were more closely related to each other than data points from different organoids. We therefore identified the 30 nearest neighbor data points, showing that at later timepoints these 30 nearest neighbor data points were almost all attributable to the same organoid (new Supplementary Figure S3 E/F). This was only not the case for experiments that lacked in between timepoints (E007 and E002), therefore misaligning the organoids in the reduced space and convoluting the nearest neighbor analysis.

We have included the respective new Figures and new Supplementary Figures and linked them in the main manuscript.

5. Statistical Reporting and Significance: The manuscript focuses on F1-score as the metric to report accuracy over time, which is appropriate. However, it's not explicitly stated whether any statistical significance tests were performed on the differences between methods (e.g., CNN vs human, CNN vs classical ML). Suggestion: The authors could report statistical significance of the performance differences, perhaps using a permutation test or McNemar's test on predictions. For example, is the improvement of the CNN ensemble over the Random Forest/QDA classifier statistically significant across experiments? Given the n of organoids, this should be assessable. Demonstrating significance would add rigor to the analysis.

Response: We thank the reviewer for this helpful suggestion. Following the recommendation, we quantified per-experiment differences in predictive performance by calculating the area under the F1-score curves (AUC) for each classifier and experiment. We then compared methods using paired Wilcoxon signed-rank tests across experiments, with Holm-Bonferroni correction for multiple comparisons. This analysis confirmed that the CNN consistently and significantly outperformed the baseline models and classical machine learning classifiers in validation and test organoids, while CNNs were notably but not significantly better performing in test organoids for RPE area and lens sizes compared to the machine learning classifiers. In summary, the findings add the requested statistical rigor to our findings. The results of these tests are now provided in the Supplementary Material as Supplementary File 1.

Minor Issues (with Suggestions):

1. Data Availability: Given the resource-intensive nature of the work, the value to the community will be highest if the data is made publicly available. I understand that this is of course at the behest of the authors and they do mention that they will make the data available upon publication of the manuscript. For the time being, the authors can consider sharing at least a representative subset of the data or the trained model weights. This will allow others to build on their work and test the method in other contexts, amplifying the impact of the study.

Response: We have now made the repository and raw data public and apologize for this oversight. The link for the github repository is now provided in the manuscript under “Data availability”, while the links for the datasets are contained within the github repository.

2. Discussion - Future Directions: The Discussion does a good job of highlighting applications (like guiding molecular analysis). One minor addition could be speculation on using this approach to actively intervene: for example, could one imagine altering culture conditions mid-course for organoids predicted not to form RPE, to see if their fate can be changed? The authors touch on reducing variability by focusing on the window of determination; extending that thought to an experimental test (though not done here) would inspire readers. This is entirely optional, but a sentence or two envisioning how predictive models enable dynamic experimental designs (not just passive prediction) would be a forward-looking note to end on.

Response: We thank the reviewer for this constructive suggestion. We have expanded the discussion to briefly address how predictive modeling could go beyond passive observation. Specifically, we now discuss that predictive models may enable dynamic interventions, such as altering culture conditions mid-course for organoids predicted not to form RPE, to test whether their developmental trajectory can be redirected. While outside the scope of the present work, this forward-looking perspective emphasizes how predictive modeling could inspire adaptive experimental strategies in future studies.

I believe with the above clarifications and enhancements - especially regarding generalizability and interpretability - the paper will be suitable for broad readership. The work represents an exciting intersection of developmental biology and AI, and I commend the authors for this contribution.

Response: We thank the reviewer for the positive assessment and their encouraging remarks regarding the contribution of our work to these fields.

Novelty and Impact:

This work fills an important gap in organoid biology and imaging. Previous studies have used deep learning to link imaging with molecular profiles or spatial patterns in organoids, but there remained a "notable gap" in predicting whether and to what extent specific tissues will form in organoids. The authors' approach is novel in applying deep learning to prospectively predict organoid tissue outcomes (RPE and lens) on a per-organoid basis, something not previously demonstrated in retinal organoids. Conceptually, this is a significant advance: it shows that fate decisions in a complex 3D culture model can be predicted well in advance, suggesting the existence of subtle early morphogenetic cues that only a sophisticated model can discern. The findings will be of broad interest to researchers in organoid technology, developmental biology, and biomedical AI.

Response: We thank the reviewer for this thoughtful and encouraging assessment. We agree that our study addresses an important gap by prospectively predicting tissue outcomes at the single-organoid level, and we appreciate the recognition that this represents a conceptual advance with relevance not only for retinal organoids but also for broader applications in organoid biology, developmental biology, and biomedical AI.

Methodological Rigor and Technical Quality:

The study is methodologically solid and carefully executed. The authors gathered a uniquely large dataset under consistent conditions, which lends statistical power to their analyses. They employ rigorous controls: an expert panel provided human predictions as a baseline, and a classical machine learning pipeline using quantitative image-derived features was implemented for comparison. The deep learning approach is well-chosen and technically sound. They use an ensemble of CNN architectures (DenseNet121, ResNet50, and MobileNetV3) pre-trained on large image databases, fine-tuning them on organoid images. The use of image segmentation (DeepLabV3) to isolate the organoid from background is appropriate to ensure the models focus on the relevant morphology. Model training procedures (data augmentation, cross-entropy loss with class balancing, learning rate scheduling, and cross-validation) are thorough and follow best practices. The evaluation metrics (primarily F1-score) are suitable for the imbalanced outcomes and emphasize prediction accuracy in a biologically relevant way. Importantly, the authors separate training, test, and validation sets in a meaningful manner: images of each organoid are grouped to avoid information leakage, and an independent experiment serves as a validation to test generalization. The observation that performance is slightly lower on independent validation experiments underscores both the realism of their evaluation and the inherent heterogeneity between experimental batches. In addition, the study integrates interpretability (using GradientSHAP-based relevance backpropagation) to probe what image features the network uses. Although the relevance maps did not reveal obvious human-interpretable features, the attempt reflects a commendable thoroughness in analysis. Overall, the experimental design, data analysis, and reporting are of high quality, supporting the credibility of the conclusions.

Response: We thank the reviewer for their very positive and detailed assessment. We appreciate the recognition of our efforts to ensure methodological rigor and reproducibility, and we agree that interpretability remains an important but challenging area for future work.

Reviewer #3 (Significance (Required)):

Scientific Significance and Conceptual Advances:

Biologically, the ability to predict organoid outcomes early is quite significant. It means researchers can potentially identify when and which organoids will form a given tissue, allowing them to harvest samples at the right moment for molecular assays or to exclude organoids that will not form the desired structure. The manuscript's results indicate that RPE and lens fate decisions in retinal organoids are made much earlier than visible differentiation, with predictive signals detectable as early as ~11 hours for RPE and ~4-5 hours for lens. This suggests a surprising synchronization or early commitment in organoid development that was not previously appreciated. The authors' introduction of deep learning-derived determination windows refines the concept of a developmental "point of no return" for cell fate in organoids. Focusing on these windows could help in pinpointing the molecular triggers of these fate decisions. Another conceptual advance is demonstrating that non-invasive imaging data can serve a predictive role akin to (or better than) destructive molecular assays. The study highlights that classical morphology metrics and even expert eyes capture mainly recognition of emerging tissues, whereas the CNN detects subtler, non-intuitive features predictive of future development. This underlines the power of deep learning to uncover complex phenotypic patterns that elude human analysis, a concept that could be extended to other organoid systems and developmental biology contexts. In sum, the work not only provides a tool for prediction but also contributes conceptual insights into the timing of cell fate determination in organoids.

Response: We thank the reviewer for this thoughtful and positive assessment. We agree that the determination windows provide a valuable framework to study early fate decisions in organoids, and we have emphasized this point in the discussion to highlight the biological significance of our findings.

Strengths:

The combination of high-resolution time-lapse imaging with advanced deep learning is innovative. The authors effectively leverage AI to solve a biological uncertainty problem, moving beyond qualitative observations to quantitative predictions. The study uses a remarkably large dataset (1,000 organoids, >100k images), which is a strength as it captures variability and provides robust training data. This scale lends confidence that the model isn't overfit to a small sample. By comparing deep learning with classical machine learning and human predictions, the authors provide context for the model's performance. The CNN ensemble consistently outperforms both the classical algorithms and human experts, highlighting the value added by the new method. The deep learning model achieves high accuracy (F1 > 0.85) at impressively early time points. The fact that it can predict lens formation just ~4.5 hours into development with confidence is striking. Performance remained strong and exceeded human capability at all assessed times. Key experimental and analytical steps (segmentation, cross-validation between experiments, model calibration, use of appropriate metrics) are executed carefully. The manuscript is transparent about training procedures and even provides source code references, enhancing reproducibility. The manuscript is generally well-written with a logical flow from the problem (organoid heterogeneity) to the solution (predictive modeling) and clear figures referenced.

Response: We thank the reviewer for this very positive and encouraging assessment of our study, particularly regarding the scale of our dataset, the methodological rigor, and the reproducibility of our approach.

Weaknesses and Limitations:

Generalizability Across Batches/Conditions: One limitation is the variability in model performance on organoids from independent experiments. The CNN did slightly worse on a validation set from a separate experiment, indicating that differences in the experimental batch (e.g., slight protocol or environmental variations) can affect accuracy. This raises the question of how well the model would generalize to organoids generated under different protocols or by other labs. While the authors do employ an experiment-wise cross-validation, true external validation (on a totally independent dataset or a different organoid system) would further strengthen the claim of general applicability.

Response: We thank the reviewer for this important point. We agree that generalizability across batches and experimental conditions is a key consideration. We have carefully revised the discussion to explicitly address this limitation and to highlight the variability observed between independent experiments.

Interpretability of the Predictions: Despite using relevance backpropagation, the authors were unable to pinpoint clear human-interpretable image features that drive the predictions. In other words, the deep learning model remains somewhat of a "black box" in terms of what subtle cues it uses at early time points. This limits the biological insight that can be directly extracted regarding early morphological indicators of RPE or lens fate. It would be ideal if the study could highlight specific morphological differences (even if minor) correlated with fate outcomes, but currently those remain elusive.

Response: We thank the reviewer for raising this important point. Indeed, while our models achieved robust predictive performance, the underlying morphological cues remained difficult to interpret using relevance backpropagation. We believe this limitation reflects both the subtlety of the early predictive signals and the complexity of the features captured by deep learning models, which may not correspond to human-intuitive descriptors. We have clarified this limitation in the Discussion and Supplementary Note 1 and emphasize that further methodological advances in interpretability, or integration with complementary molecular readouts, will be essential to uncover the precise morphological correlates of fate determination.

Scope of Outcomes: The study focuses on two particular tissues (RPE and lens) as the outcomes of interest. These were well-chosen as examples (one induced, one spontaneous), but they do not encompass the full range of retinal organoid fates (e.g., neural retina layers). It's not a flaw per se, but it means the platform as presented is specialized. The method might need adaptation to predict more complex or multiple tissue outcomes simultaneously.

Response: We agree with the reviewer that our study focuses on two specific tissues, RPE and lens, which served as proof-of-concept outcomes representing both induced and spontaneous differentiation events. While this scope is necessarily limited, we believe it demonstrates the general feasibility of our approach. We have clarified in the Discussion that the same framework could, in principle, be extended to additional retinal fates such as neural retina layers, or even to multi-label prediction tasks, provided appropriate annotations are available. We now provide additional experiments showing that even abstract morphological classes are well predictable. This will be an important next step to broaden the applicability of our platform.

Requirement of Large Data and Annotations: Practically, the approach required a very large imaging dataset and extensive manual annotation; each organoid's RPE and lens outcome, plus manual masking for training the segmentation model. This is a substantial effort that may be challenging to reproduce widely. The authors suggest that perhaps ~500 organoids might suffice to achieve similar results, but the data requirement is still high. Smaller labs or studies with fewer organoids might not immediately reap the full benefits of this approach without access to such imaging throughput.

Response: We thank the reviewer for highlighting this important point. We agree that the generation of a large imaging dataset and the associated annotations represent a substantial investment of time and resources. At the same time, we consider this effort highly relevant, as it reflects the intrinsic heterogeneity of organoid systems rather than technical artifacts, and therefore ensures robust model training. We have clarified this limitation in the discussion. While our full dataset included ~1,000 organoids, our downsampling analysis suggests that as few as ~500 organoids may already be sufficient to reproduce the key findings, which we believe makes the approach feasible for many organoid systems (compare new Supplementary Note 1). Moreover, as we outline in the Discussion, future refinements such as combining image- and tabular-based features or incorporating fluorescence data could further enhance predictive power and reduce annotation effort.

Medaka Fish vs. Other Systems: The retinal organoids in this study appear to be from medaka fish, whereas much organoid research uses human iPSC-derived organoids. It's not fully clear in the manuscript as to how the findings translate to mammalian or human organoids. If there are species-specific differences, the applicability to human retinal organoids (which are important for disease modeling) might need discussion. This is a minor point if the biology is conserved, but worth noting as a potential limitation.

Response: We thank the reviewer for pointing out this important consideration. We have now explicitly clarified in the Discussion that our proof-of-concept study was performed in medaka organoids, which offer high reproducibility and rapid development. While species-specific differences may exist, the predictive framework is not inherently restricted to medaka and should, in principle, be transferable to mammalian or human iPSC/ESC-derived organoids, provided sufficiently annotated datasets are available. We have amended the Discussion accordingly.

Predicting Tissue Size is Harder: The model's accuracy in predicting how much tissue (relative area) an organoid will form, while good, is notably lower than for simply predicting presence/absence. Final F1 scores for size classes (~0.7) indicate moderate success. This implies that quantitatively predicting organoid phenotypic severity or extent is more challenging, perhaps due to more continuous variation in size. The authors do acknowledge the lower accuracy for size and treat it carefully.

Response: We thank the reviewer for this observation and agree with their interpretation. We have already acknowledged in the manuscript that predicting tissue size is more challenging than predicting tissue presence/absence, and we believe we have treated these results with appropriate caution in the revised version of the manuscript.

Latency vs. Determination: While the authors narrow down the time window of fate determination, it remains somewhat unclear whether the times at which the model reaches high confidence truly correspond to the biological "decision point" or are just the earliest detection of its consequences. The manuscript discusses this caveat, but it's an inherent limitation that the predictive time point might lag the actual internal commitment event. Further work might be needed to link these predictions to molecular events of commitment.

Response: We agree with the reviewer. As noted in the Discussion, the time points identified by our models likely reflect the earliest detectable morphological consequences of fate determination, rather than the exact molecular commitment events themselves. Establishing a direct link between predictive signals and underlying molecular mechanisms will require future experimental work.
